# Nanorings with copper(ii) and zinc(ii) centers: forcing copper porphyrins to bind axial ligands in heterometallated oligomers†
†Electronic supplementary information (ESI) available: Synthesis and characterization of new compounds, UV-vis-NIR titrations and binding data for reference compounds and for the formation of linear oligomer complexes, error analysis and calculation of statistical factors. See DOI: 10.1039/c6sc01809b



**DOI:** 10.1039/c6sc01809b

**Published:** 2016-08-03

**Authors:** Jonathan Cremers, Sabine Richert, Dmitry V. Kondratuk, Tim D. W. Claridge, Christiane R. Timmel, Harry L. Anderson

**Affiliations:** a Department of Chemistry , University of Oxford , Chemistry Research Laboratory , Oxford OX1 3TA , UK . Email: harry.anderson@chem.ox.ac.uk; b Department of Chemistry , University of Oxford , Centre for Advanced Electron Spin Resonance , Oxford OX1 3QR , UK

## Abstract

The affinity of copper(ii) porphyrins for pyridine ligands is extremely weak, but oligo-pyridine templates can be used to direct the synthesis of Cu-containing cyclic porphyrin oligomers when they also have Zn centers. We report the synthesis of two heterometallated nanorings: a six-porphyrin ring prepared from a Zn/Cu/Zn linear trimer and a ten-porphyrin ring prepared from a Zn/Zn/Cu/Zn/Zn pentamer. Both these macrocycles have copper porphyrins at two specific positions across the diameter of the ring and zinc at other sites. The presence of a paramagnetic metal results in broadening of the ^1^H NMR spectra and reduces the relaxation time constants (*T*_1_ and *T*_2_). The changes in *T*_1_ provide quantitative information on the distance of each proton from the copper atom. The Zn/Zn/Cu/Zn/Zn linear porphyrin pentamer binds strongly to a penta-pyridyl template, despite the weakness of the Cu–N interaction, because of the chelate cooperativity of the neighboring Zn–N coordination. The stabilities of a family of four linear porphyrin pentamer complexes were determined by UV-vis-NIR titration and analyzed using a chemical double-mutant cycle. The results show that the free energy of interaction of a copper center to axial pyridine ligands is –6.2 kJ mol^–1^ when the entropy cost of bringing together the two molecules has already been paid by pyridine–zinc interactions. The development of template-directed approaches to the synthesis of nanorings with combinations of different metals at specific positions around the ring opens up many possibilities for controlling the photophysical behavior of these supramolecular systems and for probing their conformations by EPR.

## Abstract 2

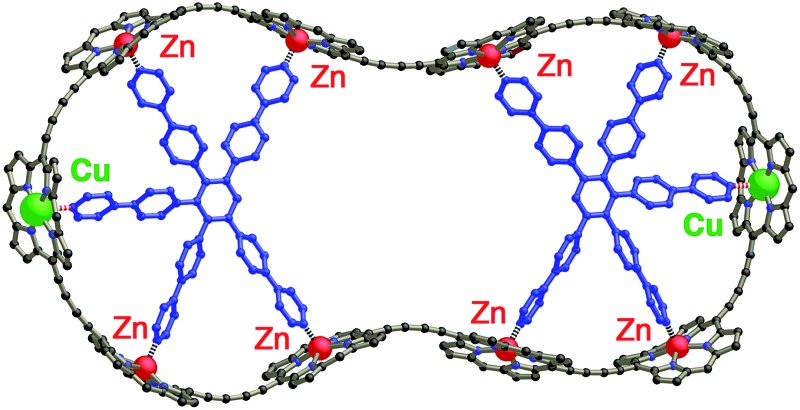
The stability of copper/zinc heterometallated porphyrin complex oligomers provides information on the strength of the copper porphyrin pyridine interaction. Heterometallated nanorings were prepared by template-directed synthesis.

## Introduction

**Fig. 1:**
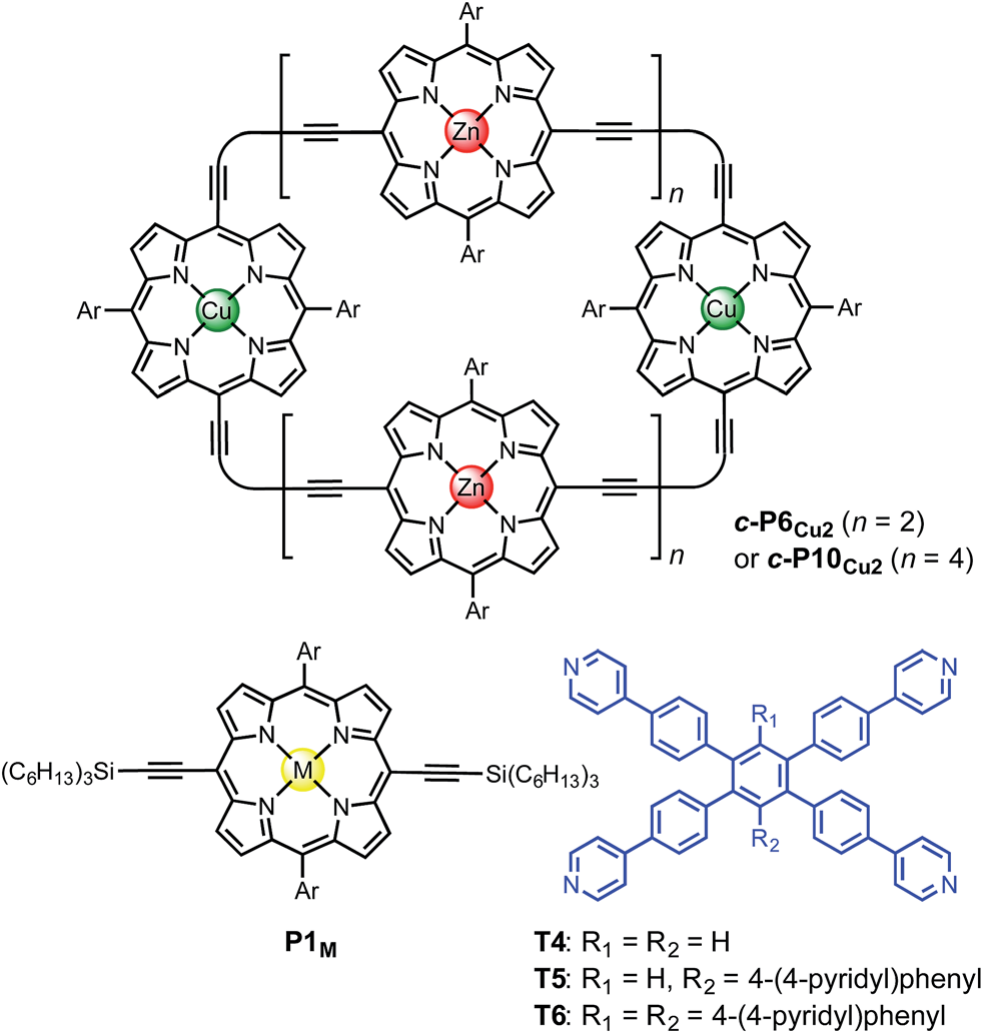
Chemical structures of the porphyrin nanorings ***c*-P6_Cu2_** (*n* = 2) and ***c*-P10_Cu2_** (*n* = 4), **P1_M_** (where M = Zn, Cu, or 2H), and the templates **T4**, **T5** and **T6**. Ar = 3,5-bis(*tert*-butyl)phenyl.

The use of templates to control the formation of covalent bonds, *via* non-covalent interactions, has provided access to many fascinating molecular structures.^1–4^ Various types of reversible interactions can be used in template-directed synthesis. The most versatile of these approaches is arguably the coordination of zinc porphyrins to oligo-amine templates.^5–15^ Zinc porphyrin nanorings exhibit intriguing photophysical and ligand-binding behavior,^12–17^ yet their scope would be dramatically expanded if we could include other transition metal centers. Here we present a strategy for the synthesis of nanorings with copper(ii) porphyrins at two specific positions across the diameter of the macrocycle and zinc at the other sites, ***c*-P6_Cu2_** and ***c*-P10_Cu2_**, using templates **T6** and **T5**, respectively (Fig. 1). This methodology should be applicable for the synthesis of many other heterometallated porphyrin nanorings.

Heterometallated porphyrin oligomers have been widely investigated as model systems for exploring electron transfer,^18–20^ energy transfer,^21–24^ nonlinear optical activity^25^ and molecular recognition.^26–28^ The presence of an open-shell copper(ii) ion in a porphyrin oligomer provides a local fluorescence-quenching site, due to relaxation *via* d–d transitions, although phosphorescence from trip-doublet states is sometimes observed.^29–31^ Thus the quenching of fluorescence from a free-base or zinc porphyrin by a remote copper center provides a test for long-range energy migration.^23^ EPR measurements have been used to probe the structure of DNA strands covalently attached to copper(ii) porphyrin units.^32^ The through-space dipolar coupling between two paramagnetic copper(ii) centers can be measured by EPR using double electron-electron resonance (DEER).^33^ This technique provides accurate information on Cu···Cu distances in the range 2–5 nm.^34^ The ability to place two copper(ii) centers at precisely defined positions in a nanoring enables the conformation and flexibility of the whole assembly to be probed by EPR.^35^

**Fig. 2:**
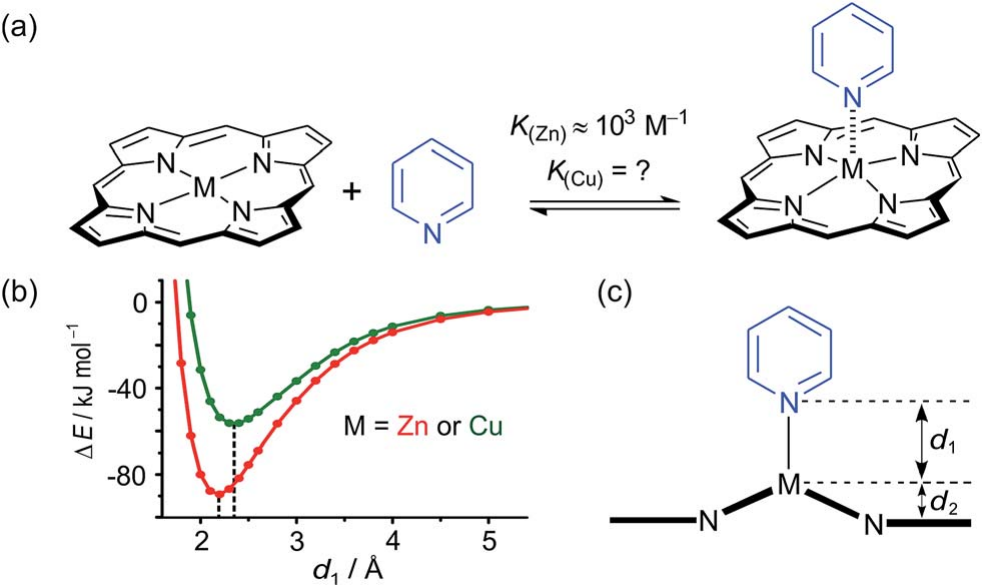
(a) Axial coordination of pyridine to a metalloporphyrin (M = Zn or Cu). (b) Total SCF energy differences as a function of the metal–pyridine separation distance for M = Zn (red) and M = Cu (green). DFT calculations were carried out with Turbomole V6.1 (ref. 42) under *C*_2_ symmetry, DFT/B3LYP with the TZVP basis set,^43^ RI-approximation,^44^ empirical dispersion corrected energies.^45^ (c) Scheme of a metal center (M) bound in a porphyrin coordinating an axial pyridine ligand; *d*_1_ is the distance between the central metal and the axial pyridine, while *d*_2_ is the displacement of the metal atom from the plane of the porphyrin ligand.

While the binding of zinc(ii) porphyrins to amines has been studied extensively,^36,37^ little is known about the coordination of axial ligands to copper(ii) porphyrins (Fig. 2). The interaction appears to be extremely weak, as expected from a consideration of the relevant orbitals. In Cu(ii) (d^9^) complexes, there is strong ligand binding in the equatorial plane, due to a single vacancy in the 3d_*x*^2^–*y*^2^_ orbitals. Bonding in the equatorial plane is also enhanced by hybridization of the 4s and 3d_*z*^2^_ orbitals which leads to a build-up of electron density along the *z*-axis, causing a reduction in axial binding strength. When the d-shell is full, as in Zn(ii), the driving force for bonding in the equatorial plane is reduced, as is the 4s/3d_*z*^2^_ hybridization, and axial ligands bind more strongly.^38^ During the 1950s, Miller and Dorough reported that copper(ii) tetraphenyl porphyrins bind pyridine with an association constant of 0.05 ± 0.02 M^–1^ (in benzene at 303 K).^36^ This value was supported by subsequent studies,^39,40^ but it is not clear whether such a weak interaction can be measured reliably by a simple UV-vis titration, since an association constant of 0.05 M^–1^ requires a pyridine concentration of 20 M for 50% saturation, which is greater than the concentration of pyridine in neat pyridine (12 M). Here we demonstrate that heterometallated linear porphyrin oligomers can be used to measure the weak interaction of a copper center with axial nitrogen ligands, without requiring high concentrations of the pyridine ligand, by utilizing the cooperative effect of neighboring zinc centers in a chemical double-mutant cycle.^41^

Some insights into the interaction of copper and zinc porphyrins with pyridine are provided by DFT calculations.^42–45^ A plot of the self-consistent field (SCF) energy *vs.* distance (*d*_1_) from constrained DFT geometry optimizations at fixed metal–pyridine distances (Fig. 2b) reveals that the binding energy is significantly smaller for copper porphyrins than for zinc. As a result, a shorter equilibrium M–N bond length (*d*_1_) of 2.18 Å is found for M = Zn compared to 2.35 Å for M = Cu. The calculations also show that the zinc atom is pulled out of the porphyrin plane by *d*_2_ = 0.28 Å in the equilibrium geometry, whereas the copper atom is only slightly displaced when it coordinates pyridine, *d*_2_ = 0.12 Å, reflecting stronger equatorial binding. The calculated distances *d*_1_ and *d*_2_ agree well with data from X-ray crystallography. The only known structure of a neutral copper(ii) porphyrin with a 5-coordinate metal bound to a nitrogen-ligand is a pyridine complex reported by Lipstman and Goldberg,^46,47^ whereas the Cambridge Structural Database^48^ contains 362 crystal structures of zinc porphyrin amine complexes. The mean zinc–pyridine distance is *d*_1_ = 2.16(3) Å and the out of plane distance is *d*_2_ = 0.24(6) Å.^9*c*^ The parameters for the Cu(ii) complex are *d*_1_ = 2.47 Å and *d*_2_ = 0.12 Å.^46^ The scarcity of crystallographic data and the length of this Cu–N bond illustrate the weakness of the interaction.

## Results and discussion

### Synthesis of linear porphyrin oligomers

**Scheme 1:**
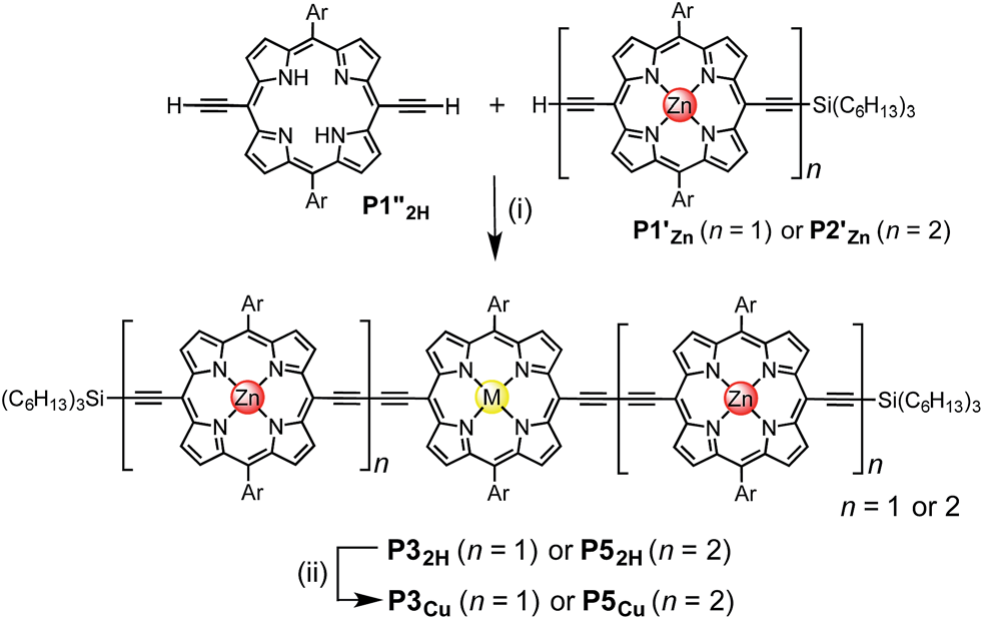
Synthetic route of **P3_Cu_** and **P5_Cu_**. Reaction conditions: (i) Pd_2_(dba)_3_, tri-2-furylphosphine, 1,4-benzoquinone, toluene/Et_3_N, 39%; (ii) Cu(OAc)_2_·H_2_O, CHCl_3_, 95%. Ar = 3,5-bis(*tert*-butyl)phenyl, M = 2H or Cu.

Two linear porphyrin oligomers with a copper porphyrin in the middle of the chain, **P3_Cu_** and **P5_Cu_**, were prepared by coupling the free-base deprotected porphyrin monomer **P1′′_2H_** with a large excess of mono-protected monomer **P1′_Zn_** or dimer **P2′_Zn_**, as shown in Scheme 1 (*n* = 1 and *n* = 2, respectively). Copper was then inserted into the central free-base porphyrin unit at the final stage of the synthesis. This route was adopted because the free-base intermediates have better solubility than the corresponding copper porphyrins, and because it can easily be modified to insert other metals at the centers of the oligomers. Copper-free palladium catalyzed coupling conditions were used to avoid premature insertion of copper into the free-base porphyrin units.^49^ All of the compounds were fully characterized by MALDI-ToF mass spectroscopy, UV-vis-NIR and NMR spectroscopy (see ESI, Section S6†).

### Synthesis of heterometallated porphyrin nanorings

**Scheme 2:**
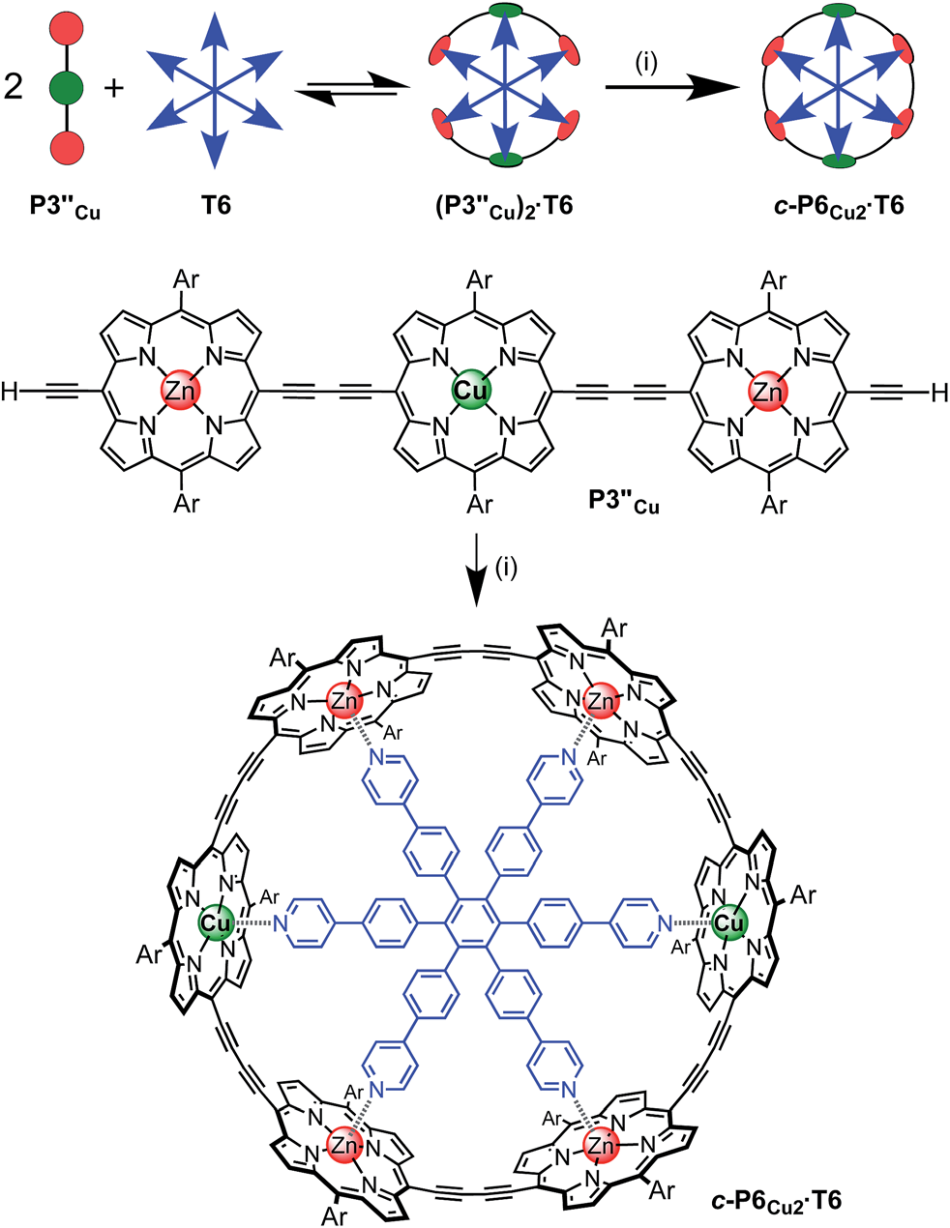
Synthesis of ***c*-P6_Cu2_·T6**. Two representations of the synthetic route used to prepare ***c-*P6_Cu2_·T6**. Reaction conditions: (i) PdCl_2_(PPh_3_)_2_, CuI, 1,4-benzoquinone, i-Pr_2_NH, CHCl_3_, 2%. Ar = 3,5-bis(*tert*-butyl)phenyl.

The six-porphyrin nanoring with two copper centers, ***c*-P6_Cu2_**, was prepared by the oxidative homocoupling of two deprotected trimers, **P3′′_Cu_**, in the presence of the hexadentate template **T6** (Scheme 2). Polymerization predominated in this reaction, and the desired product, ***c*-P6_Cu2_·T6**, was isolated in only 2% yield, reflecting the weak binding of the Zn/Cu/Zn trimer to the **T6** template.^15^

**Scheme 3:**
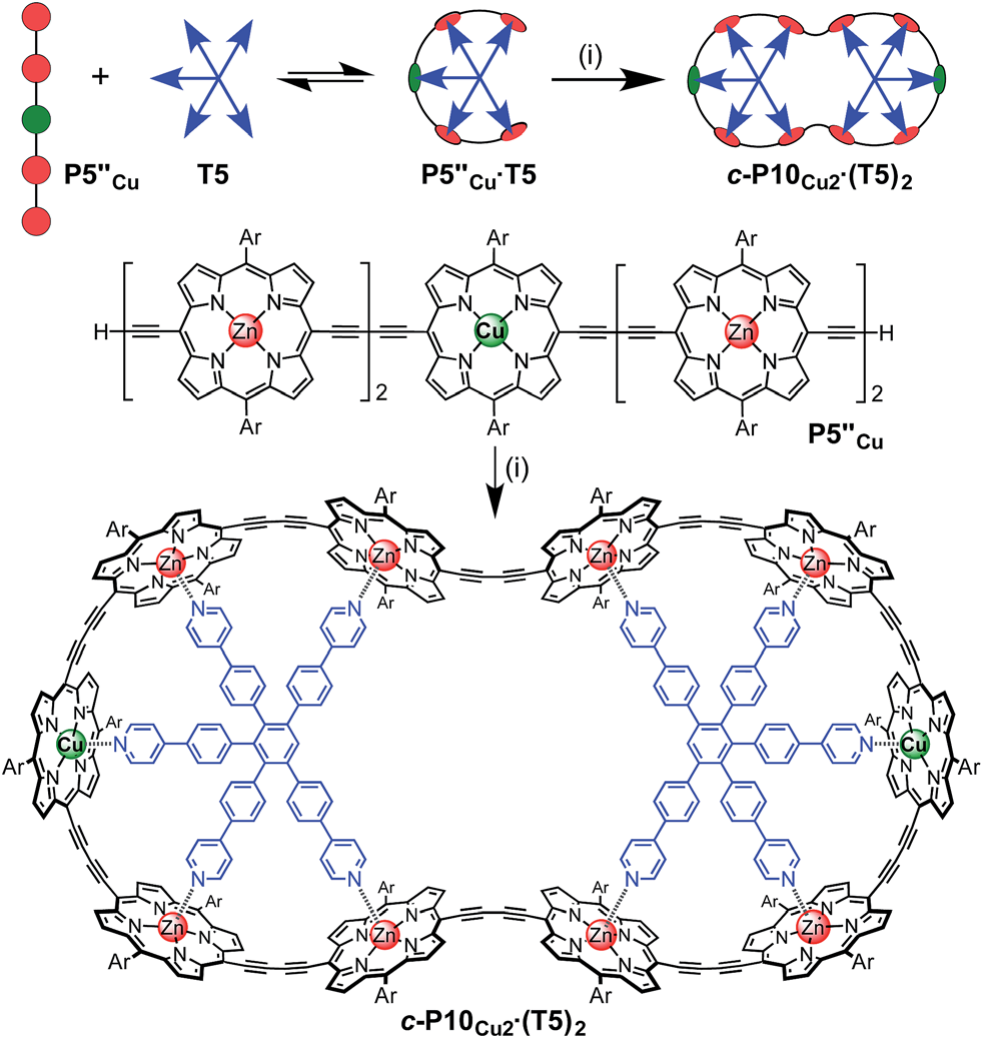
Synthesis of ***c*-P10_Cu2_·(T5)_2_**. Two representations of the synthetic route used to prepare ***c-*P10_Cu2_·(T5)_2_**. Reaction conditions: (i) PdCl_2_(PPh_3_)_2_, CuI, 1,4-benzoquinone, i-Pr_2_NH, CHCl_3_, 17%. Ar = 3,5-bis(*tert*-butyl)phenyl.

The ten-porphyrin nanoring with two copper centers, ***c*-P10_Cu2_**, was prepared using a ‘caterpillar-track’ templating strategy,^12^ by oxidative homocoupling of the deprotected pentamer, **P5′′_Cu_**, in the presence of a pentadentate template **T5** (Scheme 3). In this case, the larger number of zinc binding sites leads to stronger binding of the template and a more efficient synthesis. The desired heterometallated nanoring was isolated as its template-complex ***c*-P10_Cu2_·(T5)_2_** in a 17% yield. The **T5** template was removed from this complex in quantitative yield by addition of excess pyridine.

### NMR spectroscopy of copper-containing oligomers

Many copper porphyrins have been synthesized, but their NMR spectra are rarely reported, because they tend to give broad unresolved resonances.^50,51^ NMR measurements on paramagnetic compounds are often uninformative since the unpaired electron causes rapid relaxation, resulting in broad signals,^52^ however the heterometallated porphyrin oligomers used in this study are large enough to give informative ^1^H NMR spectra, and the effect of the paramagnetic center provides extra information. Protons close to the copper ion give extremely broad peaks, whereas those further from the paramagnetic center are well resolved.

**Fig. 3:**
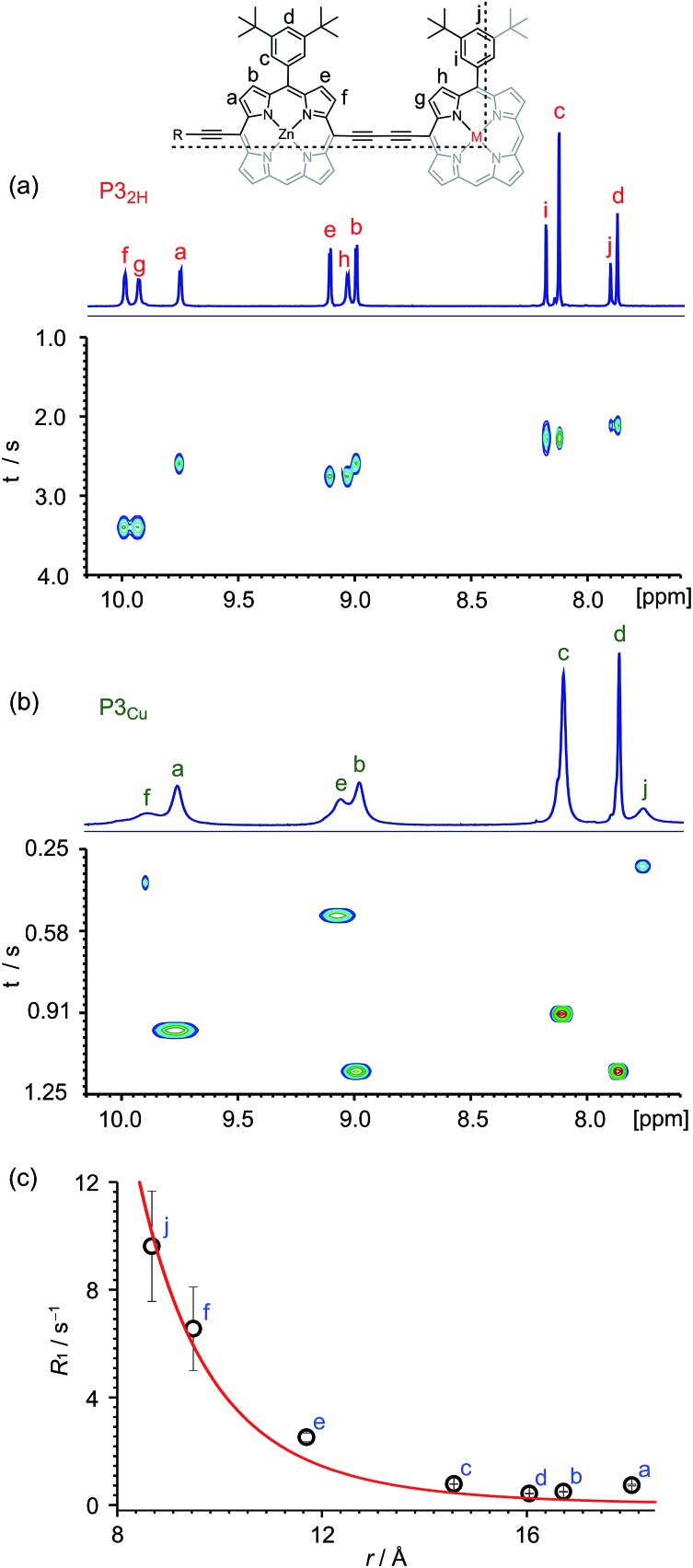
^1^H NMR spectra (CDCl_3_, 700 MHz, 298 K) of **P3_2H_** (a) and **P3_Cu_** (b) and general signal assignment (M = 2H or Cu). Only the aromatic regions of the spectra are shown. The graphs in (a) and (b) correlate the chemical shift and the corresponding *T*_1_ relaxation time of each proton signal. (c) Graph depicts the change in *R*_1_ relaxation rates (*R*_1_ = 1/*T*_1_) between **P3_Cu_** and **P3_2H_** with respect to the distance between the two nuclei, with a fit for Δ*R*_1_ ∝ *r* ^–6^. The experimental error in Δ*R*_1_ is only evident when *T*_1_ is short (signals j and f).

The aromatic regions of the ^1^H NMR spectra of the linear trimers containing either a free-base, **P3_2H_**, or a copper porphyrin, **P3_Cu_**, are compared in Fig. 3. The NMR spectrum of the diamagnetic compound **P3_2H_** shows sharp signals for both the β-protons (at 8.9–10.0 ppm) and the aryl-protons (at 7.8–8.2 ppm). In the spectrum of the copper-containing trimer, **P3_Cu_**, the signals for the β-protons nearest to the copper (g and h) are completely unobservable, whereas the signals further removed from the copper (a–d) are well resolved.

The deterioration observed in the ^1^H NMR spectrum of **P3_Cu_** is a direct result of the presence of the paramagnetic copper center and can be attributed to shortening of the *T*_1_ and *T*_2_ relaxation time constants. Values of *T*_1_ and *T*_2_ were determined for **P3_Cu_** and **P3_2H_** using the inversion-recovery and Carr–Purcell–Meiboom–Gill (CPMG) sequences, respectively.^53^ As expected, analysis of the data for the signals a–j in **P3_2H_** shows little variation; the *T*_1_ times are in the range 2–3 s (Fig. 3a) and the *T*_2_ times are 0.05–0.78 s (see ESI, Fig. S36†). These values fall in the range expected for molecules of this size and reflect the local environment of the protons within the molecule. As a result of the paramagnetic copper center, **P3_Cu_** has considerably shorter relaxation times; *T*_1_ = 0.10–1.12 s (Fig. 3b) and *T*_2_ = 0.005–0.083 s for signals a–j (see ESI, Fig. S36†). Many factors contribute to relaxation rates, but a clear trend is observed when comparing oligomers with and without copper. The relaxation rates are considerably faster for **P3_Cu_** than for **P3_2H_** indicating that all protons are affected by the copper center. Additionally, the relaxation rates are noticeably greater for the signals in closer spatial proximity to the copper center in **P3_Cu_**, allowing us to estimate the relative distances between the protons and the copper center based on their relaxation times. The difference in relaxation rate *R*_1_ between protons in **P3_Cu_** and **P3_2H_** can be defined as Δ*R*_1_ according to eqn (1).1
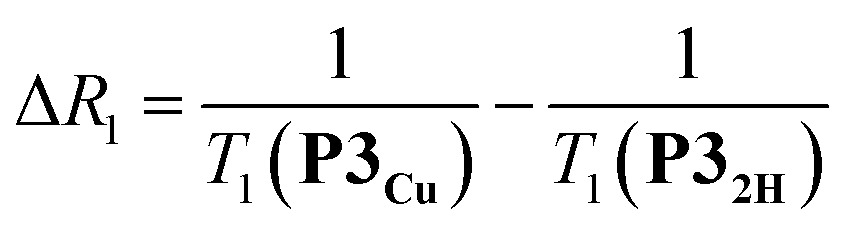



Dipolar relaxation rates are expected to depend on the inverse 6^th^ power of the distance between two magnetic dipoles, eqn (2).^54^
2
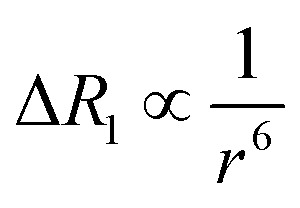



Fig. 3c shows a plot of the experimental values of Δ*R*_1_ against the distance (*r*) between the copper center and the corresponding proton, estimated from crystal structures of similar oligomers.^55^ The good fit to eqn (2) confirms the expected distance dependence and shows that changes in *T*_1_ relaxation rate can be used to gain structural information.

The NMR spectra of the porphyrin pentamer **P5_Cu_** are more complicated than those of **P3_Cu_**, because there are twice as many zinc porphyrin environments (see ESI, Section S3†). Protons further from the copper center give rise to sharp signals, but many of the signals overlap so *T*_1_ and *T*_2_ were not measured for these oligomers.

### Quantification of the Cu–porphyrin pyridine interaction

**Fig. 4:**
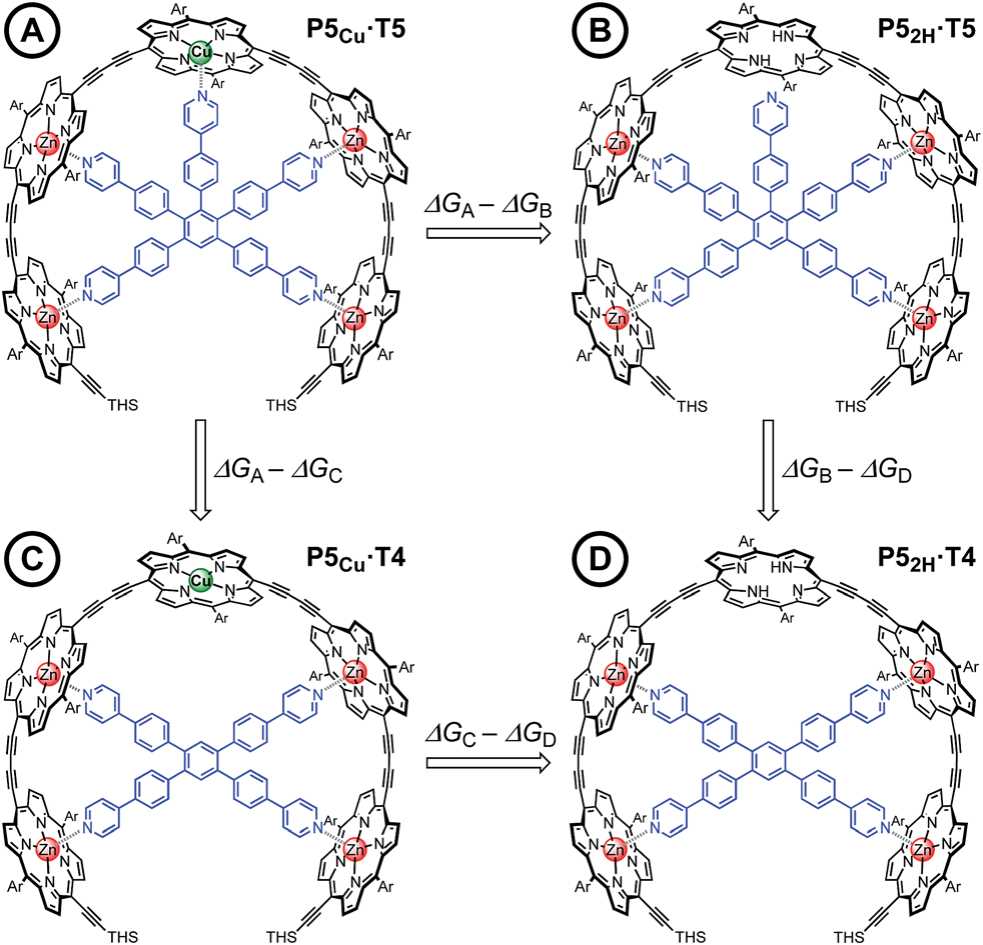
The chemical DMC used to investigate the interaction between the copper center in the central porphyrin and the pyridine leg of template **T5**. Ar = 3,5-bis(*tert*-butyl)phenyl, THS = trihexylsilyl.

Chemical double-mutant cycles (DMCs) are a way to probe weak, non-covalent interactions by utilizing the cooperative binding effect of stronger neighboring interactions.^41,56^ This approach allows one to disentangle the free energy contribution due to chelate cooperativity associated with the formation of intramolecular non-covalent interactions. We envisioned that we could utilize the cooperative binding effect of the four zinc porphyrins in **P5_Cu_** to force the copper center to interact with the template and determine its contribution to the binding strength. The DMC illustrated in Fig. 4 quantifies and eliminates all secondary and allosteric effects associated with single mutations and provides a measure of the free energy benefit associated with the Cu···N interaction by comparing the affinities of **T4** and **T5** for **P5_2H_** and **P5_Cu_**.

Simply comparing the stabilities of **P5_Cu_·T5** and **P5_2H_·T5** (complexes A and B respectively) would give an estimate of the energy of the Cu···N interaction, but this approach could lead to false conclusions because a mutation in one part of the molecule (**P5_Cu_** → **P5_2H_**) may influence other interactions; for example, a free-base porphyrin might be more flexible than a copper porphyrin, which would add to the stability of **P5_2H_·T5**, or there might be a contact between the pyridine ligand and the free-base porphyrin which could destabilize **P5_2H_·T5**. Similar issues apply to a simple comparison of complexes A and C, where a single mutation is made in the ligand (**T5** → **T4**). The DMC approach overcomes these problems by cancelling the secondary free energy effects of the mutations in a pairwise fashion in the thermodynamic cycle.^41^ The cycle is completed by probing the stability of complex D (**P5_2H_·T4**), in which both the copper center and the central binding leg are removed.

**Fig. 5:**
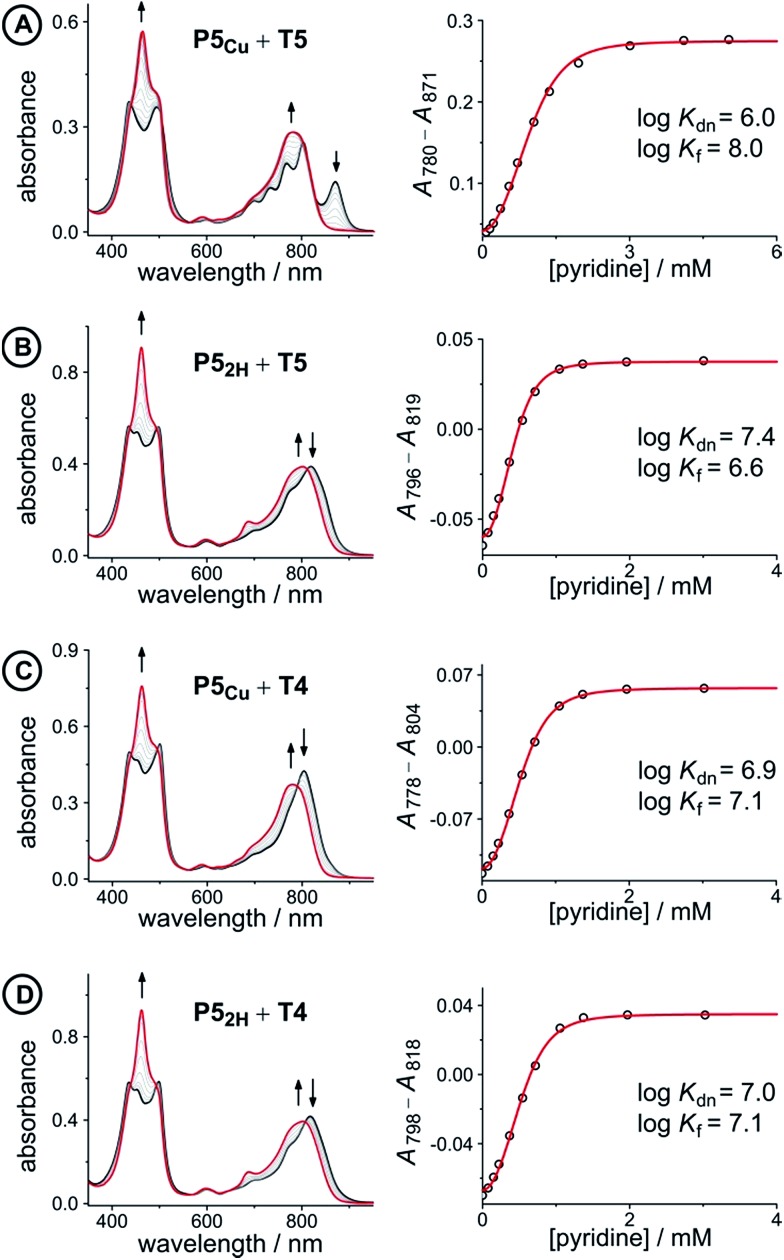
UV-vis-NIR denaturation titrations of linear porphyrin oligomer complexes with templates **T4** and **T5** (all in CHCl_3_ at 298 K). The spectra shown on the left are those of the 1 : 1 complexes (black) and the end spectra of the pyridine-saturated oligomers (red). On the right the experimental (black circles) and calculated binding isotherms (red lines) are shown. All titrations were performed at least twice (see ESI, Section S1.2†).

The 1 : 1 complexes shown in Fig. 4 were generated by titrating solutions of the corresponding porphyrin pentamers dissolved in chloroform with the ligands **T5** or **T4**. These UV-vis-NIR titrations gave sharp end points, and the complexes are too stable for their formation constants to be determined directly from their formation curves. Therefore, denaturation titrations were performed to determine the formation constant *K*_f_ *via* the denaturation constant *K*_dn_.^14,15,57^ A large excess of pyridine was titrated into solutions of the 1 : 1 complexes (*ca.* 10^–6^ M in CHCl_3_ at 298 K) to displace the multidentate ligands. Our analysis assumes that the denaturation processes are essentially all-or-nothing two state equilibria (*i.e.* that intermediate partially denatured species do not build up to significant concentration). This assumption is supported by the isosbestic nature of the UV-vis-NIR titrations and by the good fits of the curves to the calculated binding isotherm for a two-state equilibrium (Fig. 5). Denaturation constants were used to calculate the formation constants *K*_f_ using eqn (3):3
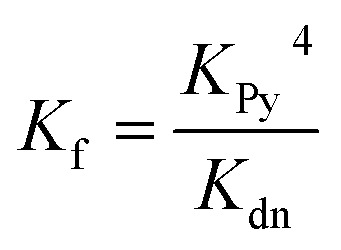
where *K*_Py_ is a reference binding constant which was approximated to the binding of pyridine to porphyrin monomer **P1_Zn_** (see ESI, Fig. S2–4;† *K*_Py_ = 3.2 × 10^3^ M^–1^ in CHCl_3_ at 298 K).^58^

The denaturation titrations of the complexes with pyridine are illustrated in Fig. 5 and the results are summarized in Table 1. The absorption spectra of the complexes with multidentate ligands are more red-shifted (black lines) than the final spectra (red lines) in which the complex is completely denaturated. The observed red-shift is caused by the more rigid structures and reduced porphyrin–porphyrin dihedral angles in the template complexes, compared to the free oligomers. The absorption spectrum of the template complex **P5_Cu_·T5** (A) clearly reveals more structure in the Q-bands compared to **P5_2H_·T5** (B), **P5_Cu_·T4** (C), and **P5_2H_·T4** (D), which is further evidence for the interaction of the copper center with the template. Binding to **T5** locks every porphyrin unit in **P5_Cu_** into position and the rotational freedom of the central copper porphyrin is lost, giving rise to the observed characteristic fine structure in the absorption spectrum. In the complexes **P5_2H_·T5** (B), **P5_Cu_·T4** (C), and **P5_2H_·T4** (D), motion of the central porphyrin is less restricted resulting in a broader absorption band. The spectral changes observed in the denaturation of complexes B–D are similar because in all cases there is no interaction between the central porphyrin and the template.

**Table 1:** Equilibrium constants and free energy changes from the titrations in Fig. 5

Complex (X)	*K*_dn_ (M^–3^)	log *K*_f(X)_	Δ*G*_X_ (kJ mol^–1^)
**P5_Cu_·T5** (A)	9.1 ± 2.0 × 10^5^	8.0 ± 0.1	–35.6 ± 0.3
**P5_2H_·T5** (B)	2.4 ± 0.3 × 10^7^	6.6 ± 0.1	–29.2 ± 0.2
**P5_Cu_·T4** (C)	8.1 ± 0.3 × 10^6^	7.1 ± 0.1	–30.2 ± 0.2
**P5_2H_·T4** (D)	9.0 ± 0.3 × 10^6^	7.0 ± 0.1	–29.9 ± 0.2

The stability constants of complex **P5_Cu_·T4** (C) and **P5_2H_·T4** (D) are nearly identical, indicating that there is no inductive effect on the binding interaction due to the presence of copper and that the flexibility of copper and free-base porphyrins are comparable. A slightly lower stability constant is found for complex **P5_2H_·T5** (B), which might reflect steric repulsion of the central template leg with the central free-base unit of the oligomer. The formation constant of the complex **P5_Cu_·T5** (A) is roughly an order of magnitude higher than the others, due to interaction of the copper center with the template.

The energy of the copper–pyridine interaction (ΔΔ*G*_Cu_) was calculated from eqn (4):

4 ΔΔ*G*_Cu_ = Δ*G*_A_ – Δ*G*_B_ – Δ*G*_C_ + Δ*G*_D_

where Δ*G*_X_ is the statistically corrected energy of formation of complex X calculated according to eqn (5); *K*_chem(X)_ is the statistically corrected formation constant and *K*_σ(X)_ is the statistical factor of complex X (see ESI, Section 2†).^59^
5
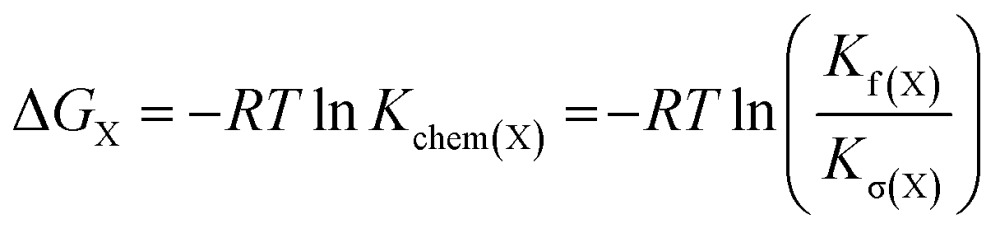



Analysis of these data reveals that the energy of interaction between the copper center and the template is ΔΔ*G*_Cu_ = –6.2 ± 0.4 kJ mol^–1^. This interaction energy is substantially less than the energy of the copper porphyrin pyridine interaction from DFT calculation (56.6 kJ mol^–1^, Fig. 2), which is not surprising as the DFT calculations do not take account of solvation. The free energy changes associated with single mutations Δ*G*_A_ – Δ*G*_B_ = –6.4 ± 0.3 kJ mol^–1^ and Δ*G*_A_ – Δ*G*_C_ = –5.4 ± 0.3 kJ mol^–1^ differ significantly from the value from the double-mutant cycle, showing the benefit of the DMC approach. Its use in this system provided clearer insights into the weak binding interaction than would have been achieved from single mutations.

The free energy term ΔΔ*G*_Cu_ = –6.2 ± 0.4 kJ mol^–1^ is an estimate of the enthalpy of the copper–pyridine interaction, because it is measured in a situation where the loss of translational entropy of bringing together two molecules has already been paid, and the interaction probed by the DMC is effectively intramolecular. In general, the entropy cost of bringing two molecules together to form a non-covalent complex in solution at 298 K contributes approximately –*T*Δ*S* ≈ +6 kJ mol^–1^ to Δ*G*,^60,61^ which would correspond to a free energy for a bimolecular copper porphyrin pyridine interaction of Δ*G*_Cu_ ≈ 0 (*i.e. K* ≈ 1 M^–1^). However there are reports that the coordination of pyridine to a metalloporphyrin is more entropically unfavorable which would explain the very weak association constant.^40,62^

When two molecules bind together through more than one point of interaction, the increased stability resulting from chelate cooperativity can be quantified by the effective molarity (EM).^57,63^ Comparison of the stability constants of **P5_Zn_·T5** (*K*_chem_ = 1.1 ± 0.2 × 10^12^ M^–1^) with those of **P5_2H_·T5** (*K*_chem_ = 1.3 ± 0.2 × 10^5^ M^–1^) and **P1_Zn_** with 4-phenylpyridine (*K*_chem_ = 2.1 ± 0.1 × 10^3^ M^–1^) indicates that the effective molarity of the central Zn–N interaction in **P5_Zn_·T5** is EM = 4 ± 1 × 10^3^ M (see ESI, Section S1.3†). If we assume that the effective molarity is the same for the central Cu–N interaction in **P5_Cu_·T5**, then the single-site microscopic binding constant for copper porphyrins to pyridyl ligands, *K*_Cu_, can be estimated by dividing the observed equilibrium constant (ΔΔ*G*_Cu_ = –6.2 kJ mol^–1^ ⇒ *K*_Cu_EM = 12) by the effective molarity, giving *K*_Cu_ = 3.1 × 10^–3^ M^–1^. This value illustrates how a high effective molarity enables very weak interactions to be measured.

## Conclusions

We have demonstrated that linear porphyrin oligomers can be prepared containing a central free-base porphyrin unit and zinc at the other sites. The free-base porphyrin can be metallated with copper(ii), without transmetallation at the zinc centers, to prepare heterometallated linear oligomers, which are precursors to mixed-metal nanorings.

^1^H NMR spectroscopy can be used to gain structural information on porphyrin oligomers containing paramagnetic copper(ii) centers. While the signals corresponding to protons in close proximity to the copper are broadened to the extent that they can no longer be observed, protons further from the copper are well resolved. The changes in proton relaxation rate constants (*R*_1_ = 1/*T*_1_) due to the presence of the copper center depend on the inverse 6^th^ power to the distance, providing information on the molecular geometry.

The axial binding interaction between a copper porphyrin and pyridine can be quantified with the help of a chemical double-mutant cycle, revealing that the energy of the Cu···N interaction is ΔΔ*G*_Cu_ = –6.2 ± 0.4 kJ mol^–1^. It is tempting to compare this energy with that for coordination of pyridine to a zinc porphyrin monomer (Δ*G*_Zn_ = –20.0 ± 0.2 kJ mol^–1^ for **P1_Zn_** in CHCl_3_ at 298 K). However such a comparison is misleading because Δ*G*_Zn_ includes the loss of translational entropy associated with bringing two molecules together, whereas ΔΔ*G*_Cu_ is measured in a situation where the interaction is effectively intramolecular with a high effective molarity.

The heterometallated porphyrin nanoring complexes ***c*-P6_Cu2_·T6** and ***c*-P10_Cu2_·(T5)_2_** have been prepared by template-directed synthesis. The molecular geometry and metal ligand interactions in the nanoring ***c*-P10_Cu2_** have been investigated using EPR, the results of this study are presented in an accompanying paper.^35^
