# Supplementary material for: Nanorings with copper(ii) and zinc(ii) centers: forcing copper porphyrins to bind axial ligands in heterometallated oligomers
Source: Chem Sci. 2016 Aug 3;7(12):6961–8. doi: 10.1039/c6sc01809b (PMC5355962; doi:10.1039/c6sc01809b)
Supplement: Supplementary file 1 [file SC-007-C6SC01809B-s001.pdf]

Supplementary Electronic Information

Nanorings with Copper(II) and Zinc(II) Centers: Forcing Copper Porphyrins to Bind Axial Ligands in Heterometallated Oligomers

Jonathan Cremers,† Sabine Richert, ‡ Dmitry V. Kondratuk,† Tim D. W. Claridge,†
Christiane R. Timmel,‡ and Harry L. Anderson*,†

† Department of Chemistry, University of Oxford, Chemistry Research Laboratory, Oxford, OX1 3TA

‡ Department of Chemistry, University of Oxford, Centre for Advanced Electron Spin Resonance, Oxford, OX1 3QR

* harry.anderson@chem.ox.ac.uk

Table of Contents

S1. **UV-vis-NIR Titrations** S2

S1.1 Titrations of Porphyrin Monomer **P1**$_{\mathbf{Zn}}$ and Monodentate Ligands S2

S1.2 Denaturation Titrations with Pyridine on Heterometallated Oligomers S4

S1.3 Denaturation of **P5**$_{\mathbf{Zn}}$**·T5** with Pyridine S9

S2. **Calculation of Statistical Factors** S11

S3. **^{1}H NMR Spectra of Linear Pentamers** S14

S4. **DFT Calculations** S14

S5. **T_1 and T_2 Relaxation Time Constant Measurements** S16

S5.1 T_1 Measurements on **P3**$_{\mathbf{2H}}$ S16

S5.2 T_2 Measurements on **P3**$_{\mathbf{2H}}$ S18

S5.3 T_1 Measurements on **P3**$_{\mathbf{Cu}}$ S20

S5.4 T_2 Measurements on **P3**$_{\mathbf{Cu}}$ S22

S5.5 T_1 and T_2 decay rate versus distance plots S24

S6. **Experimental Procedures** S25

S7. **Spectra Confirming Identity of New Compounds** S32

S8. **^{1}H NMR spectra of the 1:1 complexes of P5$_{\mathbf{Cu}}$ and P5$_{\mathbf{2H}}$ with T4 and T5** S43

S9. **References** S44

Section S1. UV-vis-NIR Titrations

Section S1.1. Titrations of Porphyrin Monomer $P1_{Zn}$ and Monodentate Ligands

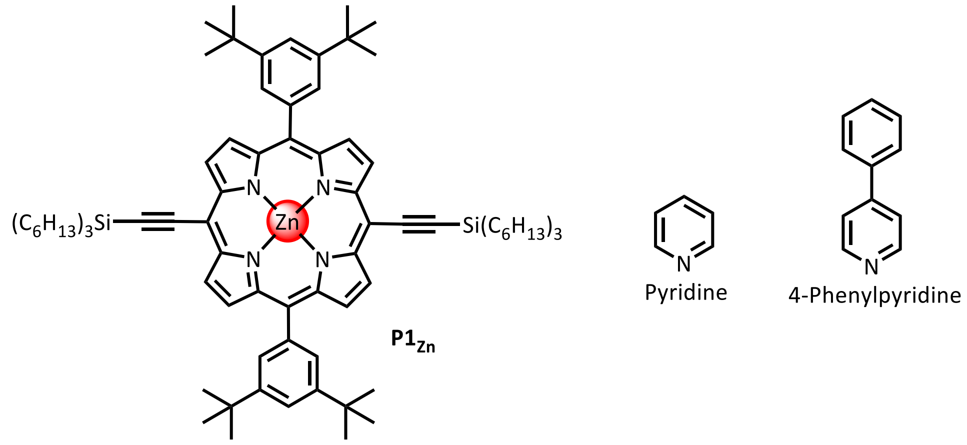

Figure S1: Structures of reference porphyrin monomer $P1_{Zn}$ and monodentate ligands.

Titrations with porphyrin monomer $\mathbf{P1_{Zn}}$ and pyridine or 4-phenylpyridine were performed in order to determine reference binding constants (K_{py} and K_{Ph}).

All titrations were performed in chloroform (containing ca. 0.5% ethanol as stabilizer) at 298 K. Care was taking to keep the porphyrin concentration constant throughout the entire titration by adding porphyrin to the ligand solution before titrations were started. The binding curves were fitted using a 1:1 binding isotherm using the equation:

$$\frac{A - A_{initial}}{A_{\infty} - A_{initial}} = \left(\frac{(K_a([L] + [P]_0) + 1) - \sqrt{(K_a([L] + [P]_0) + 1)^2 - 4K_a^2[P]_0[L]}}{2K_a[P]_0} \right)$$

where *A* is the observed absorption at a specific wavelength or the difference of absorbance between two wavelengths; $A_{initial}$ is the starting absorption at this wavelength; A_{∞} is the asymptotic final absorption at this wavelength; K_a is the association constant between ligand and porphyrin host; [L] is the concentration of ligand; $[P]_0$ is the concentration of porphyrin host. The free variables which were adjusted to optimize the fit to the experimental data during the fitting procedure are $A_{initial}$, A_{∞}, and K_a. Fitting analysis was carried out using the Origin software (Figures S2–S7) giving K_{py} = (3.26 ± 0.25) × 10^3 M^{-1} and K_{Ph} = (4.25 ± 0.12) × 10^3 M^{-1}.

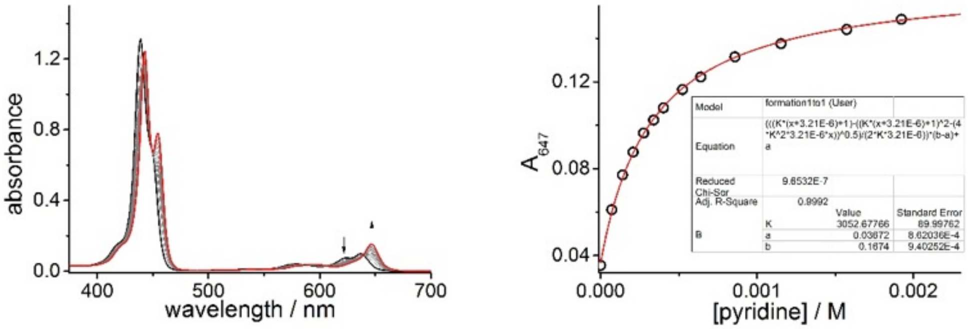

Figure S2: UV-vis titration of $P1_{Zn}$ and pyridine, R^2 = 0.9992. (Run 1, $CHCl_3$, 298 K, [$P1_{Zn}$] = 3.21 μM, K = 3.05 × 10^3 M^{-1}).

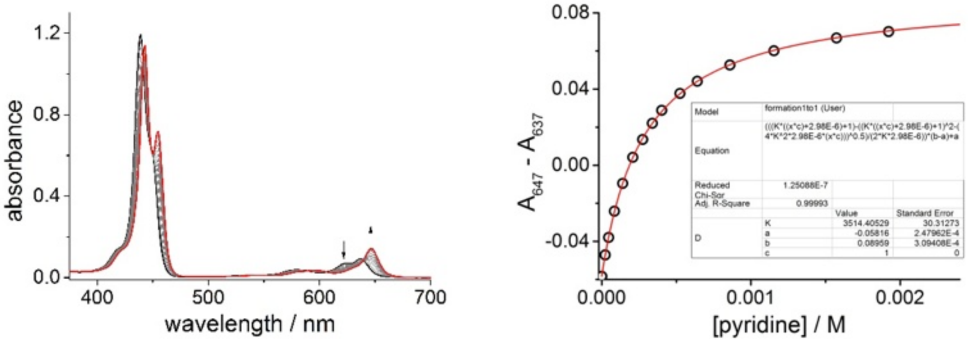

Figure S3: UV-vis titration of $P1_{Zn}$ and pyridine, R^2 = 0.9992. (Run 2, $CHCl_3$, 298 K, [$P1_{Zn}$] = 2.98 μM, K = 3.51 × 10^3 M^{-1}).

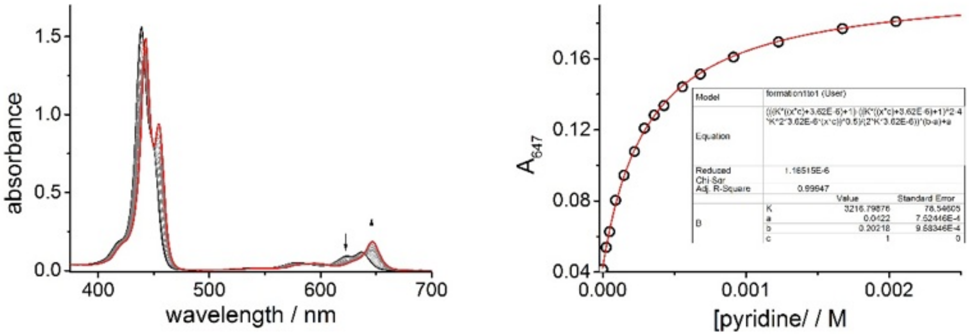

Figure S4: UV-vis titration of $P1_{Zn}$ and pyridine, R^2 = 0.9995. (Run 3, $CHCl_3$, 298 K, [$P1_{Zn}$] = 3.62 μM, K = 3.22 × 10^3 M^{-1}).

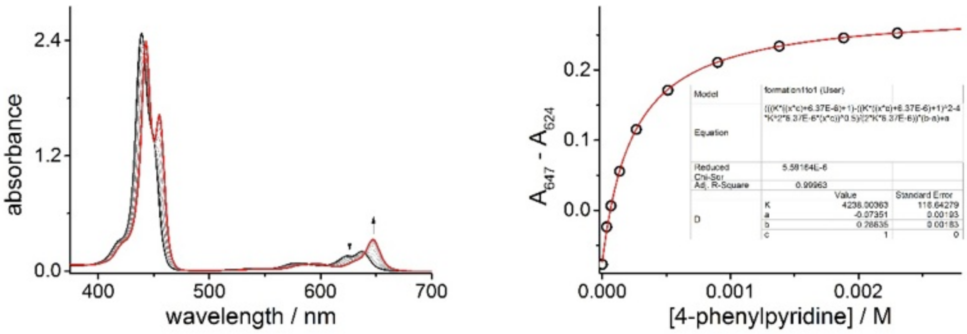

Figure S5: UV-vis titration of $P1_{Zn}$ and 4-phenylpyridine, R^2 = 0.9996. (Run 1, $CHCl_3$, 298 K, [$P1_{Zn}$] = 6.37 μM, K = 4.24 × 10^3 M^{-1}).

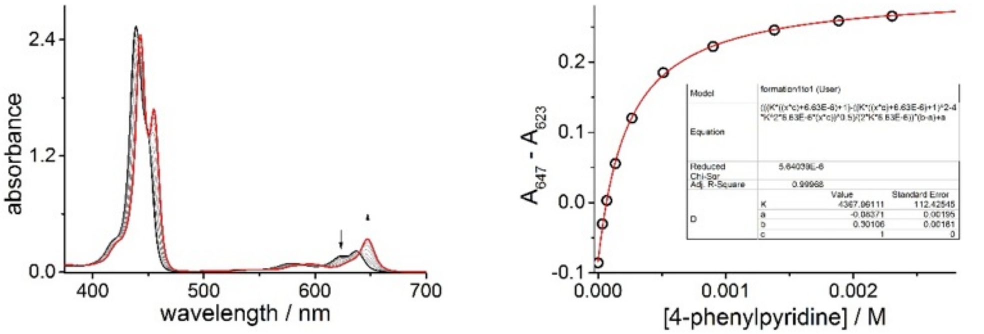

Figure S6: UV-vis titration of $P1_{Zn}$ and 4-phenylpyridine, R^2 = 0.9997. (Run 2, $CHCl_3$, 298 K, [$P1_{Zn}$] = 6.63 µM, $K = 4.37 \times 10^3$ M^{-1}).

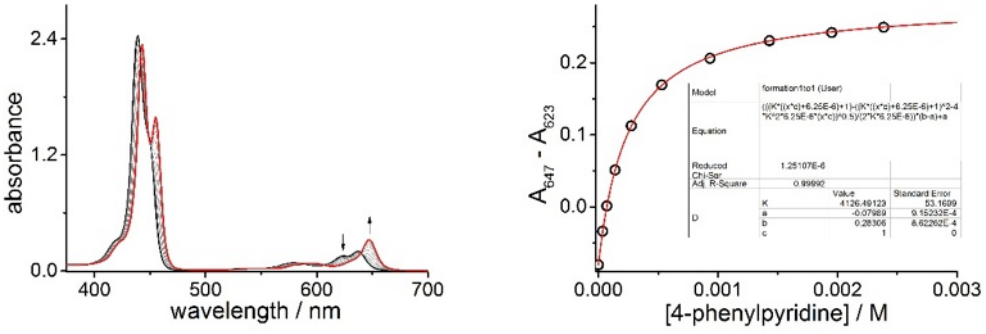

Figure S7: UV-vis titration of $P1_{Zn}$ and 4-phenylpyridine, R^2 = 0.9996. (Run 3, $CHCl_3$, 298 K, [$P1_{Zn}$] = 6.25 µM, $K = 4.13 \times 10^3$ M^{-1}).

Section 1.2. Denaturation Titrations with Pyridine on Heterometallated Oligomers

When binding strength increases, the binding curves become increasingly square, leading to greater uncertainty in the fit. In order to derive a trustworthy binding constant, denaturation titrations (break-up titration) need to be performed with a competing ligand such as pyridine. Using the data from these break-up titrations (K_{dn} = denaturation constant) and the formation constant of the single site binding event of the competing ligand with a zinc-porphyrin monomer (K_{Py} = association constant for pyridine to **$P1_{Zn}$**) allows us to derive the formation binding constant (K_f) between the oligomers (N = number of zinc porphyrin binding sites) and the template using the following equation:

$$K_{\mathrm{f}} = \frac{K_{\mathrm{py}}^{N}}{K_{\mathrm{dn}}}$$

via the thermodynamic cycle shown in Figure S8.

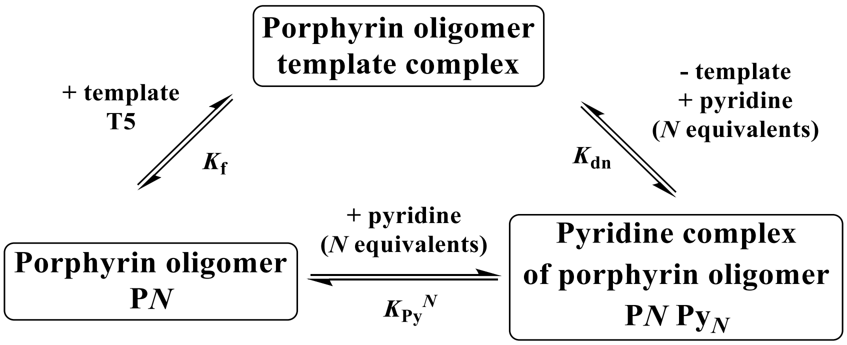

Figure S8: Thermodynamic cycle relating the formation constant of the template complex (K_f) to the denaturation constant (K_{dn}) and binding constant of each porphyrin unit for pyridine (K_{py}).

All the linear pentamers were found to interact strongly with the templates **T5** and **T4** and therefore denaturation titrations were performed on these complexes in order to determine binding constants.

Denaturation titrations were performed in chloroform at 298 K. The 1:1 complexes between the porphyrin oligomers and templates were prepared in the cuvette prior to the denaturation titration. All formation titrations were carried out at constant porphyrin concentrations by adding porphyrin to the ligand (**T5** or **T4**) stock solution and titrating until a 1:1 complex was formed according to UV. All denaturation titrations were carried out at constant porphyrin·template complex concentration by adding both porphyrin and template to the ligand (pyridine) stock solution before titrations started.

Data were fitted to the *N*-dentate denaturation binding isotherm described in the following equation:

$$\frac{A - A_{initial}}{A_\infty - A_{initial}} = \left(\frac{-K_{dn}[L]^N + \sqrt{K_{dn}^2[L]^{2N} + 4K_{dn}[L]^N[P]_0}}{2[P]_0} \right)$$

where *A* is the observed absorption at a specific wavelength or difference of absorption between two wavelengths; $A_{initial}$ is the starting absorption at a specific wavelength or difference between absorption in two wavelengths; A_∞ is the terminal absorption at a specific wavelength or difference of absorption in two wavelengths; K_{dn} is the dissociation constant between ligand and porphyrin oligomer complex, [L] is the concentration of ligand; $[P]_0$ is the concentration of porphyrin oligomer complex, *N* is the number of binding sites in the complex (e.g. *N* = 4 in **$P5_{Cu}$·T4**). The titration curves and fittings are shown below.

Table S1: Results from UV-vis-NIR titrations in Figures S9–S16

	K_{dn} (M^{-3})	K_f (M^{-1})	K_σ	K_{chem} (M^{-1})
**$P5_{Cu}$·T5**	$9.07 \pm 1.99 \times 10^5$	$1.10 \pm 0.29 \times 10^8$	64	$1.72 \pm 0.45 \times 10^5$
**$P5_{2H}$·T5**	$2.44 \pm 0.26 \times 10^7$	$4.10 \pm 0.71 \times 10^6$	32	$1.28 \pm 0.22 \times 10^5$
**$P5_{Cu}$·T4**	$8.08 \pm 0.27 \times 10^6$	$1.24 \pm 0.17 \times 10^7$	64	$1.93 \pm 0.27 \times 10^5$
**$P5_{2H}$·T4**	$8.95 \pm 0.26 \times 10^6$	$1.12 \pm 0.16 \times 10^7$	64	$1.74 \pm 0.25 \times 10^5$

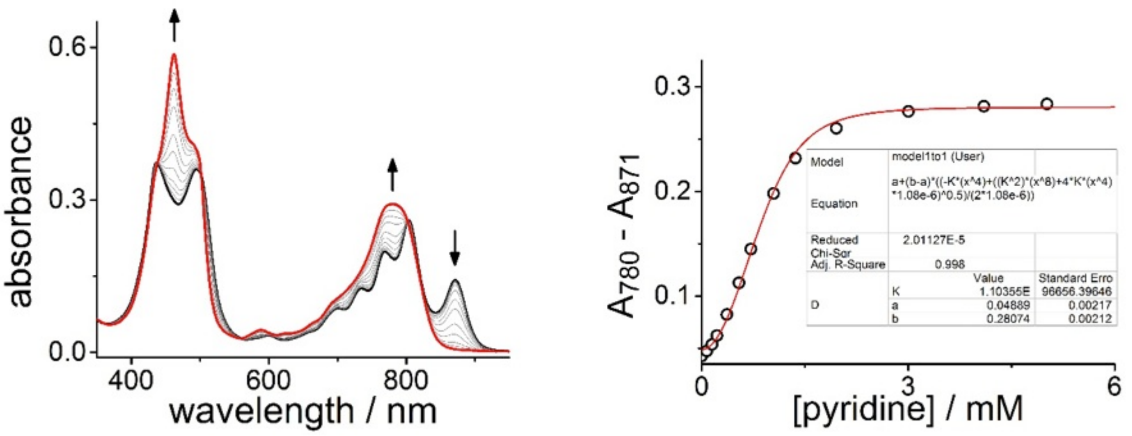

Figure S9: UV-vis-NIR titration of pyridine and P5$_{Cu}$·T5. R^2 = 0.9980. (Run 1, $CHCl_3$, 298 K, [P5$_{Cu}$·T5] = 1.08 µM, K = 1.10 × 10^6 M^{-3}).

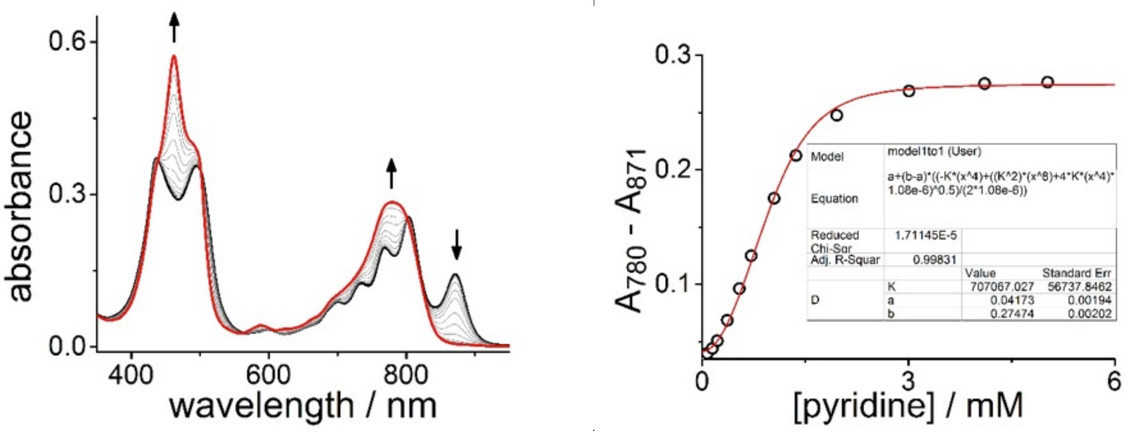

Figure S10: UV-vis-NIR titration of pyridine and P5$_{Cu}$·T5. R^2 = 0.9983. (Run 2, $CHCl_3$, 298 K, [P5$_{Cu}$·T5] = 1.08 µM, K = 7.07 × 10^5 M^{-3}).

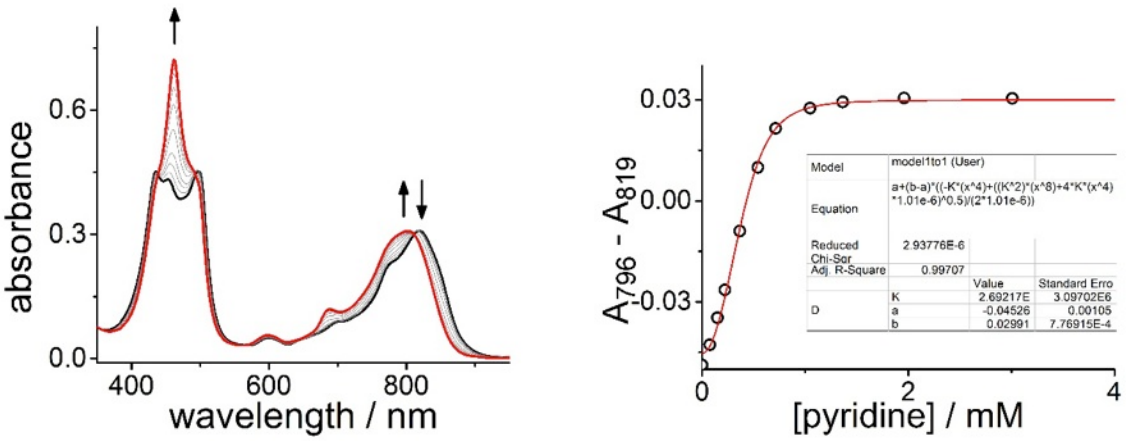

Figure S11: UV-vis-NIR titration of pyridine and P5$_{2H}$·T5. R^2 = 0.9971. (Run 1, $CHCl_3$, 298 K, [P5$_{2H}$·T5] = 1.01 µM, K = 2.69 × 10^7 M^{-3}).

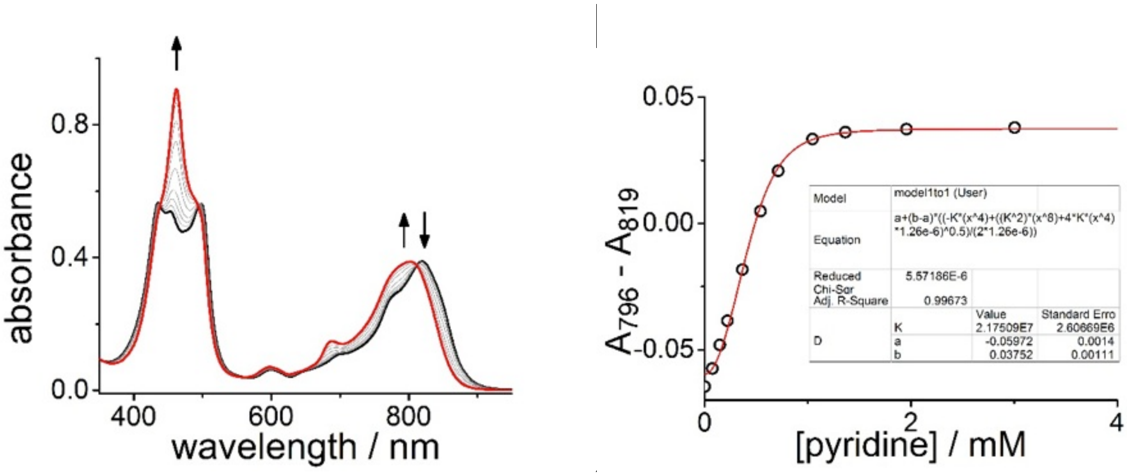

Figure S12: UV-vis-NIR titration of pyridine and $P5_{2H}$·T5. R^2 = 0.9967. (Run 2, $CHCl_3$, 298 K, [$P5_{2H}$·T5] = 1.26 μM, K = 2.18 × 10^7 M^{-3}).

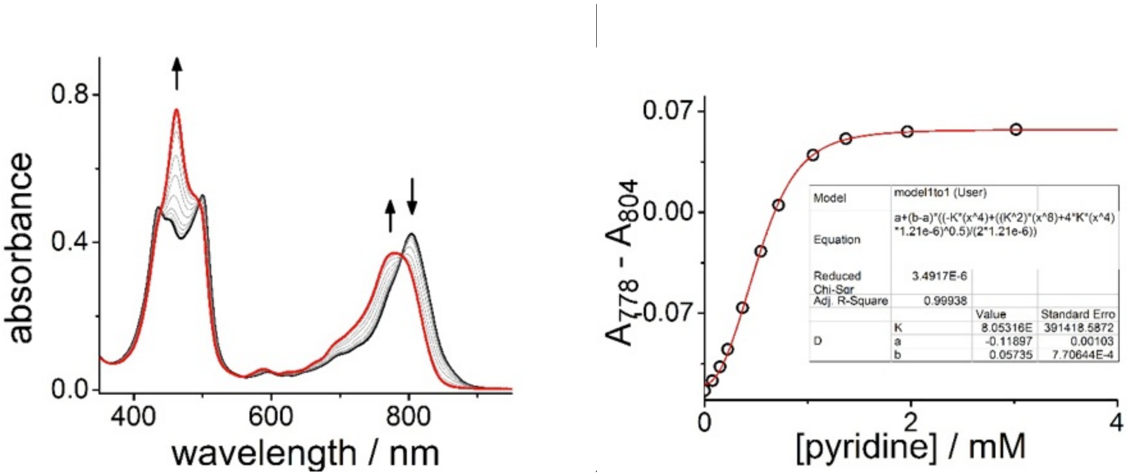

Figure S13: UV-vis-NIR titration of pyridine and $P5_{Cu}$·T4. R^2 = 0.9994. (Run 1, $CHCl_3$, 298 K, [$P5_{Cu}$·T4] = 1.21 μM, K = 8.05 × 10^6 M^{-3}).

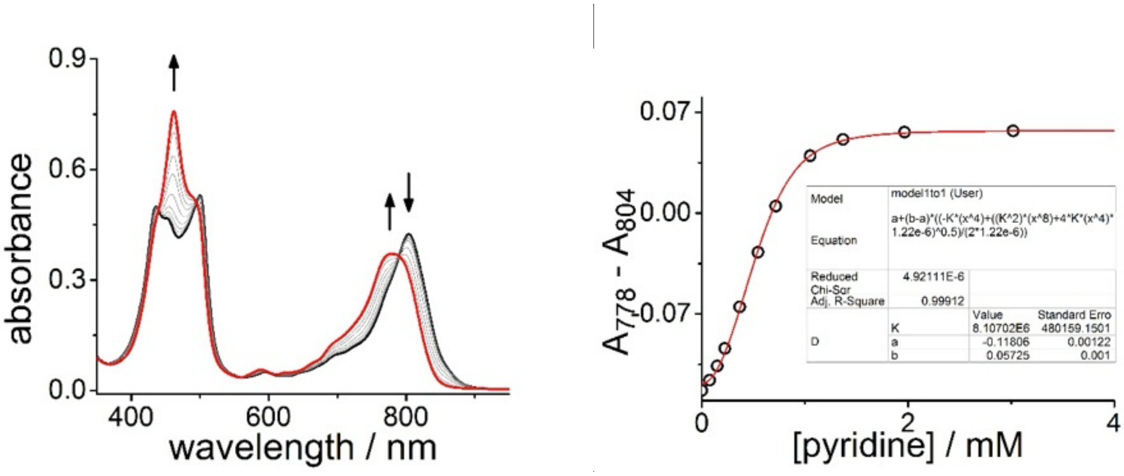

Figure S14: UV-vis-NIR titration of pyridine and $P5_{Cu}$·T4. R^2 = 0.9991. (Run 2, $CHCl_3$, 298 K, [$P5_{Cu}$·T4] = 1.22 μM, K = 8.11 × 10^6 M^{-3}).

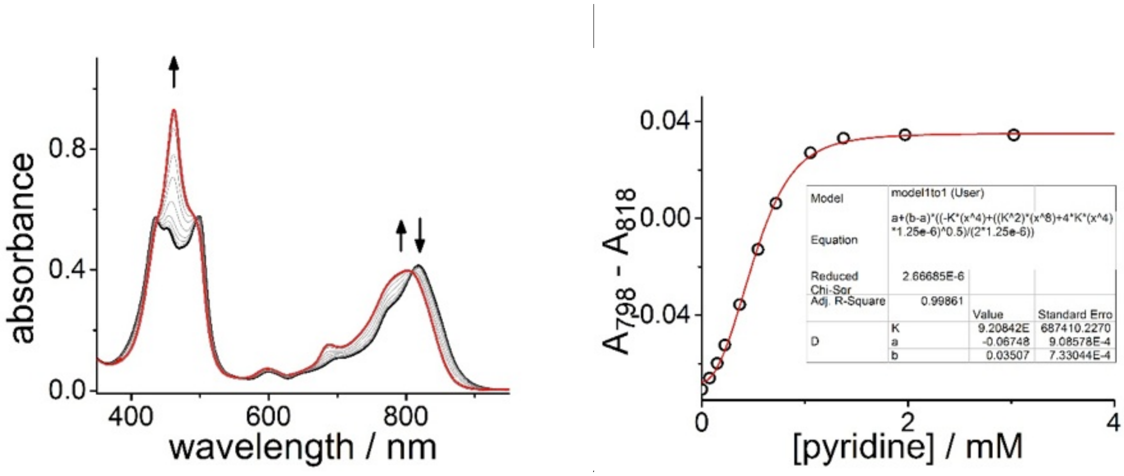

Figure S15: UV-vis-NIR titration of pyridine and $P5_{2H}$·T4. R^2 = 0.9986. (Run 1, $CHCl_3$, 298 K, [$P5_{2H}$·T4] = 1.25 μM, K = 9.21 × 10^6 M^{-3}).

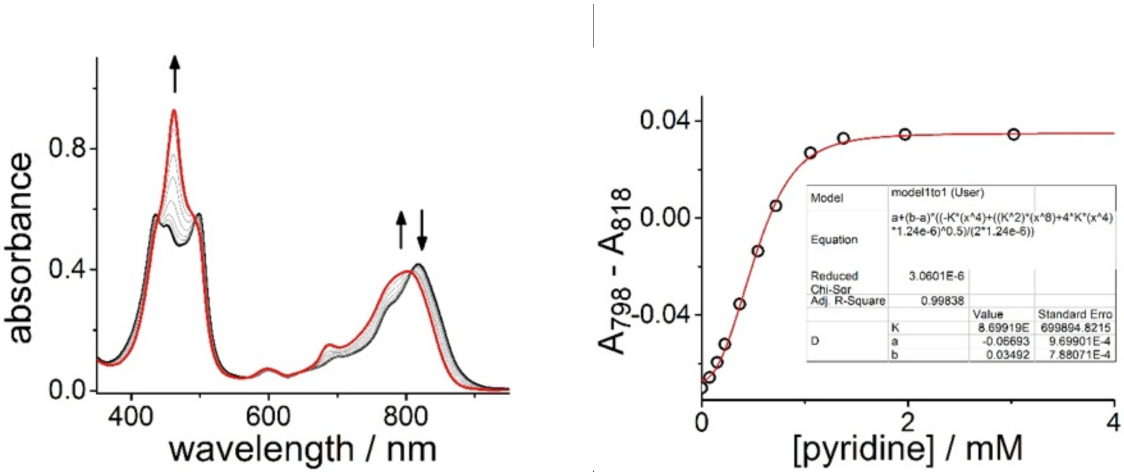

Figure S16: UV-vis-NIR titration of pyridine and $P5_{2H}$·T4. R^2 = 0.9984. (Run 2, $CHCl_3$, 298 K, [$P5_{2H}$·T4] = 1.24 μM, K = 8.70 × 10^6 M^{-3}).

Section 1.3. Denaturation of $P5_{Zn}$·T5 with Pyridine

Denaturation titrations were performed on the complex **$P5_{Zn}$·T5** with pyridine under the same conditions as described in Section 1.2.

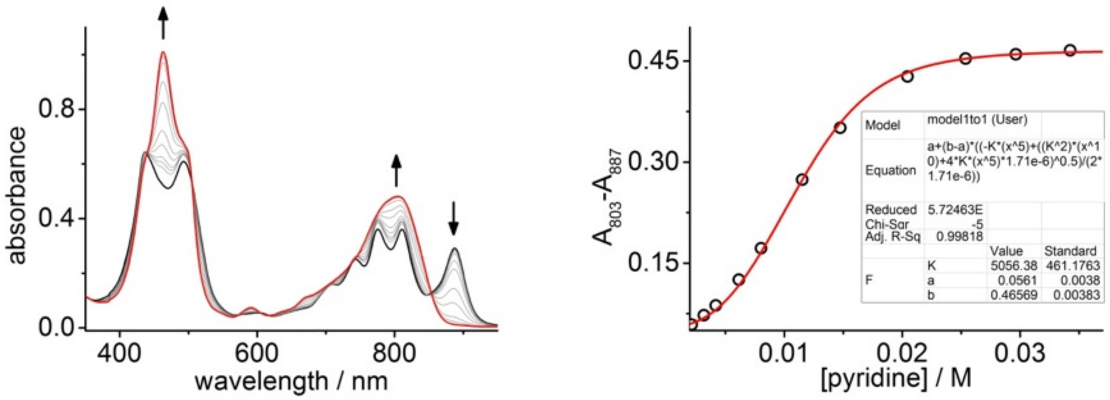

Figure S17: UV-vis-NIR titration of pyridine and $P5_{Zn}$·T5. R^2 = 0.9982. (Run 1, $CHCl_3$, 298 K, [$P5_{Zn}$·T5] = 1.71 μM, K = 5.06 × 10^3 M^{-4}).

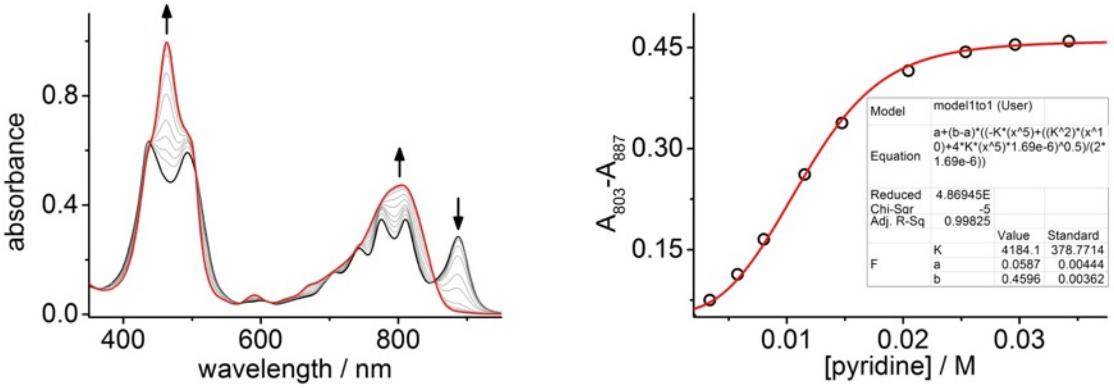

Figure S18: UV-vis-NIR titration of pyridine and $P5_{Zn}$·T5. R^2 = 0.9983. (Run 2, $CHCl_3$, 298 K, [$P5_{Zn}$·T5] = 1.69 μM, K = 4.18 × 10^3 M^{-4}).

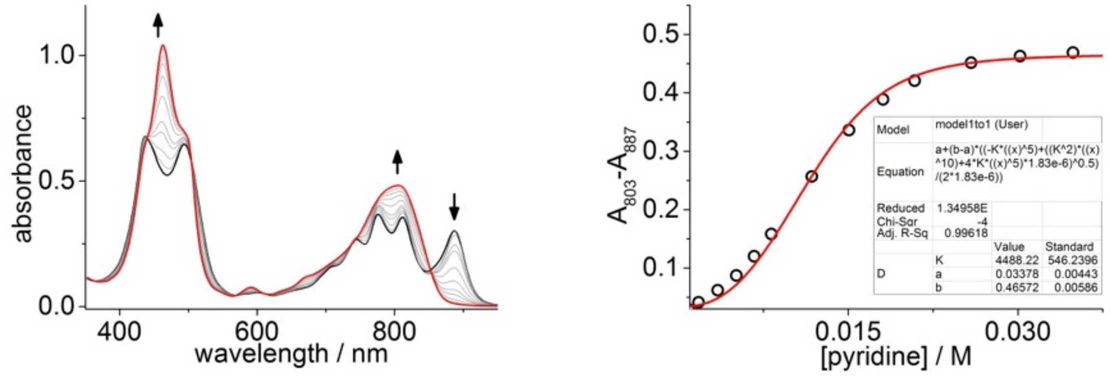

Figure S19: UV-vis-NIR titration of pyridine and $P5_{Zn}$·T5. R^2 = 0.9962. (Run 3, $CHCl_3$, 298 K, [$P5_{Zn}$·T5] = 1.83 μM, K = 4.49 × 10^3 M^{-4}).

The denaturation constant, 4.58 ± 0.48 × 10^3 M^{-4}, was translated into a statistically corrected association constant of 1.1 ± 0.4 × 10^{12} M^{-1}. To elucidate the effective molarity of the central porphyrin in the chain, we

look at a single mutation in which we compare the stability of **P5$_{Zn}$·T5** (1.1 ± 0.4 × 10^{12} M^{-1}) to the stability of **P5$_{2H}$·T5** (1.3 ± 0.2 × 10^{5} M^{-1}) according to the thermodynamic cycle below.

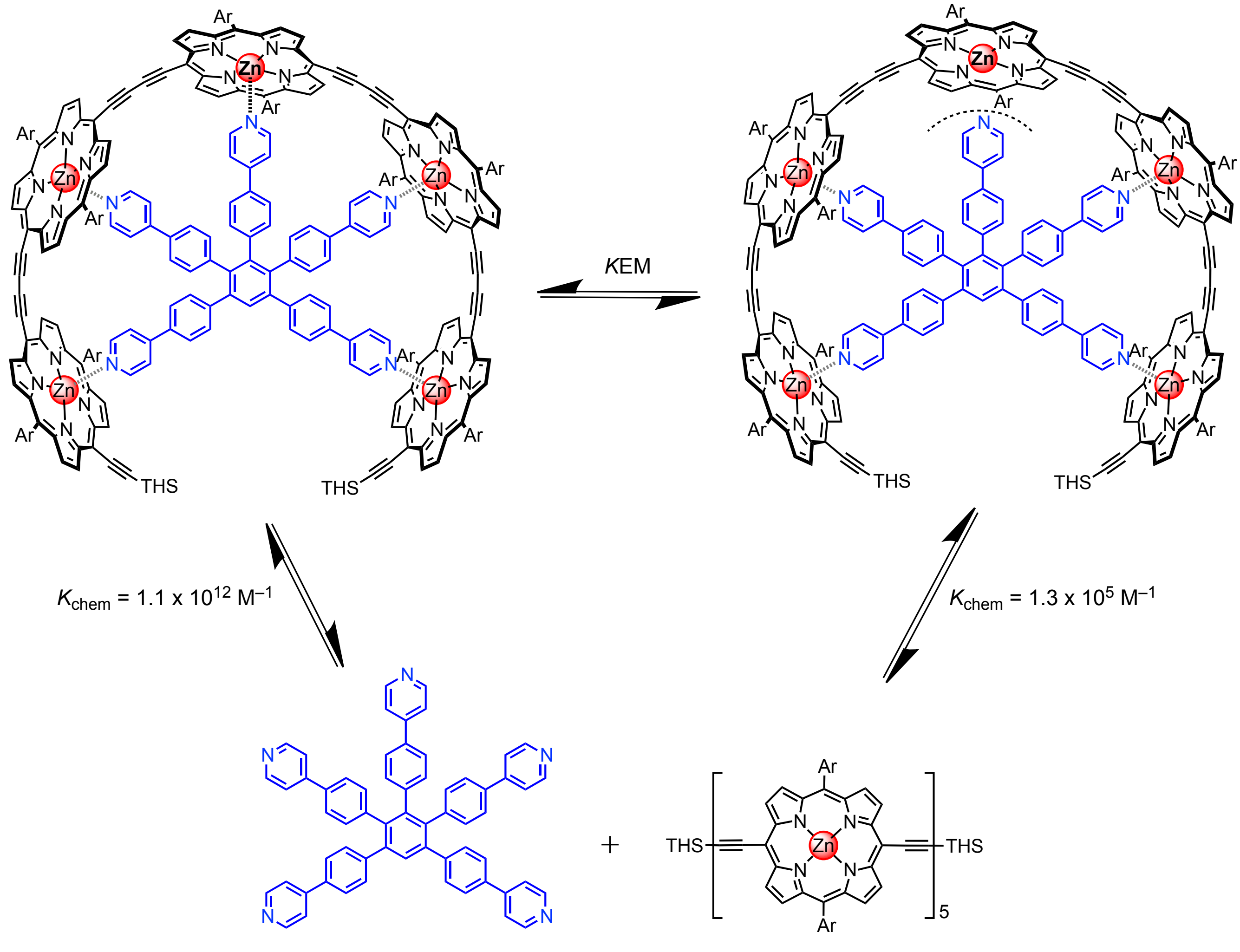

Figure S20: Complexes used to determine the interaction between the zinc center in the central porphyrin and the pyridine leg of the template T5 in $CHCl_3$ at 298 K. (Ar = 3,5-di-*tert*-butylphenyl) in order to determine the effective molarity of the central porphyrin.

The thermodynamic cycle shows the relationship between the individual species and allows us to determine the EM:

$$K_{\mathrm{Ph}}\mathrm{EM} = \frac{K_{\mathrm{f}}(\boldsymbol{P5_{Zn}} \cdot \boldsymbol{T5})}{K_{\mathrm{f}}(\boldsymbol{P5_{2H}} \cdot \boldsymbol{T5})}$$

$$EM = \frac{(1.1\times10^{12})}{(2.1\times10^{3})\times(1.3\times10^{5})} = 4.0\times10^{3}\ \mathrm{M}$$

where K_{Ph} is the equilibrium constant of the porphyrin monomer **P1$_{Zn}$** binding to 4-phenylpyridine (see Section 1.1). We determined the effective molarity of the central porphyrin unit as 4 ± 1 × 10^{3} M.

Section S2. Calculation of Statistical Factors

To understand the stability constants of different complexes, it is useful to factor out statistical contributions. Thus, a measured equilibrium constant K_{eq} can be factorized into its statistical component K_σ and its statistically corrected value K_{chem} according to the following equation:

$$wA + xB \rightleftharpoons yC + zD$$

$$K_{eq} = \frac{Q_{\mathbf{C}}^{y} Q_{\mathrm{D}}^{z}}{Q_{\mathbf{A}}^{w} Q_{\mathrm{C}}^{x}} = \frac{Q_{\mathbf{A}}^{w} Q_{\mathrm{B}}^{x}}{Q_{\mathbf{C}}^{y} Q_{\mathrm{D}}^{z}} \frac{Q'^{y}_{\mathbf{C}} Q'^{z}_{\mathrm{D}}}{Q'^{w}_{\mathbf{A}} Q'^{x}_{\mathrm{C}}} = K_\sigma K_{chem}$$

where for each species *i*, Q_i is the partition coefficient, Q'_i is the statistically corrected partition coefficient, and σ_i is the symmetry number.[1,3]

Values of K_σ were calculated using Benson's symmetry number method.[4,5] The symmetry number (σ) of each species is the product of its external symmetry number, σ_{ext}, (calculated from the point group of the molecule) and its internal symmetry number, σ_{int}, (calculated from the number of degenerate internal rotors). The values of σ for all the complexes involved are shown in Table S2. The external symmetry number is defined as the number of different but indistinguishable atomic arrangements that can be obtained by rotating a given molecule as a whole as a rigid object. The internal symmetry number is defined as the number of different but indistinguishable atomic arrangements that can be obtained by internal rotations around single bonds.

Table S2: Internal, external and total symmetry numbers for each component.*

component	point group	σ_{int}	σ_{ext}	σ
**pyridine**	C_{2v}	1	2	2
**4-phenylpyridine**	C_{2v}	1	2	2
**T5**	C_{2v}	1	2	2
**T4**	D_{2h}	1	4	4
**P1$_{Zn}$**	D_{2h}	1	4	4
**P5$_{2H}$**	D_{2h}	$2^4 = 16$	4	64
**P5$_{Cu}$**	D_{2h}	$2^4 = 16$	4	64
**P5$_{Zn}$**	D_{2h}	$2^4 = 16$	4	64
**P5$_{2H}$· T5**	C_{2v}	2	2	4
**P5$_{Cu}$· T5**	C_{2v}	1	2	2
**P5$_{Zn}$· T5**	C_{2v}	1	2	2
**P5$_{2H}$· T4**	C_{2v}	2	2	4
**P5$_{Cu}$· T4**	C_{2v}	2	2	4

[* Note that when counting internal rotations and calculating σ_{int}, we do not include rotors which are unaffected by the binding process, such as the *para*-phenylene links in **T5** because if a rotor is unaffected by the binding process it has no influence on K_σ.]

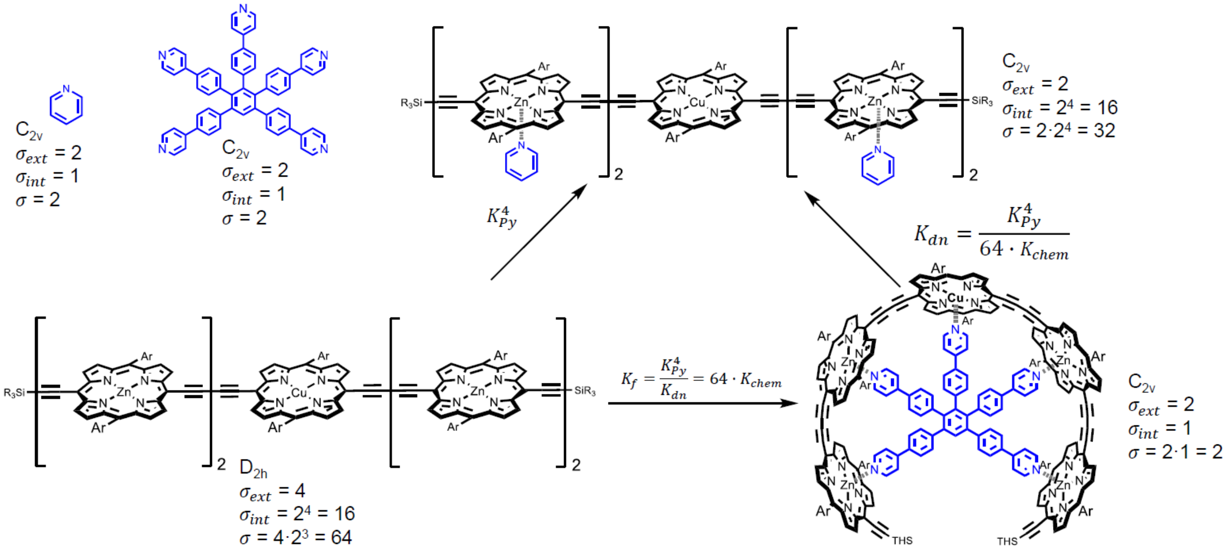

Figure S21: Statistical factors involved in the formation and denaturation (pyridine) of the $P5_{Cu}$·T5 complex. K_σ = 64.

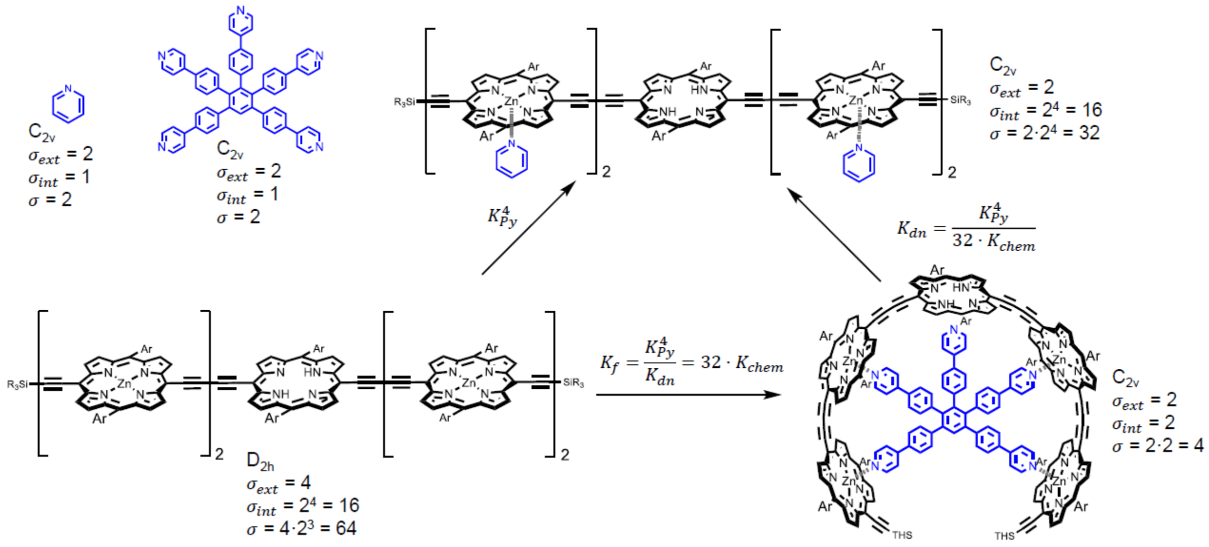

Figure S22: Statistical factors involved in the formation and denaturation (pyridine) of the $P5_{2H}$·T5 complex. K_σ = 32.

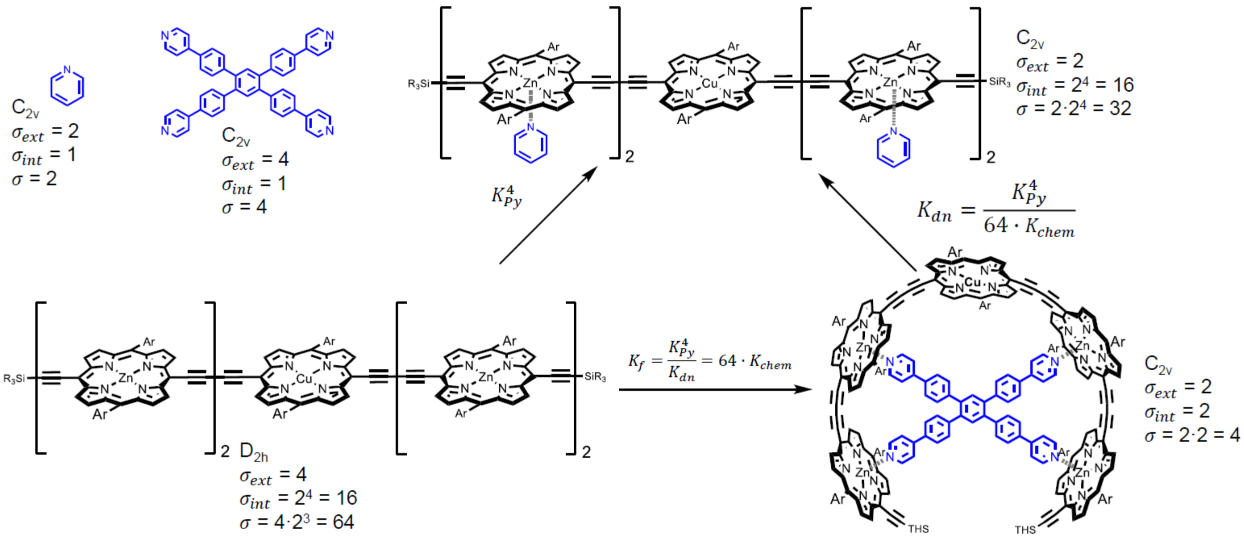

Figure S23: Statistical factors involved in the formation and denaturation (pyridine) of the P5$_{Cu}$·T4 complex. K_σ = 64.

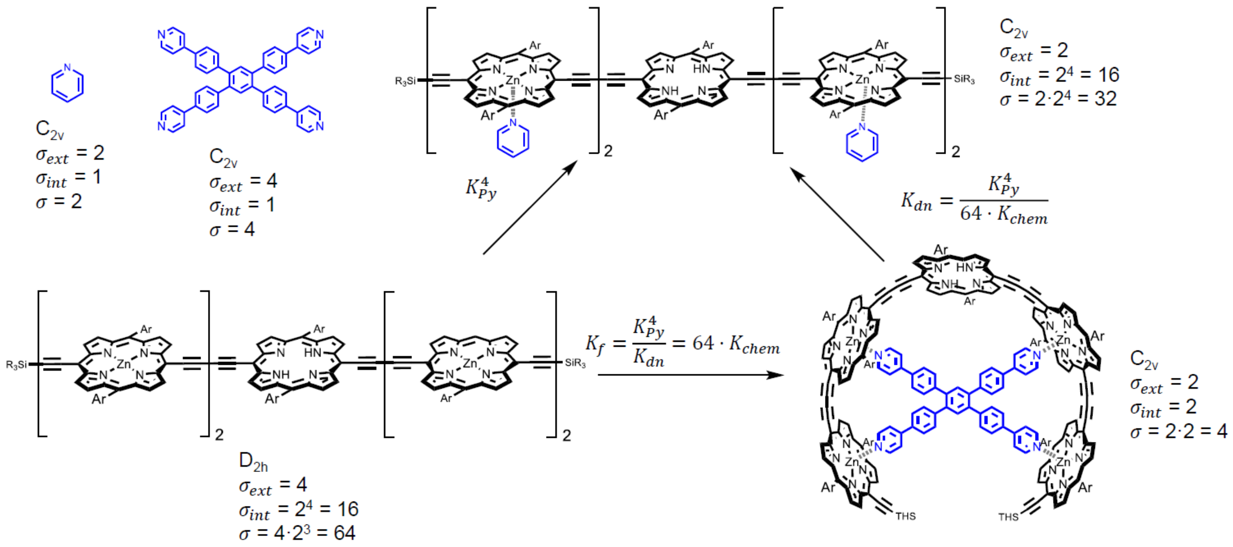

Figure S24: Statistical factors involved in the formation and denaturation (pyridine) of the P5$_{2H}$·T4 complex. K_σ = 64.

Section S3. ^{1}H NMR Spectra of Linear Pentamers

The Figure below shows the aromatic region of the proton NMR spectra corresponding to the linear pentamers containing either a central copper (**P5$_{Cu}$**) or a zinc porphyrin (**P5$_{Zn}$**). Due to the large similarity in the chemical environments of many of the signals, much overlap is observed. The overlap prevents us from being able to determine the T_1 and T_2 relaxation times but we can clearly see that the fine structure of the signals in **P5$_{Cu}$** which are spatially far removed from the paramagnetic copper center is retained (e.g. signals a and b) in **P5$_{Cu}$**.

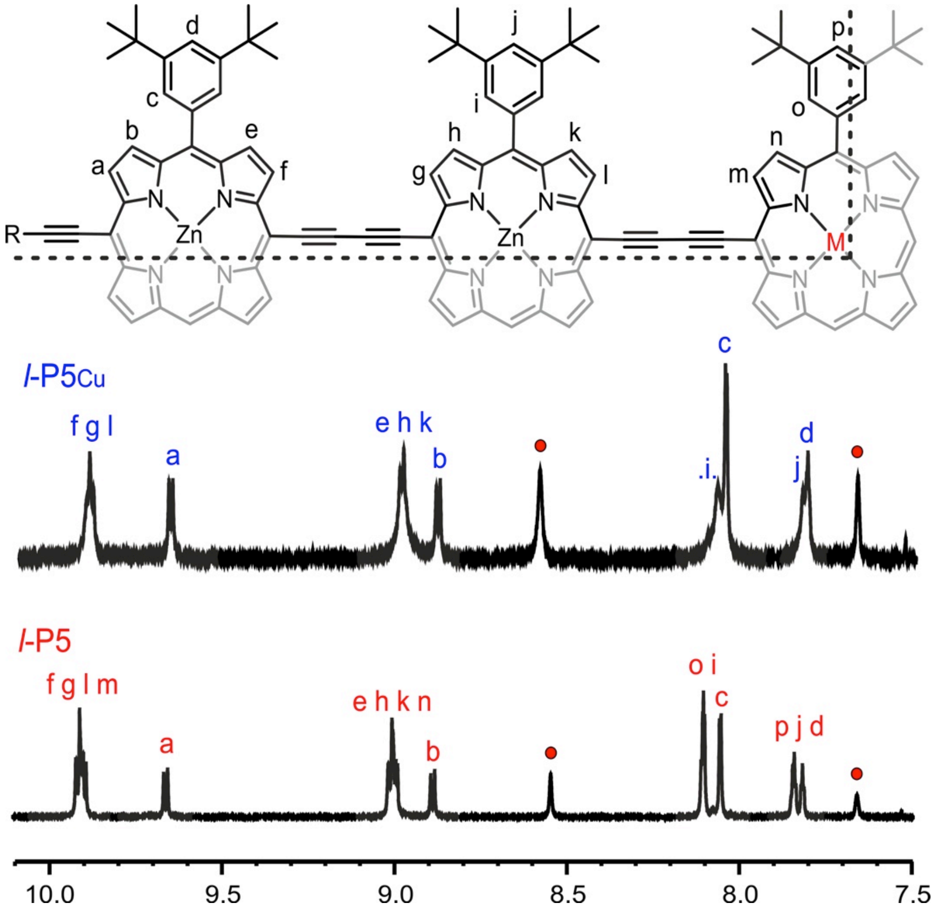

Figure S25: The ^{1}H NMR spectra ($CDCl_3$, 400 MHz, 298 K) of P5$_{Cu}$ and P5$_{Zn}$ and the general signal assignment for the oligomer. Only the aromatic part of the spectrum is shown. The signals assigned with the red dots correspond to *d*$_5$-pyridine.

Section S4. DFT Calculations

Constrained DFT geometry optimizations were carried out for several fixed separation distances between pyridine and the central metal of the porphyrin unit to explore the binding strength of pyridine to **P1$_{Cu}$** in comparison to **P1$_{Zn}$**. The optimizations were carried out in Turbomole V6.1[6] under C_2 symmetry using DFT/B3LYP in combination with the TZVP basis set,[7] RI-approximation[8] and an empirical dispersion correction to the energies.[9,10]

The SCF energy differences with respect to the minimum structure were then plotted as a function of the metal-pyridine separation distance and are shown in Figure S26. From these calculations, the following conclusions can be drawn: The metal···pyridine equilibrium separation distance (d_1) is calculated to be 2.18 Å for **P1$_{Zn}$**, whereas it is found to be 2.35 Å for **P1$_{Cu}$**. The potential energy gain for **P1$_{Cu}$** is considerably less than for **P1$_{Zn}$**, indicating that the binding of pyridine is weaker in the case of copper as the central metal. However,

since we are only comparing SCF energies, no relative numbers shall be given here. Without dispersion correction equilibrium M···N_{Py} separation distances of 2.25 Å and 2.59 Å are obtained for **P1$_{Zn}$** and **P1$_{Cu}$**, respectively.

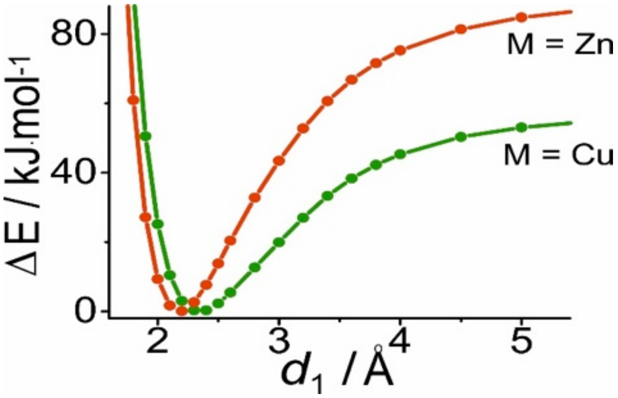

Figure S26: Total SCF energy differences with respect to the minimum structure as a function of the metal···pyridine separation distance for P1$_{Cu}$ (green line) and P1$_{Zn}$ (red line) obtained from a constraint geometry optimization in Turbomole using DFT/B3LYP in combination with the TZVP basis set and including dispersion correction for the energies.

Regarding the geometry of **P1$_{Zn}$**···Py, experimental data are available from X-ray crystallography in the CSD database. A statistical analysis of all available experimental data was found to yield a value of 2.16 ± 0.03 Å as the mean zinc-pyridine separation distance,[11] which is in very good agreement with the presented DFT results. A similar analysis based on X-ray data for the distance between the zinc atom and the porphyrin plane results in a mean value of 0.24 ± 0.06 Å.[11] This result compares favourably with the distance of 0.28 Å obtained for **P1$_{Zn}$** from the DFT calculations in this work. In the equilibrium geometry, the zinc atom is thus markedly pulled out of the porphyrin plane as is also shown in graphical form in Figure S27. For **P1$_{Cu}$**···Py a distance of 0.12 Å is found by DFT, indicating that the copper atom roughly remains in the centre of the porphyrin plane even when a pyridine ligand is bound.

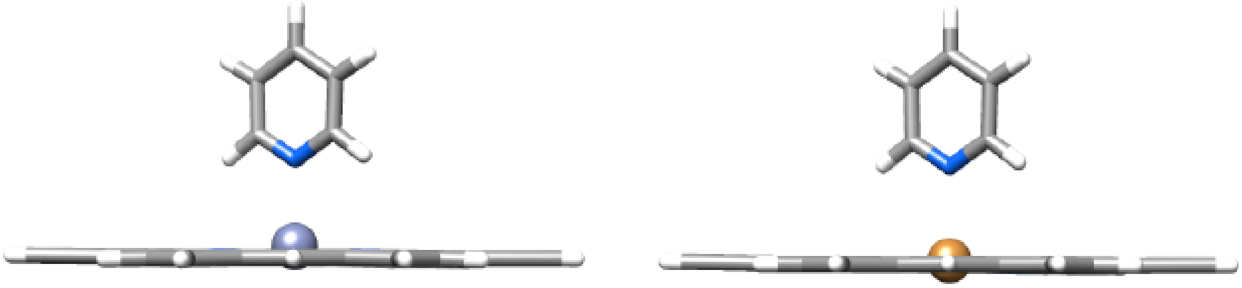

Figure S27: Side-view of the optimized minimum geometries of P1$_{Zn}$···Py (left) and P1$_{Cu}$···Py (right). The zinc center is pulled further out of the plane of the porphyrin upon binding pyridine and a smaller seperation distance is found.

Figure S27 shows a comparison of the optimized structures for **P1$_{Zn}$**···Py and **P1$_{Cu}$**···Py obtained from DFT to illustrate the different equilibrium metal···pyridine separation distances and the different locations of the metal atom with respect to the porphyrin plane. The calculated equilibrium metal···pyridine separation distance is increased in **P1$_{Cu}$**···Py (2.35 Å) as compared to **P1$_{Zn}$**···Py (2.18 Å). Upon binding of a pyridine ligand, the zinc atom is markedly pulled out of the porphyrin plane, whereas the position of the copper atom with respect to the porphyrin plane is much less affected.

Section S5. T_1 and T_2 Relaxation Time Constant Measurements

Section S5.1. T_1 Measurements on $P3_{2H}$

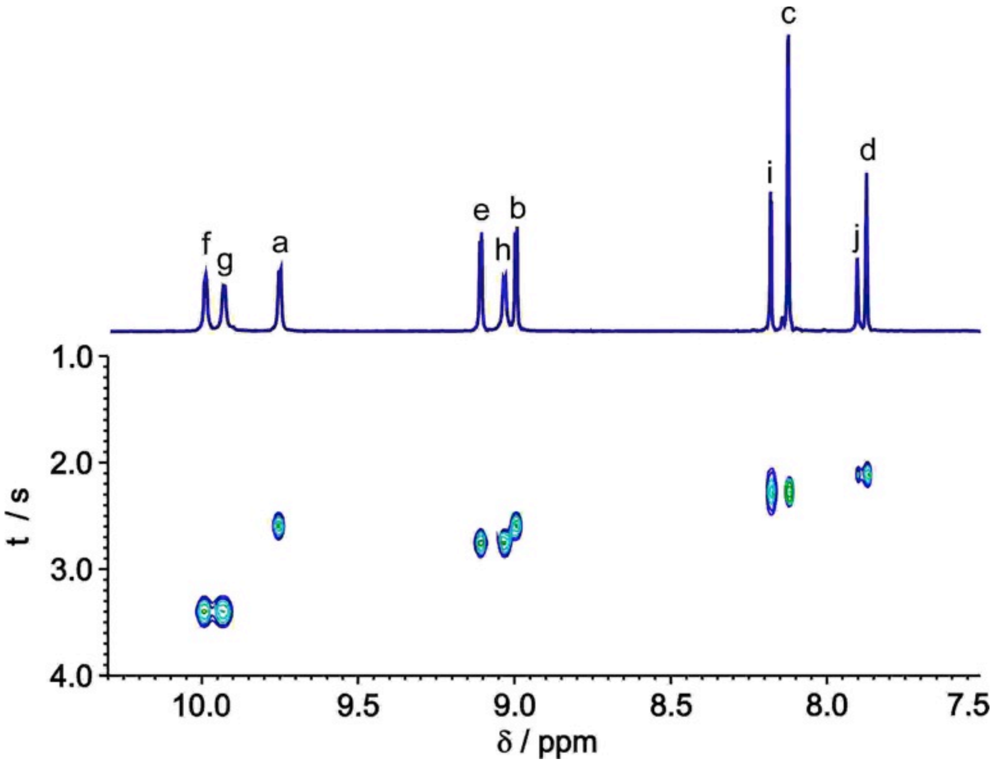

Figure S28: The ^{1}H NMR spectrum of $P3_{2H}$ ($CDCl_3$, 700 MHz, 298 K). Only the aromatic part of the spectrum is shown. The graph below shows the cross signals between chemical shift of the proton signals with respect to the corresponding T_1 relaxation time constants.

Table S3. Summary of the data describing the chemical shift of the aromatic proton signal of $P3_{2H}$ with the accompanying T_1 relaxation time constants.

Peak name	δ (ppm)	T_1 (s)	error (s)
**f**	9.987	3.33	0.098
**g**	9.928	3.32	0.10
**a**	9.749	2.53	0.048
**e**	9.106	2.68	0.024
**h**	9.031	2.78	0.041
**b**	8.992	2.51	0.026
**i**	8.178	2.24	0.16
**c**	8.121	2.22	0.015
**j**	7.901	2.15	0.020
**d**	7.872	2.08	0.020

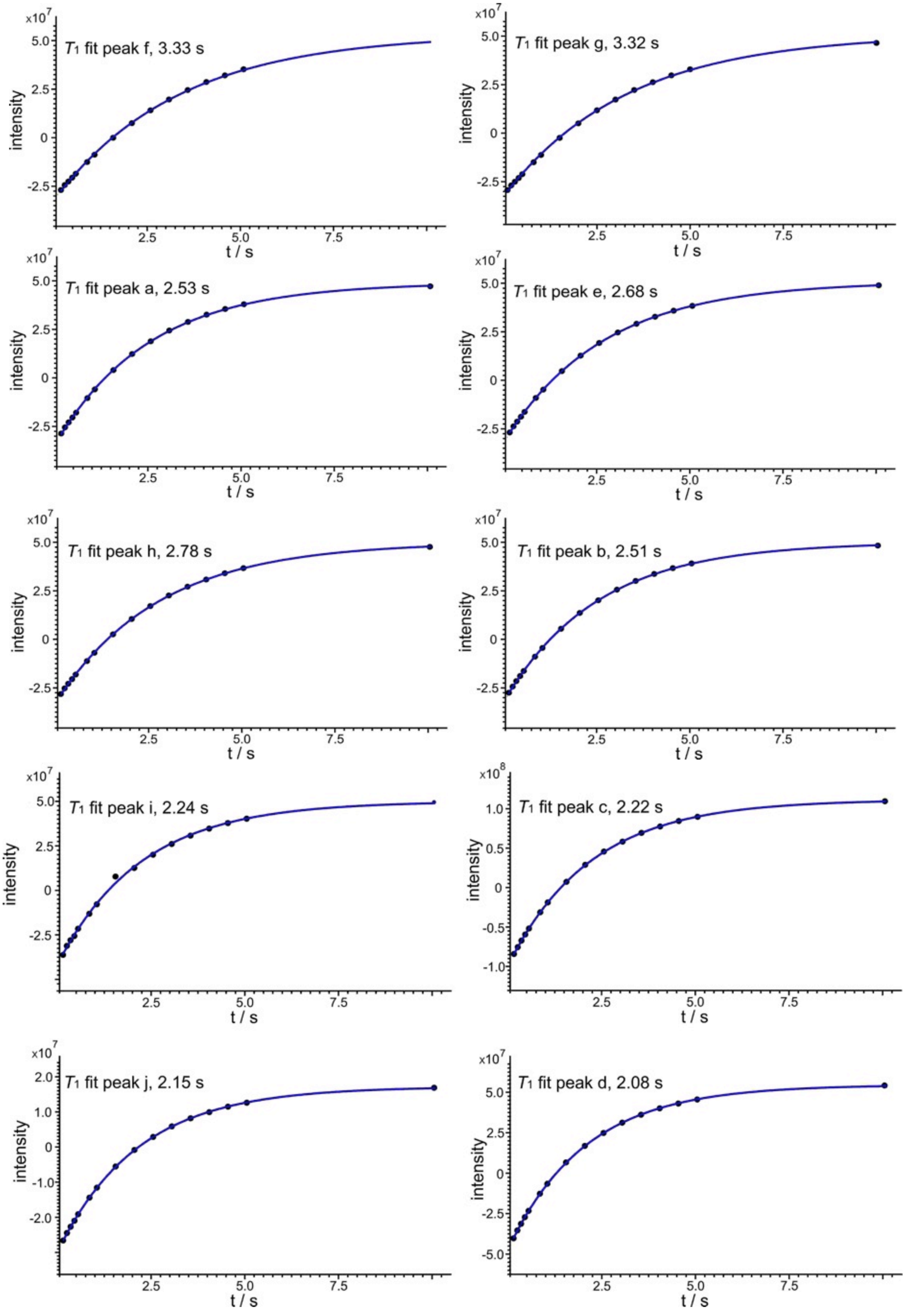

Figure S29: Inversion recovery curves and exponential fits for the T_1's of P3$_{2H}$ peaks a–j.

Section S5.2. T_2 Measurements on $P3_{2H}$

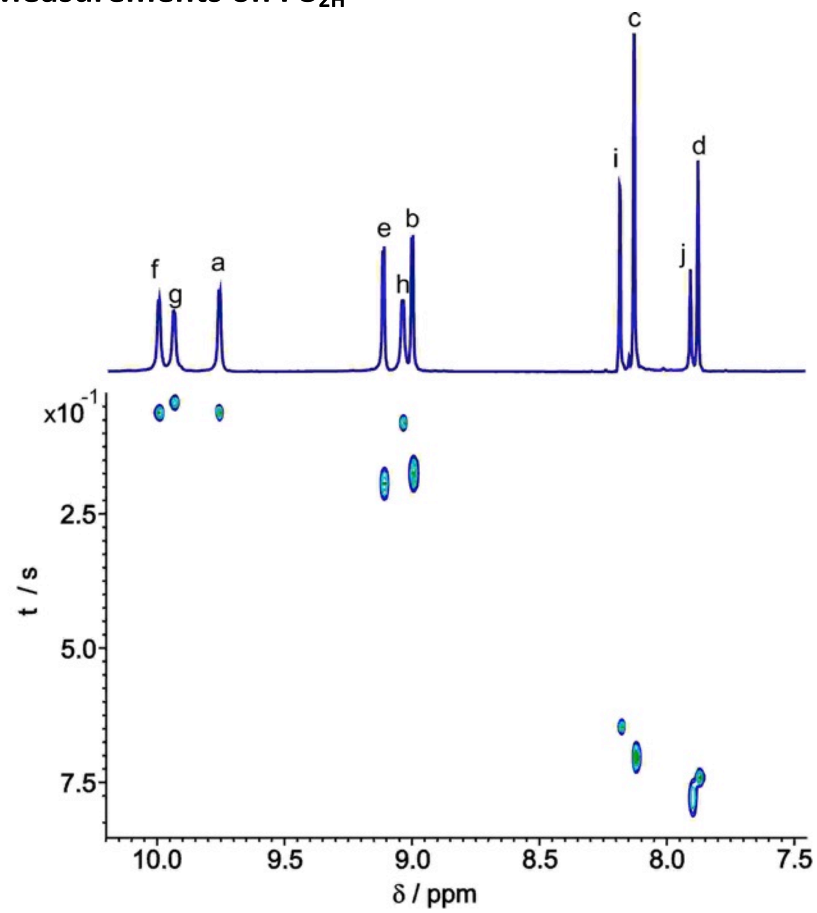

Figure S30: The ^{1}H NMR spectrum of $P3_{2H}$ ($CDCl_3$, 700 MHz, 298 K). Only the aromatic part of the spectrum is shown. The graph below shows the cross signals between chemical shift of the proton signals with respect to the corresponding T_2 relaxation time constants.

Table S4. Summary of the data describing the chemical shift of the aromatic proton signal of $P3_{2H}$ with the accompanying T_2 relaxation time constants.

Peak name	δ (ppm)	T_2 (s)	error (s)
**f**	9.987	0.0564	0.0019
**g**	9.927	0.0520	0.0017
**a**	9.750	0.0599	0.0058
**e**	9.106	0.190	0.0209
**h**	9.031	0.0729	0.0036
**b**	8.991	0.177	0.0281
**i**	8.178	0.639	0.0092
**c**	8.122	0.698	0.0165
**j**	7.901	0.775	0.0321
**d**	7.872	0.748	0.0135

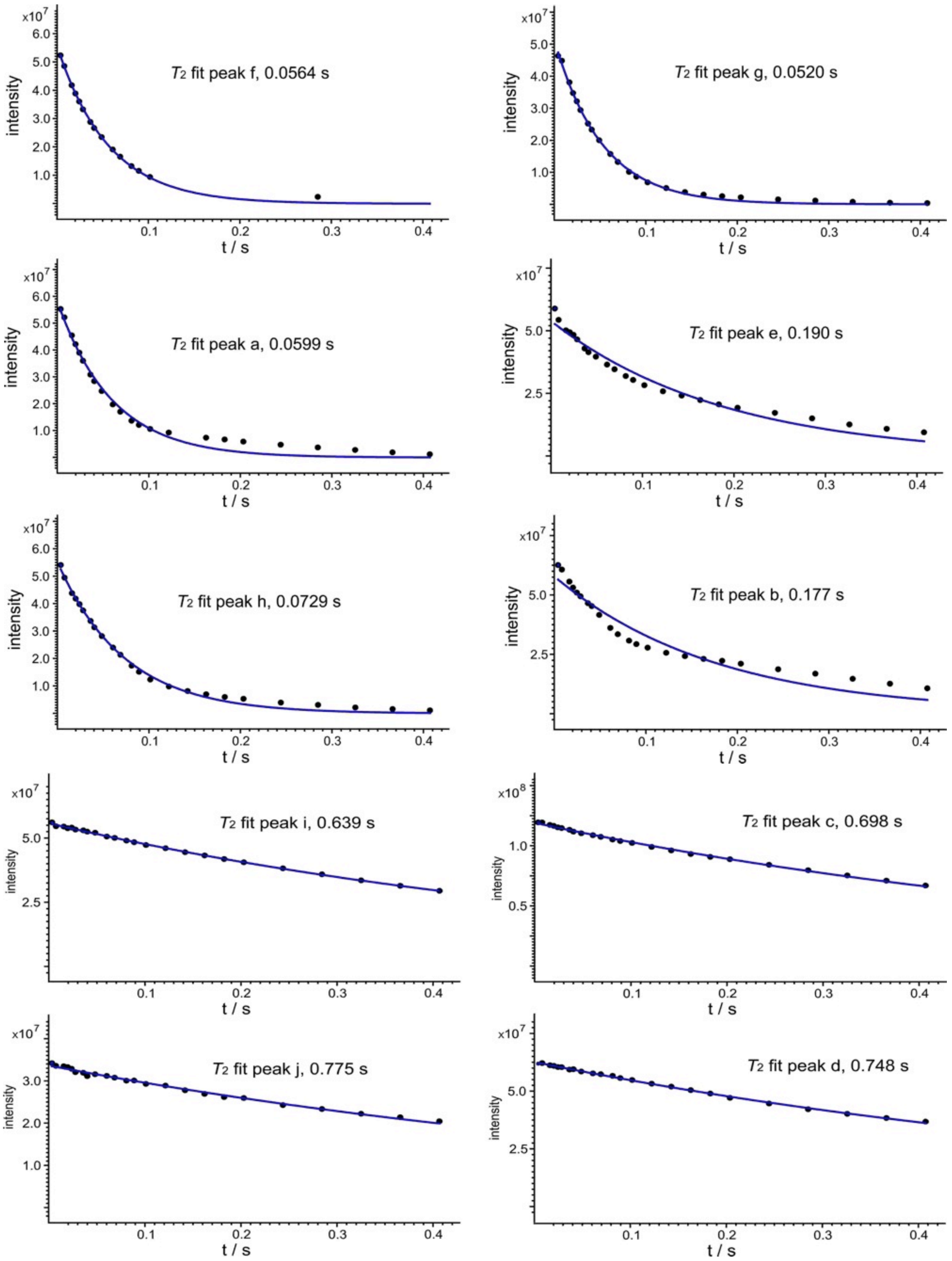

Figure S31: CPMG relaxation curves and exponential fits for the T_2's of P3$_{2H}$ peaks a–j.

Section S5.3. T_1 Measurements on $P3_{Cu}$

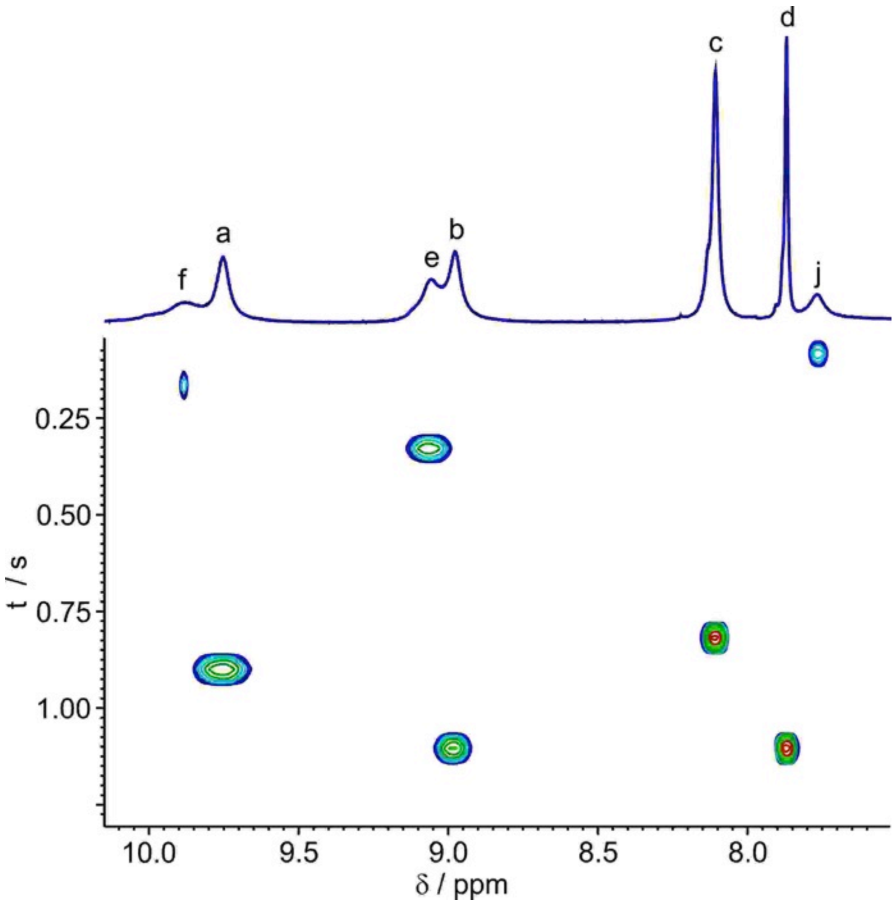

Figure S32: The ^{1}H NMR spectrum of $P3_{Cu}$ ($CDCl_3$, 700 MHz, 298 K). Only the aromatic part of the spectrum is shown. The graph below shows the cross signals between chemical shift of the proton signals with respect to the corresponding T_1 relaxation time constants.

Table S5. Summary of the data describing the chemical shift of the aromatic proton signal of $P3_{Cu}$ with the accompanying T_1 relaxation time constant.

Peak name	δ (ppm)	T_1 (s)	error (s)
**f**	9.878	0.146	0.033
**a**	9.753	0.880	0.024
**e**	9.063	0.346	0.019
**b**	8.983	1.12	0.022
**c**	8.110	0.813	0.004
**d**	7.871	1.10	0.003
**j**	7.767	0.099	0.020

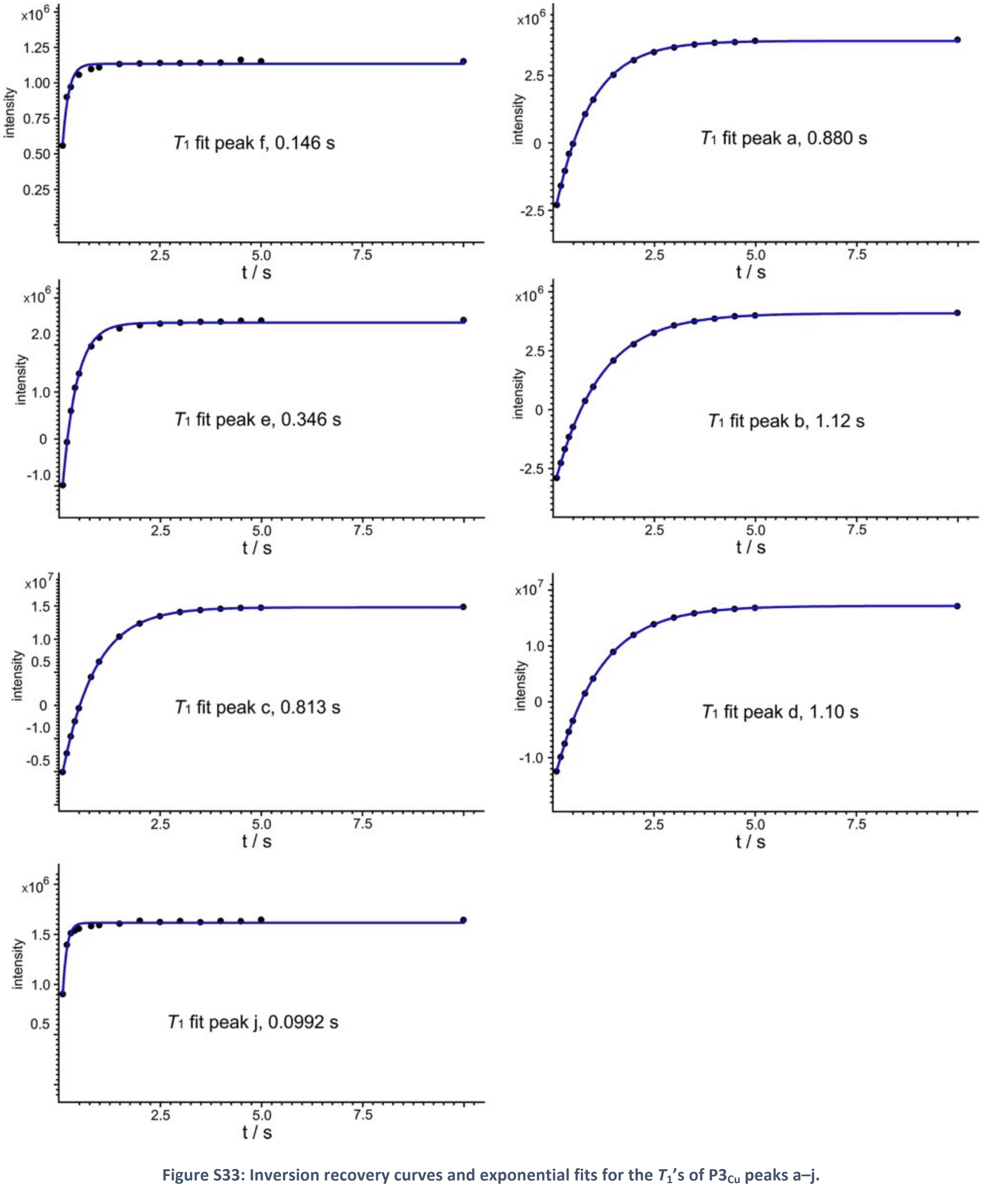

Figure S33: Inversion recovery curves and exponential fits for the T_1's of P3$_{Cu}$ peaks a–j.

Section S5.4. T_2 Measurements on $P3_{Cu}$

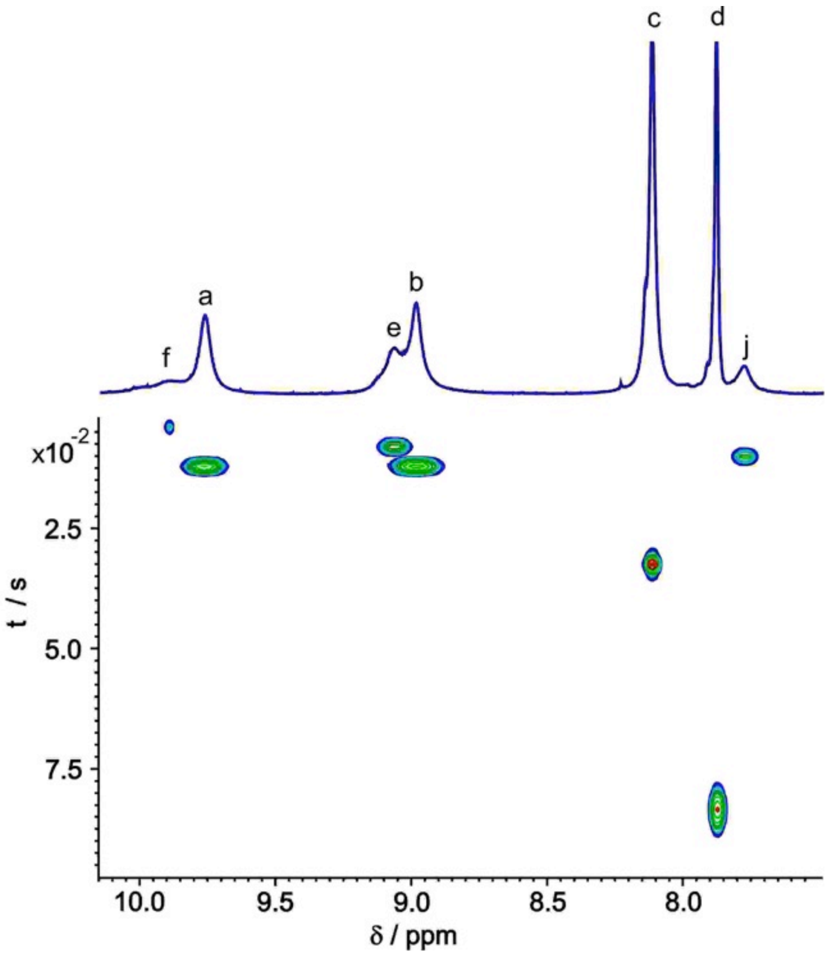

Figure S34: The ^{1}H NMR spectrum of $P3_{Cu}$ ($CDCl_3$, 700 MHz, 298 K). Only the aromatic part of the spectrum is shown. The graph below shows the cross signals between chemical shift of the proton signals with respect to the corresponding T_2 relaxation time constants.

Table S6. Summary of the data describing the chemical shift of the aromatic proton signal of $P3_{Cu}$ with the accompanying T_2 relaxation time constants.

Peak name	δ (ppm)	T_2 (s)	error (s)
**f**	9.887	0.00474	0.00018
**a**	9.756	0.0116	0.0003
**e**	9.060	0.00867	0.00040
**b**	8.980	0.0128	0.0004
**c**	8.114	0.0318	0.0012
**d**	7.875	0.0831	0.0028
**j**	7.774	0.0106	0.0009

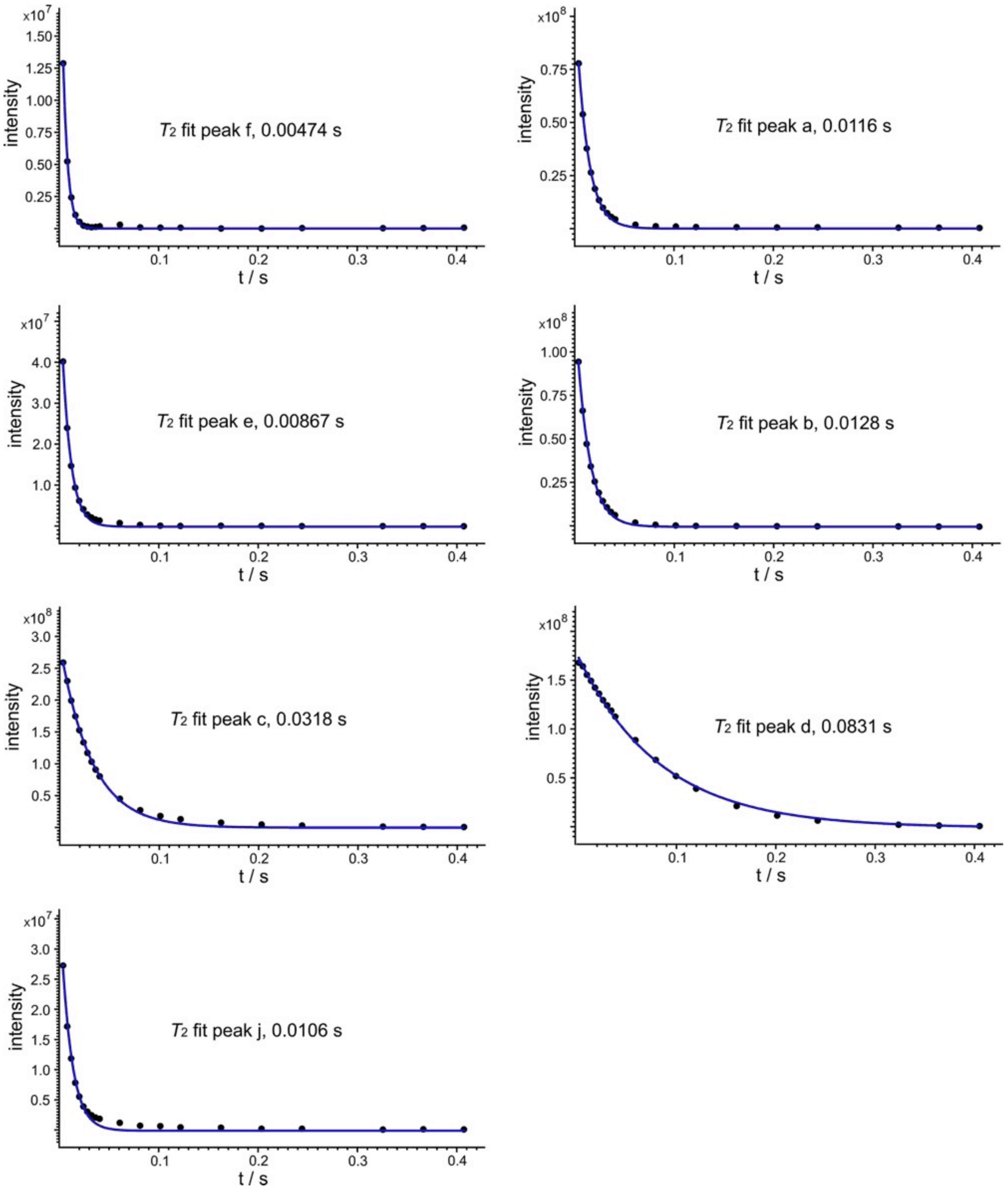

Figure S35: CPMG relaxation curves and exponential fits for the T_2's of $P3_{Cu}$ peaks a–j.

Section S5.5. R_1 and R_2 decay rate versus distance plots

The dipolar relaxation rate is expected to depend on the inverse of the 6th power of the distance between a nucleus and an electron. We have taken the experimental T_1 and T_2 relaxation time constants and plotted the change in the corresponding rates ($R_1 = 1/T_1$ and $R_2 = 1/T_2$) between $\mathbf{P3_{Cu}}$ and $\mathbf{P3_{2H}}$ to the distance between the copper center and the corresponding proton. The distance is estimated from crystal structures of similar oligomers. The experimental data were fitted to equation (S1):

$$y = \frac{A}{r^6} \tag{S1}$$

where y is the change in relaxation rate ($\frac{1}{T_1(\mathbf{P3_{Cu}})} - \frac{1}{T_1(\mathbf{P3_{2H}})}$ or $\frac{1}{T_2(\mathbf{P3_{Cu}})} - \frac{1}{T_2(\mathbf{P3_{2H}})}$), r is the distance between the proton and metal center and A is a free fitting parameter. The T_1 data fit with relatively good accuracy, confirming the expected distance dependence. The trend can also be observed for the T_2 data but the variation in the points in much larger and only an overall trend can be observed relating dipolar relaxation and distance between the two nuclei.

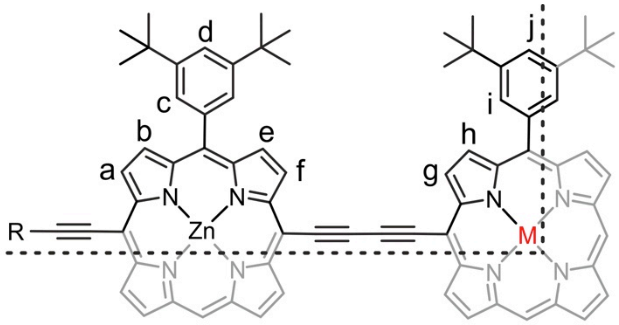

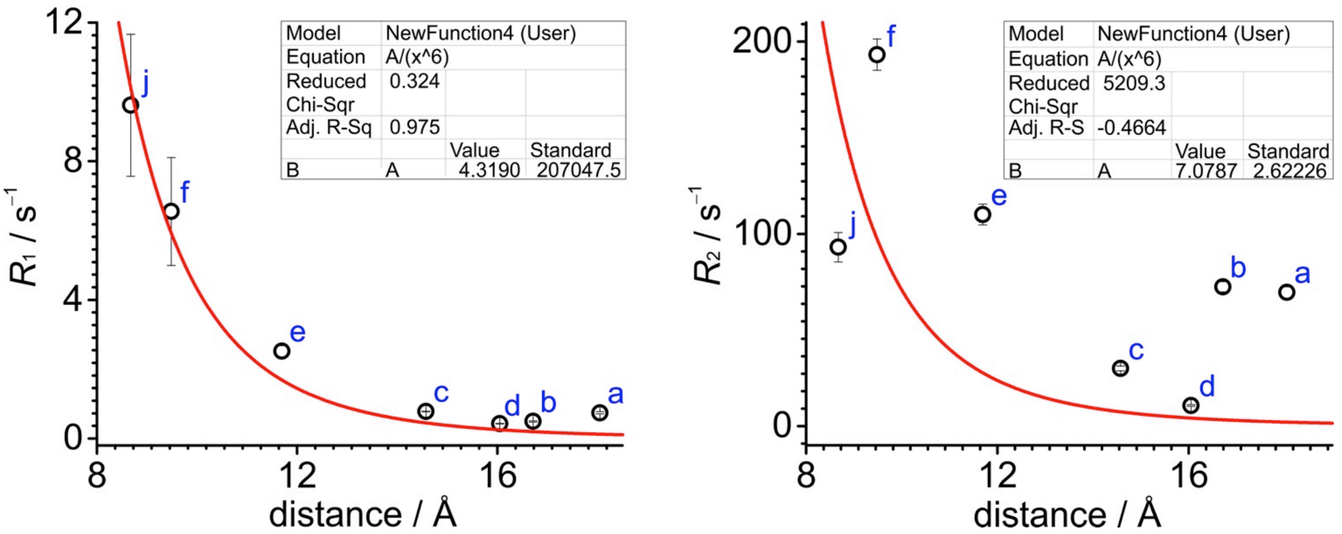

Figure S36: Changes in relaxation rates from comparing R_1 and R_2 values in $P3_{Cu}$ and $P3_{2H}$ for peaks a–j.

Section S6. Experimental Procedures

General Experimental

Dry toluene and THF were obtained by passing the solvents through columns of alumina, under nitrogen. Diisopropylamine (DIPA) was distilled from CaH_2 and kept over activated molecular sieves (3 Å, 8–12 mesh). Unless specified otherwise, all other solvents were used as commercially supplied. Flash chromatography was carried out on silica gel 60 under positive pressure. Analytical thin-layer chromatography was carried out on aluminum-backed silica gel 60 F254 plates. Visualization was achieved using UV light when necessary.

All UV-vis-NIR spectra were recorded in solution using a Perkin-Lambda 20 spectrometer (1 cm path length quartz cell). Chloroform (containing ca. 0.5% ethanol as stabilizer) was used for all titrations without any further purification.

Unless stated otherwise, $^{1}H/^{13}C$ NMR spectra were recorded at 298 K using Bruker AV400 (400/100 MHz), Bruker AV500 (500/125 MHz), Bruker AV600 (600/150 MHz), Bruker AV700 (700/175 MHz) instruments. ^{1}H, and ^{13}C NMR spectra are reported in ppm; coupling constants are given in Hertz, to the nearest 0.1 Hz. The solvent used were $CDCl_3$ or a mixture of $CDCl_3$ and pyridine-d_5 (99:1 by volume).

MALDI-TOF mass spectra were carried out using Waters MALDI Micro MX spectrometer.

Free-base 10,20-di-trihexyl(ethylnyl)silane-5,15-bis-(3,5-di-*tert*-butylphenyl)porphyrin ($\mathbf{P1_{2H}}$)

$\mathbf{P1_{Zn}}$ (955 mg, 0.7 mmol) was dissolved in $CHCl_3$ (250 mL). Trifluoroacetic acid (2.7 mL) was mixed with $CHCl_3$ (24 mL) to give a 10% solution). Both solutions were degassed. The TFA solution was added dropwise to the porphyrin solution and the reaction mixture was stirred at room temperature under nitrogen for 15 min. UV and TLC indicated the completion of the reaction and the mixture was passed immediately through a short plug of silica gel ($CHCl_3$). Column chromatography (50:1:1, 40-60 petroleum ether : ethyl acetate : pyridine) gave the title compound as a dark solid (622 mg, 68%).

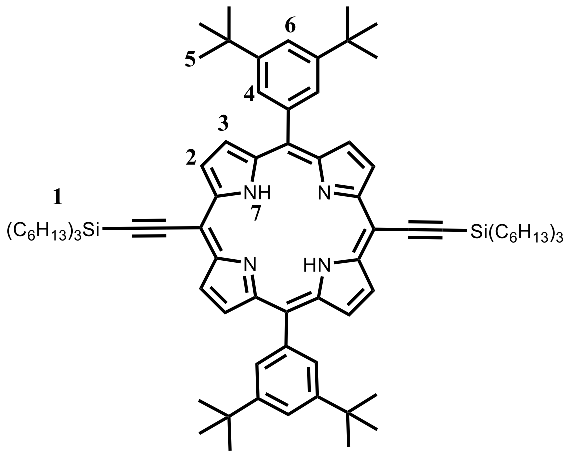

^{1}H NMR (400 MHz, $CDCl_3$, 298 K): δ_H (ppm) 9.62 (4H, d, *J* = 4.7 Hz, H2), 8.85 (4H, d, *J* = 4.7 Hz, H3), 8.04 (4H, bd, *J* = 1.5 Hz, H4), 7.82 (2H, bt, H6), 1.55 (36H, s, H5), 1.75–0.61 (78H, m, H1), –2.12 (2H, s, H7).
^{13}C NMR (125 MHz, $CDCl_3$, 298 K): δ_C (ppm) 149.0, 140.3, 129.7, 123.1, 121.3, 108.1, 101.0, 100.8, 35.0, 33.3, 31.7, 24.4, 22.7, 14.2, 13.8.
MALDI-TOF: *m*/*z* = 1299 ($C_{88}H_{130}N_4Si_2$, M^+ requires 1300).

Free-base 10,20-di-ethynyl-5,15-bis-(3,5-di-*tert*-butylphenyl)porphyrin ($P1''_{2H}$)

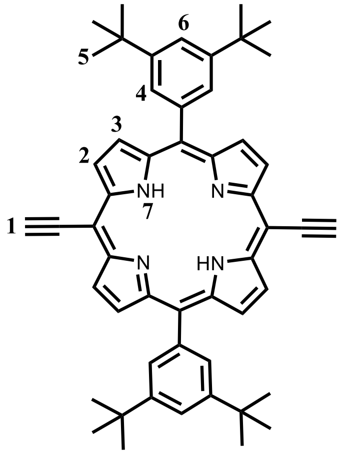

$P1_{2H}$ (100 mg, 0.077 mmol) was dissolved in CH_2Cl_2 (25 mL) under a flow of nitrogen. Tetra-*n*-butylammonium fluoride solution (1.0 M in THF, 1.31 mL, 1.31 mmol) was added dropwise to the reaction mixture and was stirred at room temperature for 15 min. The crude reaction mixture was immediately passed through a short plug of silica gel ($CHCl_3$). The product was recrystallized by layer addition (CH_2Cl_2/MeOH) to give the title compound as a purple powder (42.4 mg, 76%).

^{1}H NMR (400 MHz, $CDCl_3$, 298 K): δ_H (ppm) 9.66 (4H, d, *J* = 4.7 Hz, H2), 8.91 (4H, d, *J* = 4.7 Hz, H3), 8.05 (4H, bd, *J* = 1.6 Hz, H4), 7.78 (2H, bt, H6), 4.20 (2H, s, H1), 1.56 (36H, s, H5), –2.26 (2H, s, H7).
^{13}C NMR (100 MHz, $CDCl_3$, 298 K): δ_C (ppm) 149.2, 140.4, 130.0, 123.3, 121.5, 99.4, 85.7, 84.5, 35.2, 31.9, 29.9.
MALDI-TOF: *m/z* = 734 ($C_{52}H_{54}N_4$, M^+ requires 735).

Copper 5,15-(3,5-bis-*tert*-butyl-phenyl)-10,20-bis-trihexylsilanylethynyl-porphyrin ($P1_{Cu}$)

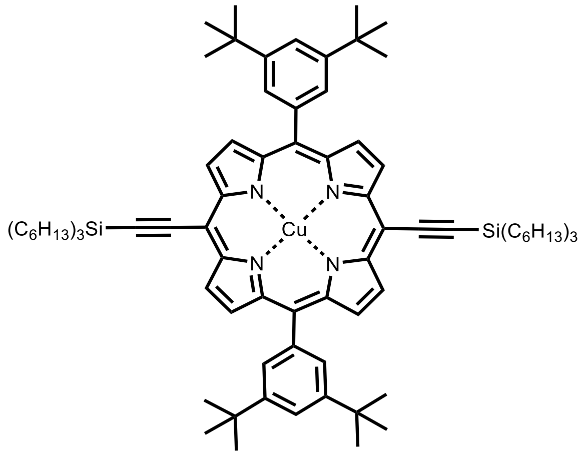

$P1_{2H}$ (47.6 mg, 0.036 mmol) was dissolved in $CHCl_3$ (15 mL). $Cu(OAc)_2{\cdot}H_2O$ (333 mg, 1.67 mmol) was added and the reaction was stirred at reflux for 2 h under nitrogen. The reaction mixture was allowed to cool to room temperature after which MeOH was added to the reaction mixture to precipitate the product. The product was filtered and washed with MeOH to give a green solid (38.6 mg, 77%).

^{1}H NMR (400 MHz, $CDCl_3$, 298 K): δ_H (ppm) broad.
MALDI-TOF: *m/z* = 1361 ($C_{88}H_{128}CuN_4Si_2$, M^+ requires 1362).
λ_{max} ($CHCl_3$) / nm log(ε): 610 (4.59), 567 (4.25), 434 (5.66).

P2'

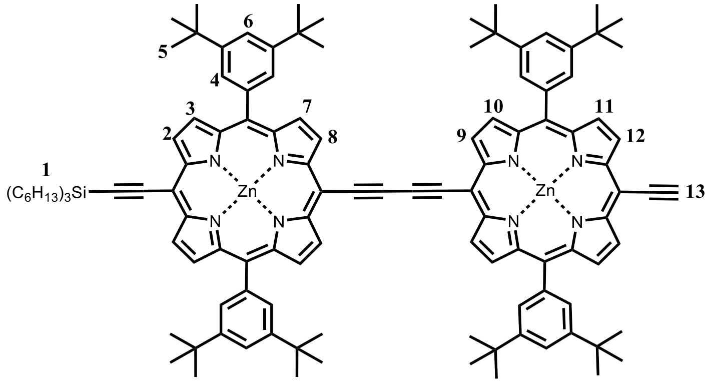

The dimer **P2** (0.54 g, 0.25 mmol) was dissolved in CH_2Cl_2 (60 mL), $CHCl_3$ (60 mL) and pyridine (1.2 mL). Tetra-*n*-butylammonium fluoride (0.375 mL, 1.0 M solution in THF, 0.375 mmol) was added to the stirred solution dropwise. The progress of the reaction was monitored by TLC until an optimal product mixture was reached. The mixture immediately was passed through a short plug of silica gel ($CHCl_3$ + 1% pyridine). Column chromatography (30:1:1, 40-60 petroleum ether : ethyl acetate : pyridine) gave:

P2' (179.0 mg):
^{1}H NMR (400 MHz, $CDCl_3$ + 1% pyridine-d_5, 298 K): δ_H (ppm) 9.93 (2H, d, *J* = 4.6 Hz, H8 or H9), 9.91 (2H, d, *J* = 4.6 Hz, H8 or H9), 9.69 (2H, d, H2 or H12), 9.68 (2H, d, H2 or H12), 9.02 (2H, d, H7 or H10), 9.01 (2H, d, H7 or H10), 8.94 (2H, d, *J* = 4.6 Hz, H3 or H11), 8.91 (2H, d, *J* = 4.5 Hz, H3 or H11), 8.07 (8H, m, H4), 7.83 (4H, m, H6), 4.20 (1H, s, H13), 1.79–0.92 (39H, m, H1), 1.58 (72H, s, H5).

P2'' (36.5 mg):
^{1}H NMR (400 MHz, $CDCl_3$, 298 K): δ_H (ppm) 9.93 (4H, d, *J* = 4.5 Hz, H8), 9.66 (4H, d, *J* = 4.5 Hz, H2), 8.98 (4H, d, *J* = 4.5 Hz, H7), 8.91 (4H, d, *J* = 4.5 Hz, H3), 8.04 (8H, m, H4), 7.80 (4H, m, H6), 4.16 (2H, s, H1), 1.55 (72H, s, H5).

P2 Recovered starting material (131.5 mg).

$P3_{2H}$[12]

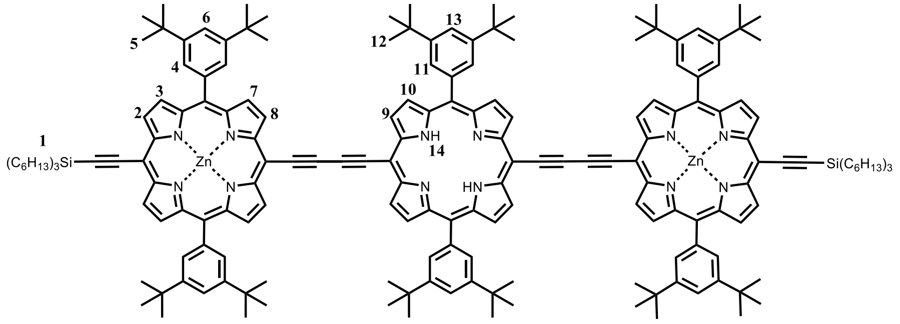

$P1''_{2H}$ (10.0 mg, 0.013 mmol), **P1'** (73.5 mg, 0.068 mmol), $Pd_2(dba)_3$ (4.36 mg, 0.0047 mmol), tri-2-furylphosphine (8.84 mg, 0.038 mmol) and 1,4-benzoquinone (13.2 mg, 0.122 mmol) were dissolved in a mixture of toluene : Et_3N (5 : 1) (16 mL). The reaction mixture was stirred at 60 ˚C overnight. The solvents were removed and the mixture was purified over a plug of silica gel ($CHCl_3$ + 1% pyridine). A small SEC column ($CHCl_3$ + 1% pyridine) was used to remove the 1,4-benzoquinone. A large SEC column (toluene + 1% pyridine) was used for separating of the trimer species (15.4 mg, 39%).

^{1}H NMR (700 MHz, $CDCl_3$, 298 K): δ_H (ppm) 9.89 (4H, d, *J* = 4.5 Hz, H8), 9.88 (4H, d, *J* = 4.5 Hz, H9), 9.66 (4H, d, *J* = 4.5 Hz, H2), 9.00 (4H, d, *J* = 4.6 Hz, H7), 8.98 (4H, d, *J* = 4.7 Hz, H10), 8.89 (4H, d, *J* = 4.5 Hz, H3), 8.14 (4H, d, *J* = 1.8 Hz, H11), 8.05 (8H, d, *J* = 1.8 Hz, H4), 7.87 (2H, t, *J* = 1.8 Hz, H13), 7.81 (4H, t, *J* = 1.8 Hz, H6), 1.77–0.90 (78H, m, H1), 1.59 (36H, s, H12), 1.56 (72H, s, H5), –1.51 (2H, s, H14).

^{13}C NMR (100 MHz, $CDCl_3$, 298 K): δ_C (ppm) 153.1, 152.4, 151.0, 150.5, 149.3, 149.1, 141.2, 133.7, 133.2, 131.5, 131.0, 130.2, 129.9, 124.8, 124.4, 121.7, 121.3, 35.3, 35.3, 33.5, 32.0, 31.9, 24.5, 22.9, 14.3, 14.0.

MALDI-TOF: *m/z* = 2893 ($C_{192}H_{230}N_{12}Si_2Zn_2$, M^+ requires 2894).

λ_{max} **($CHCl_3$) / nm log(ε)**: 752 (5.15), 661 (4.96), 485 (5.29), 455 (5.70).

$P3_{Cu}$

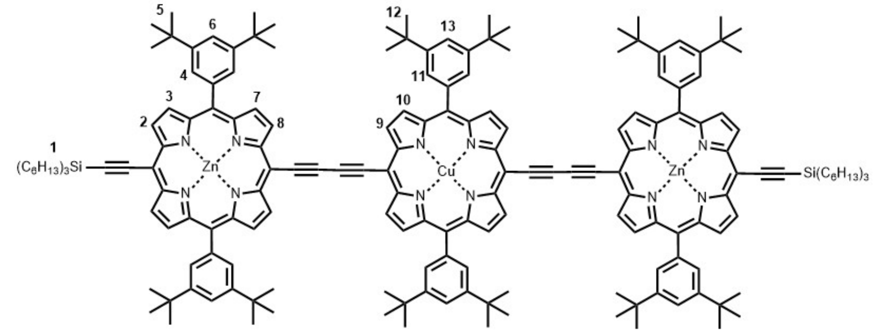

$P3_{2H}$ (4.5 mg, 1.6 µmol) was dissolved in chloroform (4 mL). $Cu(OAc)_2 \cdot H_2O$ (12.2 mg, 67 mmol) was added as a solid and the reaction was stirred at 60˚C for 1 h. The reaction mixture was allowed to cool down to room temperature after which MeOH was added to the reaction mixture to crash out the product. The precipitate was filtered and washed with MeOH to give a red/brown solid (4.8 mg, 100%).

^{1}H NMR (700 MHz, $CDCl_3$, 298 K): δ_H (ppm) 9.87 (4H, m, H8), 9.74 (4H, m, H2), 9.05 (4H, m, H7), 8.96 (4H, m, H3), 8.09 (8H, m, H4), 7.86 (4H, m, H6), 7.75 (2H, m, H13), 1.59 (108H, m, H5 + H12), 1.89–0.80 (78H, m, H1).

MALDI-TOF: *m/z* = 2953 ($C_{192}H_{228}CuN_{12}Si_2Zn_2$, M^+ requires 2954).

λ_{max} **($CHCl_3$) / nm log(ε)**: 726 (5.20), 666 (4.89), 486 (5.37), 454 (5.65).

P5$_{2H}$[12]

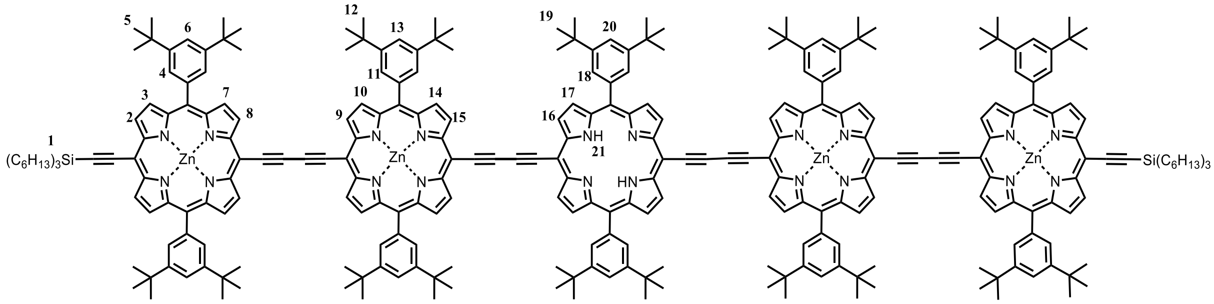

P1"$_{2H}$ (20.0 mg, 0.027 mmol), **P2'** (254.1 mg, 0.14 mmol), $Pd_2(dba)_3$ (12.3 mg, 0.014 mmol), tri-2-furylphosphine (25.2 mg, 0.11 mmol) and 1,4-benzoquinone (37.7 mg, 0.35 mmol) were dissolved in a mixture of toluene and triethylamine (5:1) (37 mL). The reaction mixture was stirred at 60 ˚C overnight. The solvents were removed and the mixture was purified over a plug of silica gel ($CHCl_3$ + 1% pyridine). A small SEC column ($CHCl_3$ + 1% pyridine) was used to remove the 1,4-benzoquinone. Recycling GPC (1% pyridine in toluene) was used for true separation (**P5$_{2H}$**: 49.3 mg, 41%, **P4**: 124.8 mg, 33%).

^{1}H NMR (500 MHz, $CDCl_3$, 298 K): δ_H (ppm) 9.92 (16H, m , H8 + H9 + H15 + H16), 9.68 (4H, d, *J* = 4.5 Hz, H2), 9.02 (16H, m, H7 + H10 + H14 +H17), 8.91 (4H, d, *J* = 4.5 Hz, H3), 8.16 (4H, d, *J* = 1.8 Hz, H18), 8.12 (8H, d, *J* = 1.8 Hz, H11), 8.07 (8H, d, *J* = 1.8 Hz, H4), 7.89 (2H, t, *J* = 1.8 Hz, H20), 7.86 (4H, t, *J* = 1.8 Hz, H13), 7.83 (4H, t, *J* = 1.8 Hz, H6), 1.77–0.91 (78H, m, H1), 1.62 (36H, s, H19), 1.60 (72H, s, H5 or 12), 1.59 (72H, s, H5 or H12), –1.48 (2H, s, H21).
MALDI-TOF: *m/z* = 4485 ($C_{296}H_{330}N_{20}Si_2Zn_4$, M^+ requires 4486).
λ_{max} ($CHCl_3$) / nm log(ε): 783 (5.43), 682 (5.09), 489 (5.64), 458 (5.79).

P5$_{Cu}$

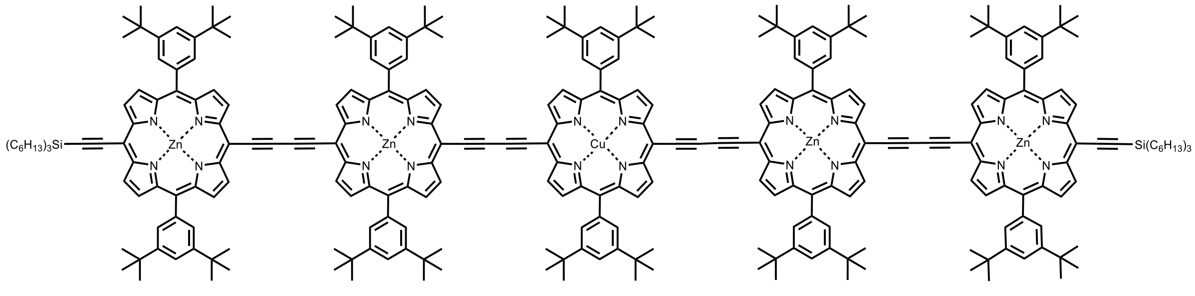

P5$_{2H}$ (40 mg, 10.2 µmol) was dissolved in $CHCl_3$ (10 mL). $Cu(OAc)_2{\cdot}H_2O$ (12.7 mg, 0.383 mmol) was added as a solid and the reaction was stirred at 60˚C for 1 h. The reaction mixture was allowed to cool down to room temperature after which MeOH was added to the reaction mixture to crash out the product. The precipitate was filtered and washed with MeOH to give a red/brown solid (29.5 mg, 73%).

^{1}H NMR (400 MHz, $CDCl_3$, 298 K): δ_H (ppm) 9.90 (m, -ArH_β), 9.67 (d, *J* = 4.4 Hz, -ArH_β), 9.00 (m, -ArH_β), 8.90 (d, *J* = 4.6 Hz, -ArH_β), 7.82 (m, -ArH_{ortho}), 8.08 (m, -ArH_{para}), 1.57 (m, -*t*BuH), 1.77 (m, $-C_6H_{13}$), 1.39 (m, $-C_6H_{13}$), 1.02 (m, $-C_6H_{13}$), 0.90 (m, $-C_6H_{13}$).
MALDI-TOF: *m/z* = 4545 ($C_{296}H_{328}N_{20}CuSi_2Zn_4$, M^+ requires 4547).
λ_{max} ($CHCl_3$) / nm log(ε): 764 (5.45), 693 (5.07), 492 (5.63), 457 (5.75).

$P5''_{Cu}$

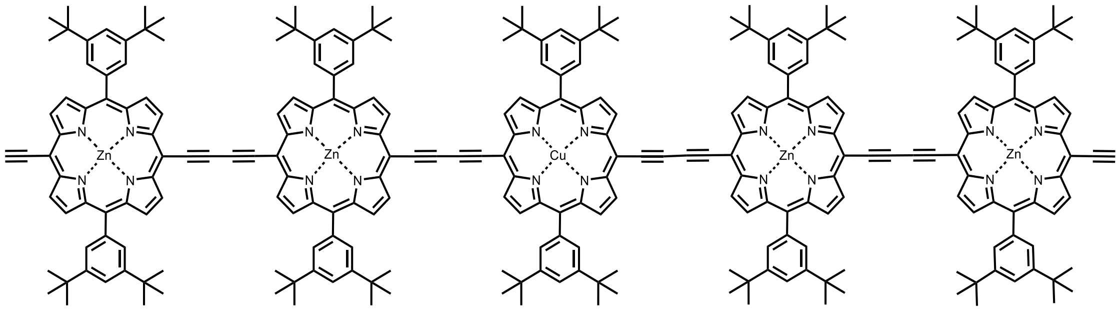

$P5_{Cu}$ (28 mg, 6.16 µmol) was dissolved in CH_2Cl_2 (9 mL) and pyridine (9 µL). Tetra-*n*-butylammonium fluoride solution (1.0 M in THF) (124 µL, 0.123 mmol) was added dropwise to the reaction mixture and was stirred at room temperature for 15 min. After the completion of the reaction, MeOH was added to the reaction mixture to crash out the product. The precipitate was filtered and washed with MeOH to give a red/brown solid (21.1 mg, 86%).

MALDI-TOF: m/z = 3976 ($C_{260}H_{252}CuN_{20}Zn_4$, M^+ requires 3982).

c-$P6_{Cu2}$

Hexadentate template **T6** (81 mg, 81.6 µmol) and **$P3''_{Cu}$** (65 mg, 27.2 µmol) were dissolved in $CHCl_3$ (150 mL). A solution of $PdCl_2(PPh_3)_2$ (9.5 mg, 13.6 µmol), CuI (9.5 mg, 50.3 µmol), 1,4-benzoquinone (29.4 mg, 272 µmol) in $CHCl_3$ (10 mL) and diisopropylamine (0.5 mL) was added to the porphyrin solution and stirred at room temperature overnight. The reaction mixture was passed through a plug of alumina using $CHCl_3$ as eluent. The solvent was evaporated and redissolved in 1% pyridine in chloroform and passed over a SEC column (1% pyridine in chloroform). Lastly, the ring was purified by recycling GPC (1% pyridine in toluene) to give the title compound (1.8 mg, 2%).

^{1}H NMR (600 MHz, $CDCl_3$, 298 K): δ_H (ppm) 9.71–9.31 (m, -ArH_β), 8.93–8.68 (m, -ArH_β), 8.05 (s, -ArH_{ortho}), 7.83 (s, -ArH_{para}), 7.78 (s, -ArH_{para}), 5.77 (m, T6), 5.68 (m, T6), 5.14 (m, T6), 2.34 (m, T6), 2.02 (m, T6), 1.59 (s, -*t*BuH).
MALDI-TOF: m/z = 4787 ($C_{320}H_{300}Cu_2N_{24}Zn_4$, M^+ requires 4774).
λ_{max} ($CHCl_3$) / nm log(ε): 480 (5.67), 761 (5.47), 797 (5.58), 838 (5.55).

c-P10$_{Cu2}$

Pentadentate template **T5** (5.10 mg, 6.04 µmol) and **P5”$_{Cu}$** (8.02 mg, 2.01 µmol) were dissolved in $CHCl_3$ (8 mL). A solution of $PdCl_2(PPh_3)_2$ (0.71 mg, 1.01 µmol), CuI (0.71 mg, 3.73 µmol), 1,4-benzoquinone (2.18 mg, 20.1 µmol) in $CHCl_3$ (0.7 mL) and diisopropylamine (35 µL) was added to the porphyrin solution and stir at room temperature overnight. The reaction mixture was passed through a plug of alumina using $CHCl_3$ as eluent. The solvent was evaporated and redissolved in 20% pyridine in chloroform and passed over a SEC column (20% pyridine in chloroform) to remove the template. Lastly, the ring was purified by recycling GPC (1% pyridine in toluene) to give the title compound (1.33 mg, 17%).

^{1}H NMR (500 MHz, $CDCl_3$, 298 K): δ_H (ppm) 9.87–9.77 (m, -ArH_β), 9.02–8.85 (m, -ArH_β), 8.08–7.98 (m, -ArH_{ortho}), 7.84–7.77 (m, -ArH_{para}), 6.58 (s, bound pyridine), 5.86 (s, bound pyridine), 3.77 (s, bound pyridine), 1.55 (s, -*t*BuH$_{(Cu)}$), 1.54 (s, -*t*BuH$_{(Zn)}$).
MALDI-TOF: *m*/*z* = 7960 ($C_{520}H_{500}Cu_2N_{40}Zn_8$, M^+ requires 7960).
λ_{max} ($CHCl_3$) / nm log(ε): 806 (5.67), 771 (5.67), 495 (5.91).

Tetradentate template T4

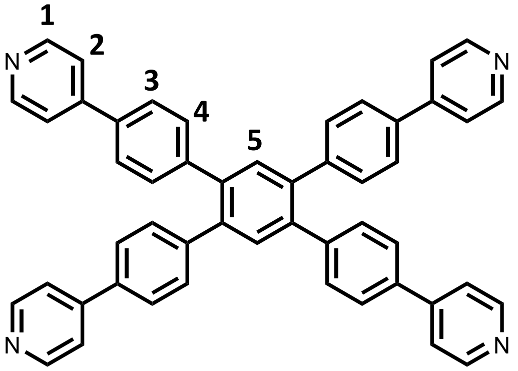

Under an argon atmosphere 1,2,4,5-tetrabromobenzene (52 mg, 0.13 mmol), 4-[4-(tetramethyl-1,3,2-dioxaborolan-2-yl)phenyl]pyridine (370 mg, 1.04 mmol), $Pd(OAc)_2$ (6.0 mg, 0.03 mmol), SPhos (21.7 mg, 0.05 mmol) and Cs_2CO_3 (859 mg, 2.60 mmol) were dissolved in toluene (3.25 mL), EtOH (0.65 mL) and H_2O (0.65 mL). Oxygen was removed from the reaction mixture by three freeze-pump-thaw cycles after which the mixture was heated at 70 ˚C for 16 h. TLC (DCM:MeOH:Et_3N = 8:1:0.1) indicated the full consumption of the starting material, the mixture was allowed to cool to room temperature after which the product was extracted with $CHCl_3$ and washed with H_2O and brine. The organic layer was dried over $MgSO_4$ and concentrated in *vacuo*. The off-white solid was washed with MeOH and purified further by silica gel column chromatography (dry loading, DCM:MeOH:Et_3N = 100:1:0.25 → 100:6:0.5) to give **T4** as a pale white powder (45 mg, 49%).

^{1}H NMR (400 MHz, $CDCl_3$, 298 K): δ_H (ppm) 8.66 (8H, d, *J* = 5.9 Hz, H1), 7.66 (2H, s, H5), 7.60 (8H, d, *J* = 8.3 Hz, H3), 7.52 (8H, d, *J* = 5.9 Hz, H2), 7.41 (8H, d, *J* = 8.3 Hz, H4).
^{13}C NMR (100 MHz, CDCl3, 298 K): δ_C (ppm) 150.7, 147.9, 141.8, 139.7, 137.0, 133.5, 131.0, 127.2, 121.7.
MALDI-TOF: *m*/*z* = 691 ($C_{50}H_{34}N_4$, M^+ requires 690).

Section S7. Spectra Confirming Identity of New Compounds

$P1_{2H}$

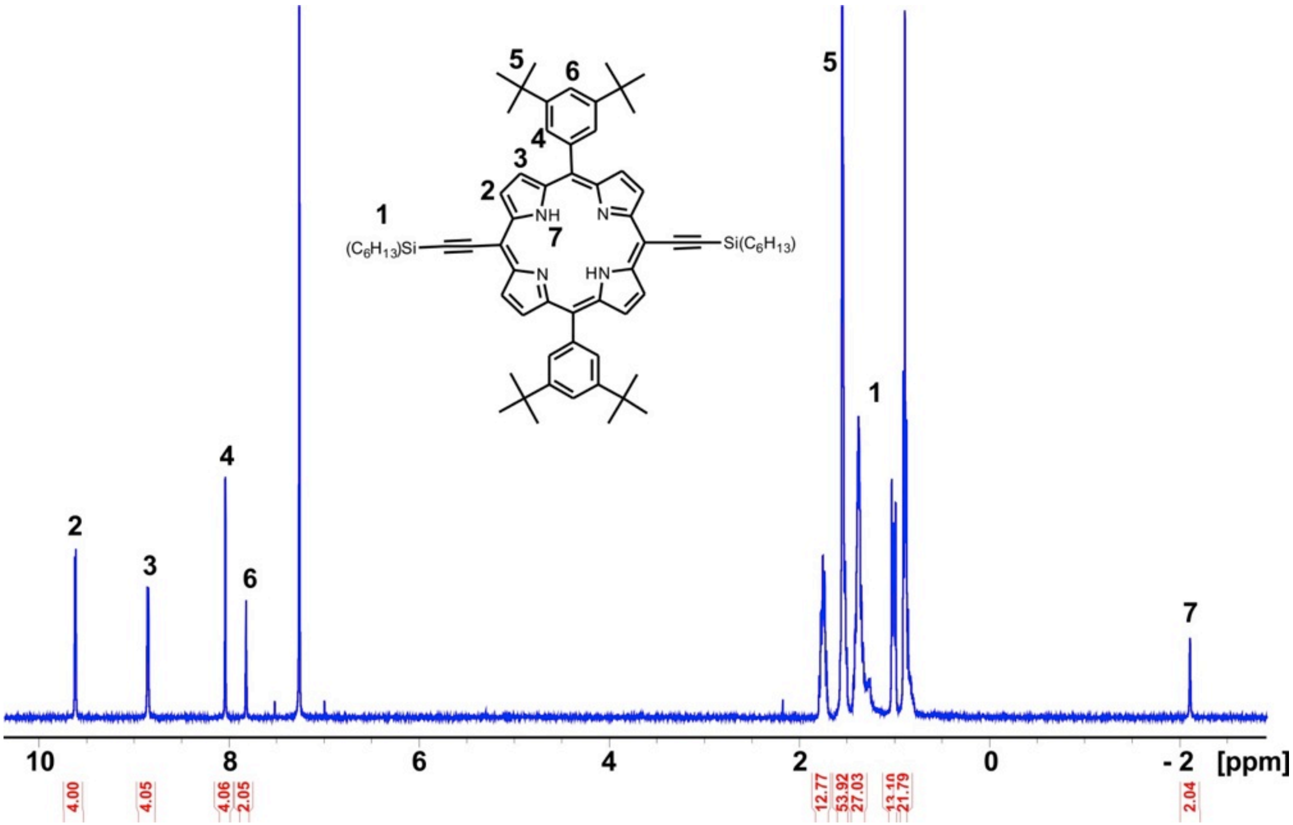

Figure S37: The ^1H NMR spectrum of $P1_{2H}$ (400 MHz, $CDCl_3$).

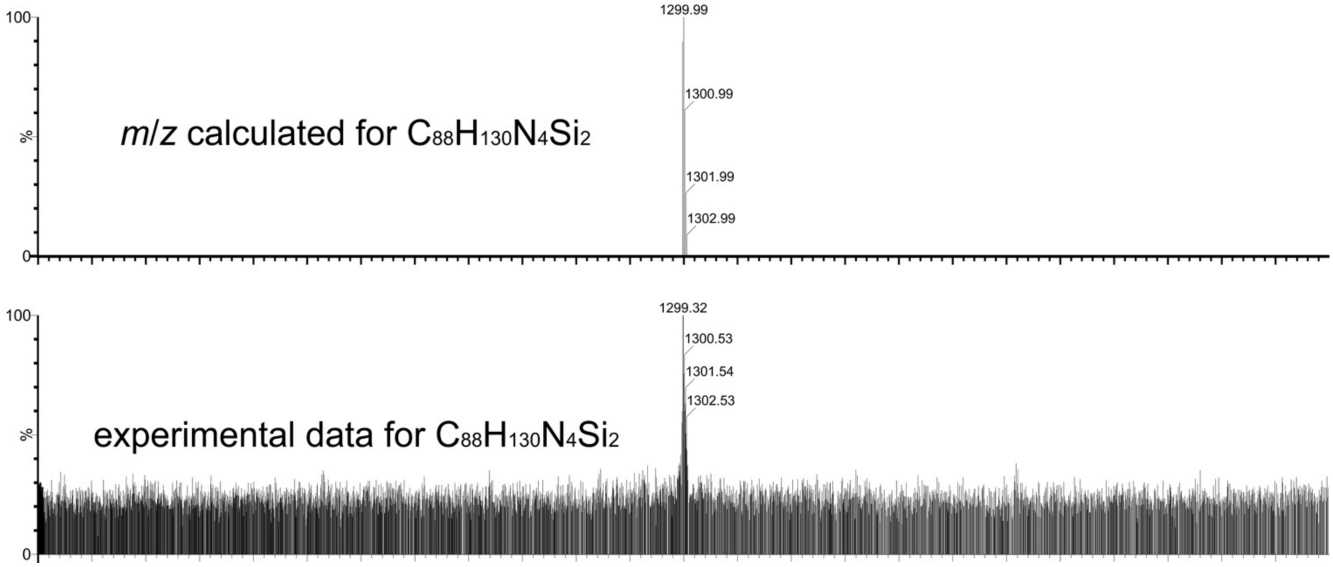

Figure S38: The MALDI-MS spectrum of $P1_{2H}$ (m/z = 1299 ($C_{88}H_{130}N_4Si_2$, M^+ requires 1300), matrix: dithranol).

P1$_{Cu}$

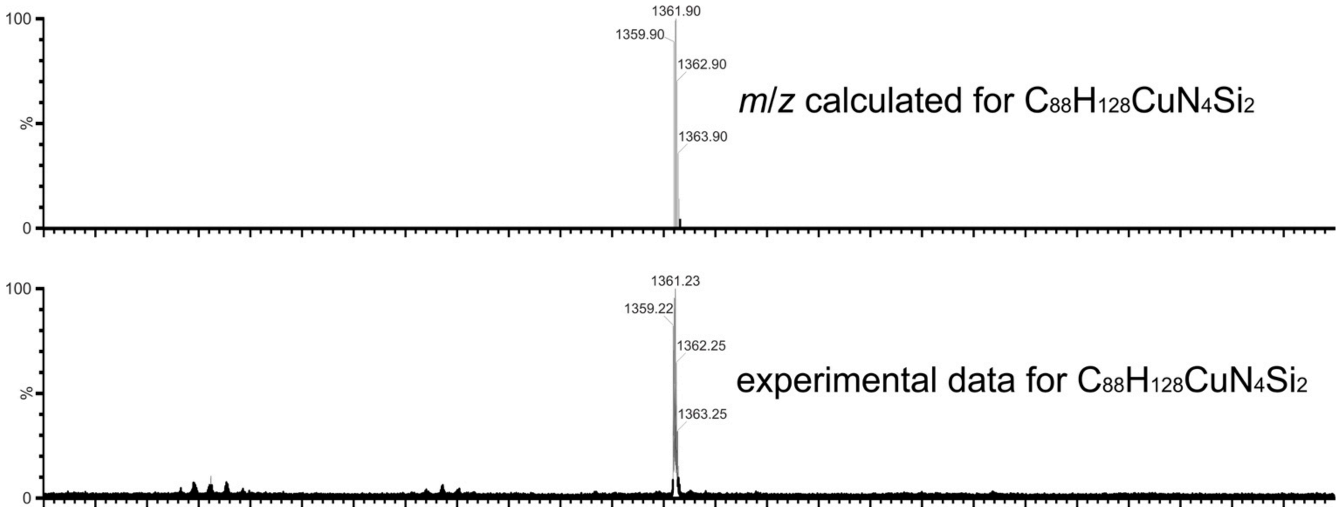

Figure S39: The MALDI-MS spectrum of P1$_{Cu}$ (m/z = 1361 ($C_{88}H_{128}CuN_4Si_2$, M^+ requires 1362), matrix: dithranol).

P2'

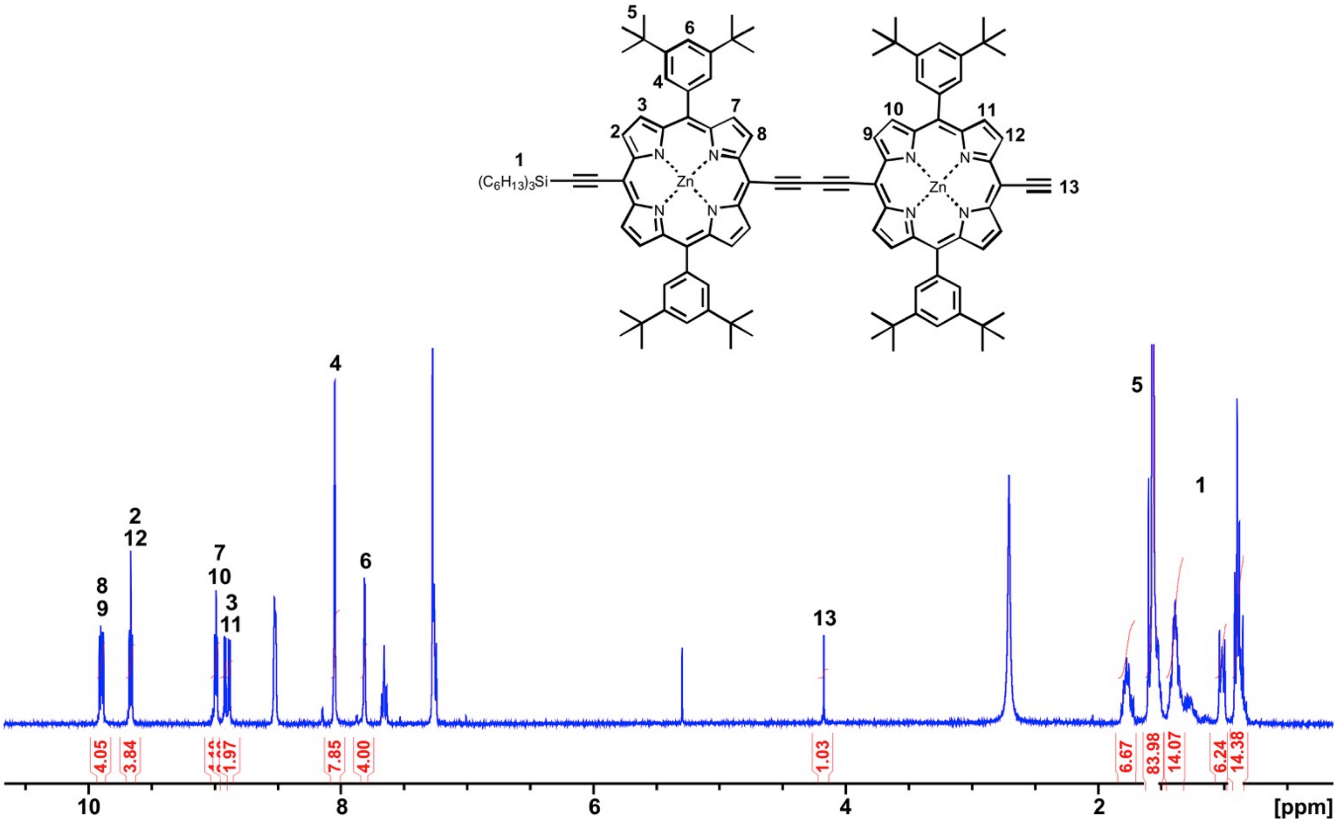

Figure S40: The ^{1}H NMR spectrum of P2' (400 MHz, $CDCl_3$ + 1% pyridine-d_5).

P1''$_{2H}$

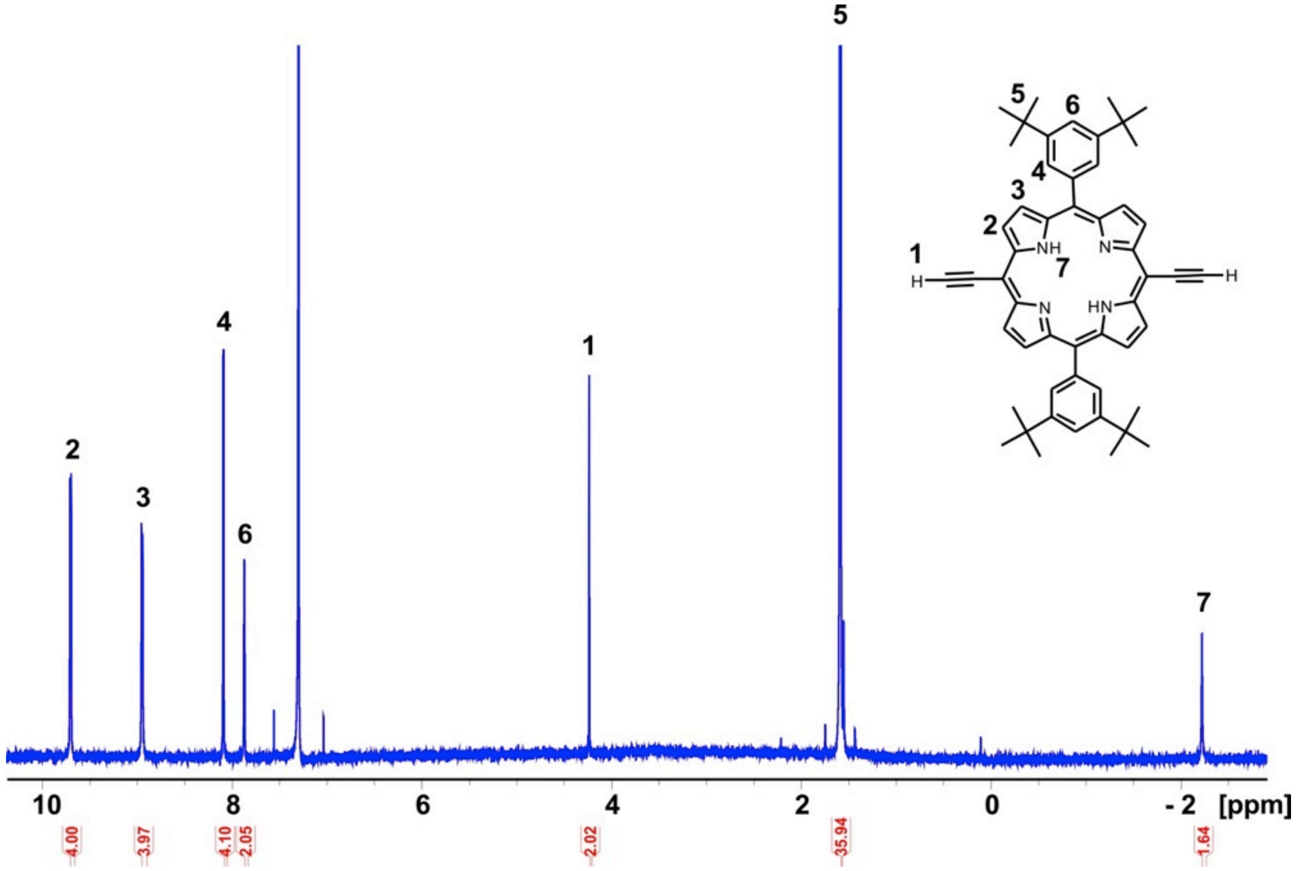

Figure S41: The ^{1}H NMR spectrum of P1''$_{2H}$ (400 MHz, $CDCl_3$).

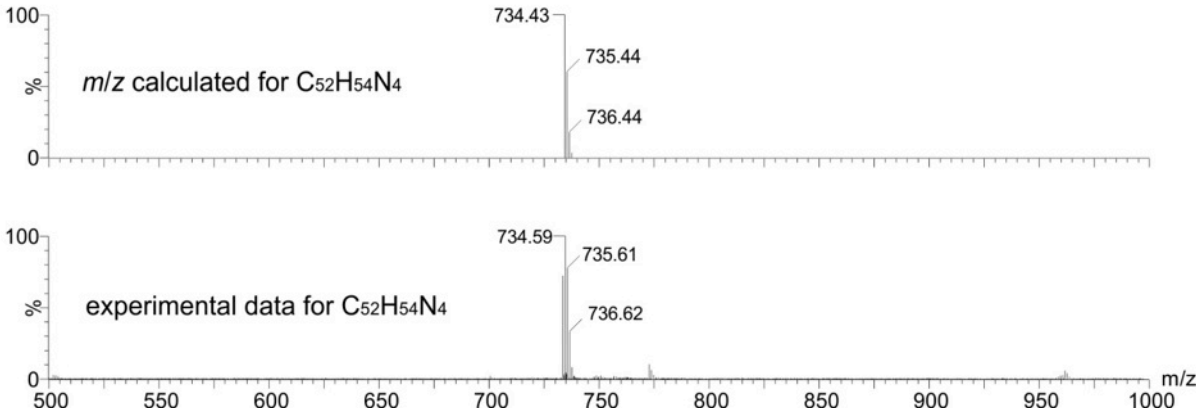

Figure S42: The MALDI-MS spectrum of P1''$_{2H}$ (m/z = 734 ($C_{52}H_{54}N_4$, M^+ requires 735), matrix: dithranol).

$P3_{2H}$

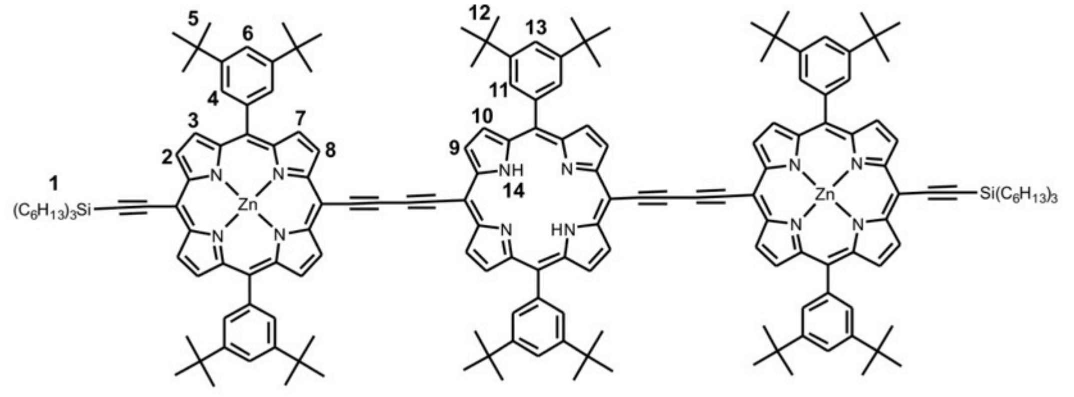

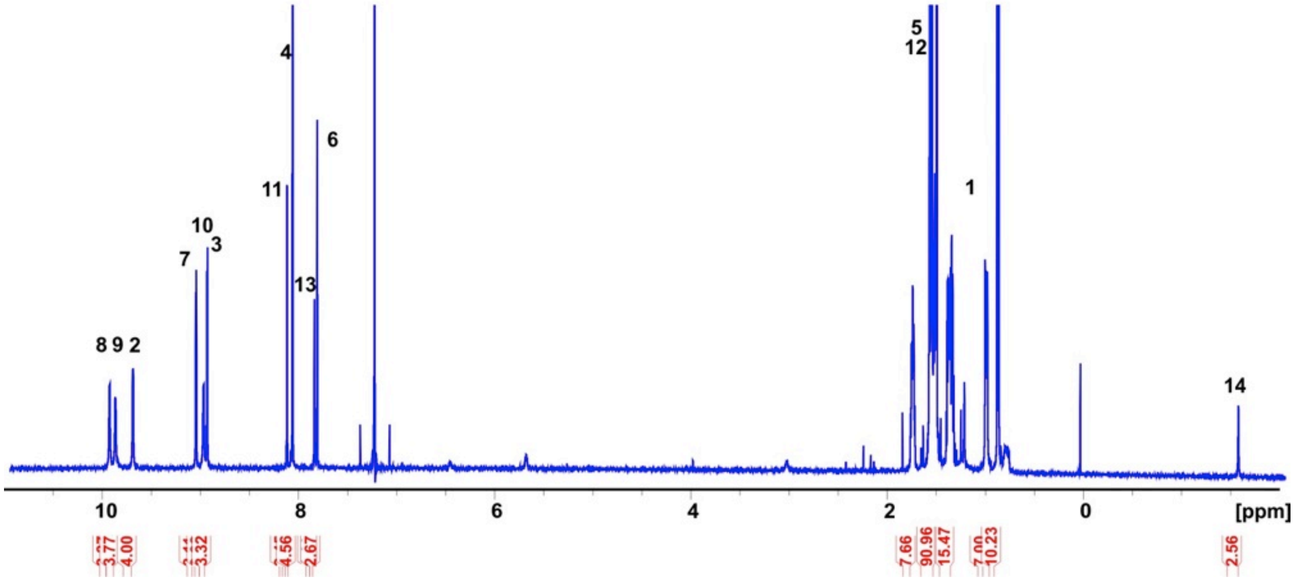

Figure S43: The ^{1}H NMR spectrum of $P3_{2H}$ (700 MHz, $CDCl_3$).

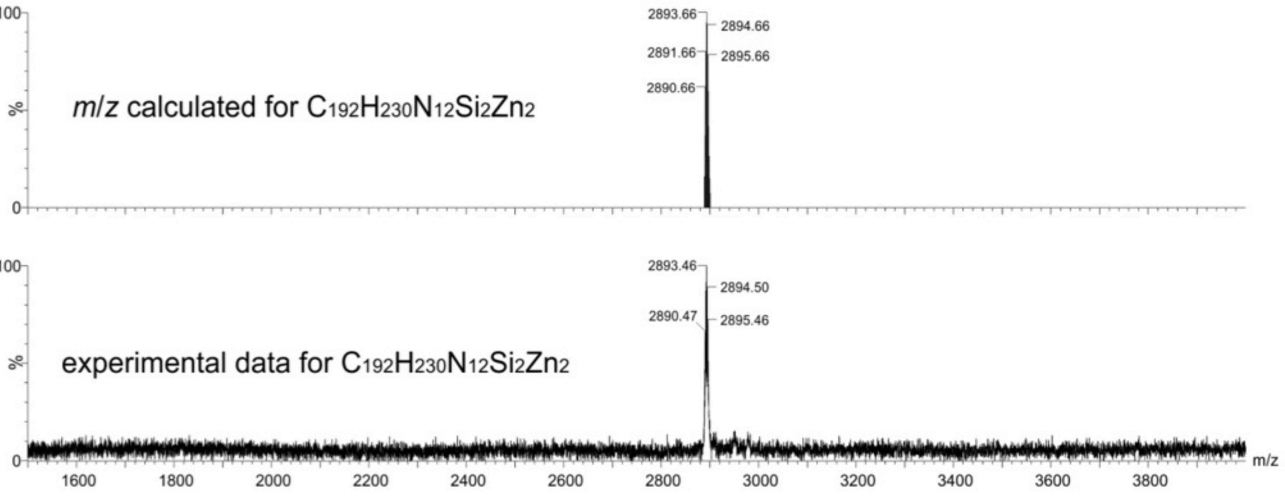

Figure S44: The MALDI-MS spectrum of $P3_{2H}$ (m/z = 2893 ($C_{192}H_{230}N_{12}Si_2Zn_2$, M^+ requires 2894), matrix: dithranol).

$P3_{Cu}$

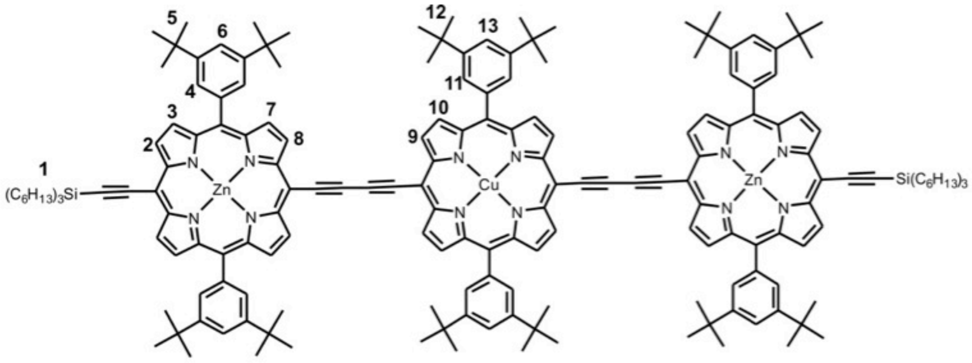

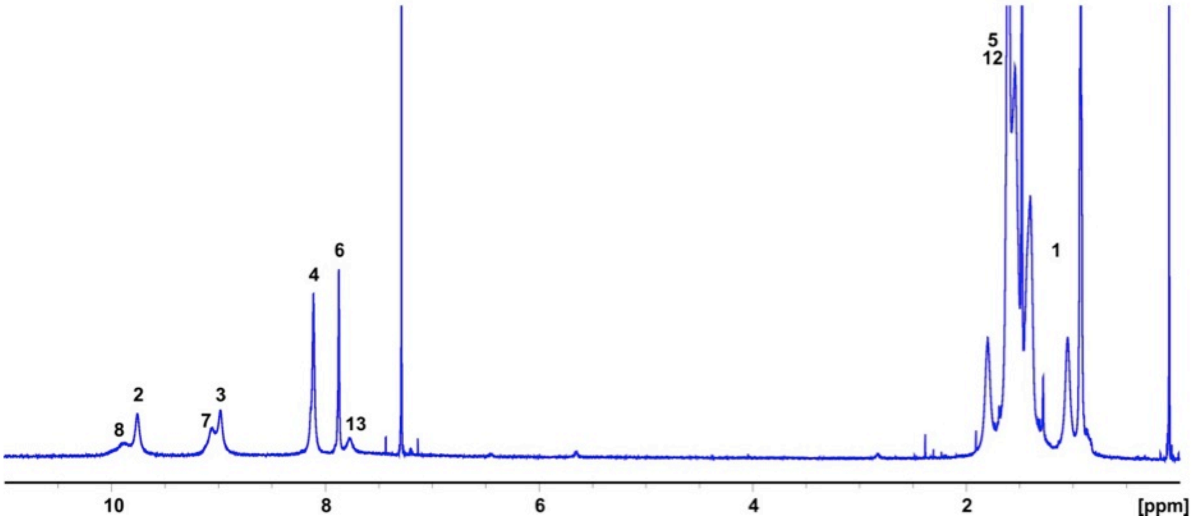

Figure S45: The ^{1}H NMR spectrum of $P3_{Cu}$ (700 MHz, $CDCl_3$).

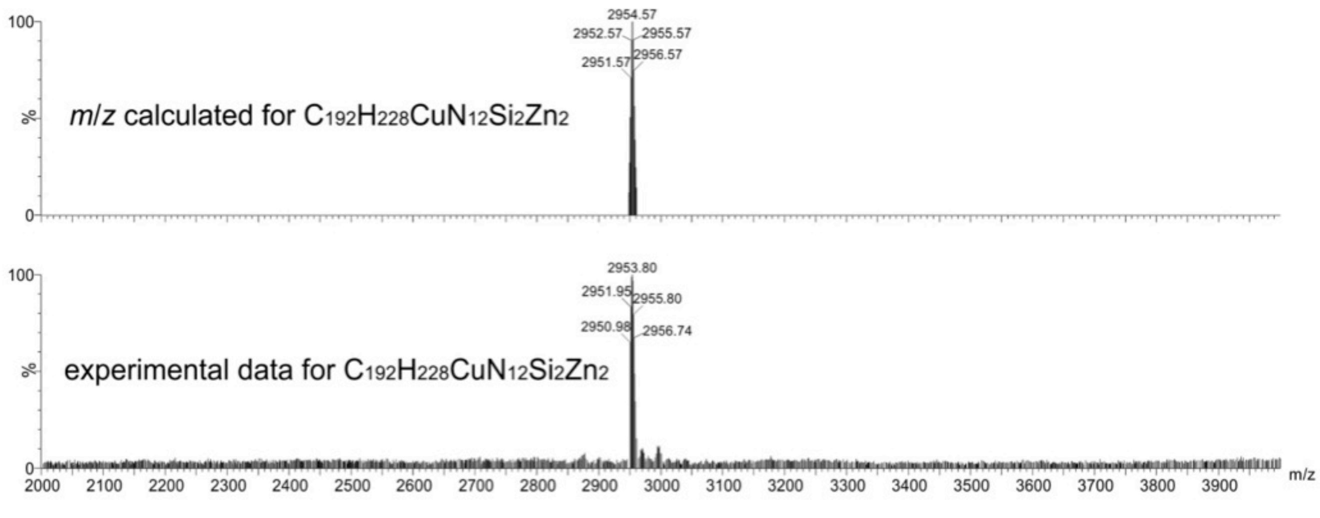

Figure S46: The MALDI-MS spectrum of $P3_{Cu}$ (*m/z* = 2954 ($C_{192}H_{228}CuN_{12}Si_2Zn_2$, M^+ requires 2955), matrix: dithranol).

P5$_{2H}$

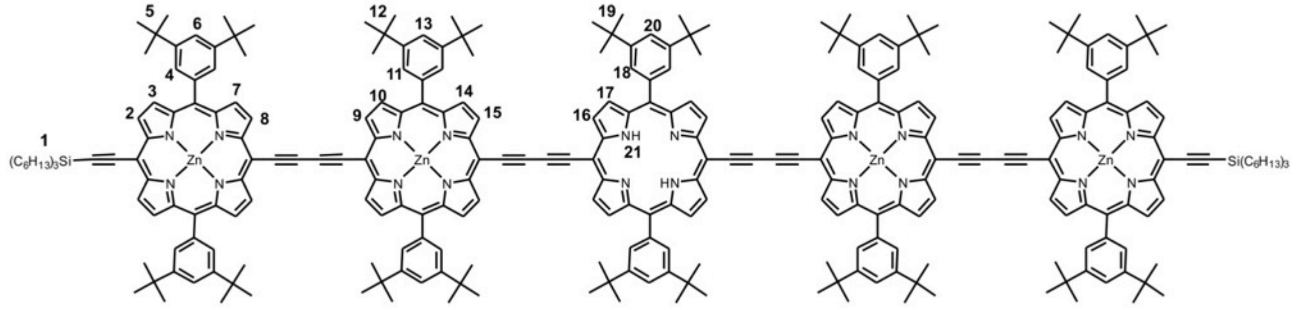

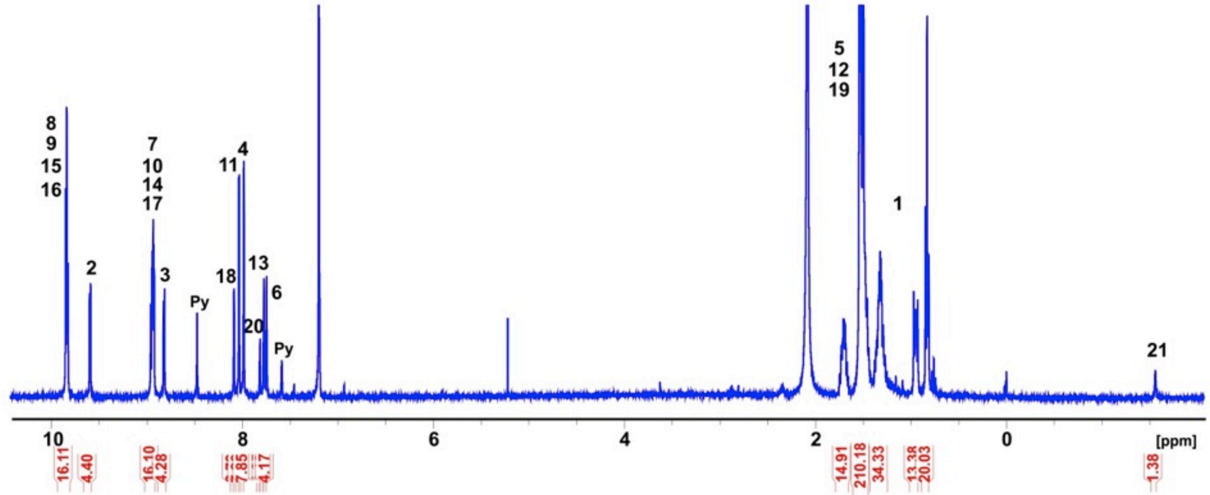

Figure S47: The ^{1}H NMR spectrum of P5$_{2H}$ (500 MHz, $CDCl_3$ + 1% d_5 pyridine).

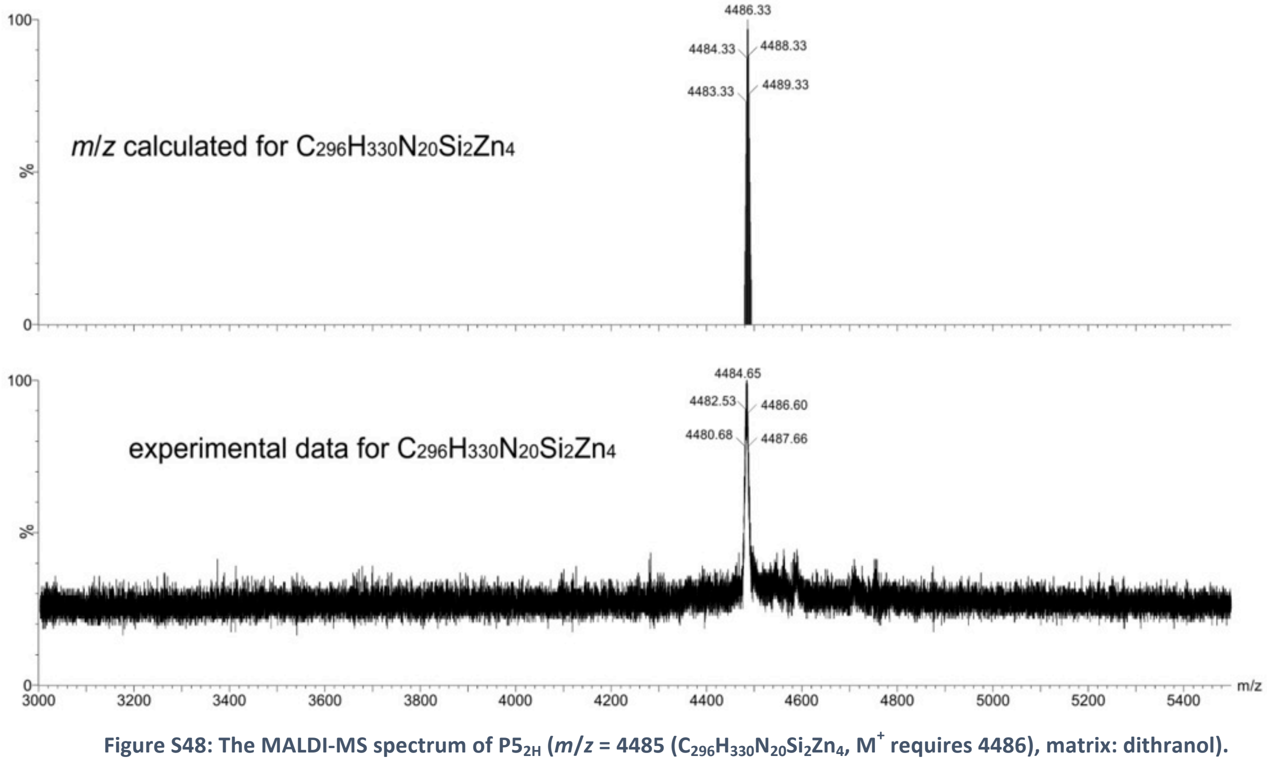

Figure S48: The MALDI-MS spectrum of P5$_{2H}$ (m/z = 4485 ($C_{296}H_{330}N_{20}Si_2Zn_4$, M^+ requires 4486), matrix: dithranol).

P5$_{Cu}$

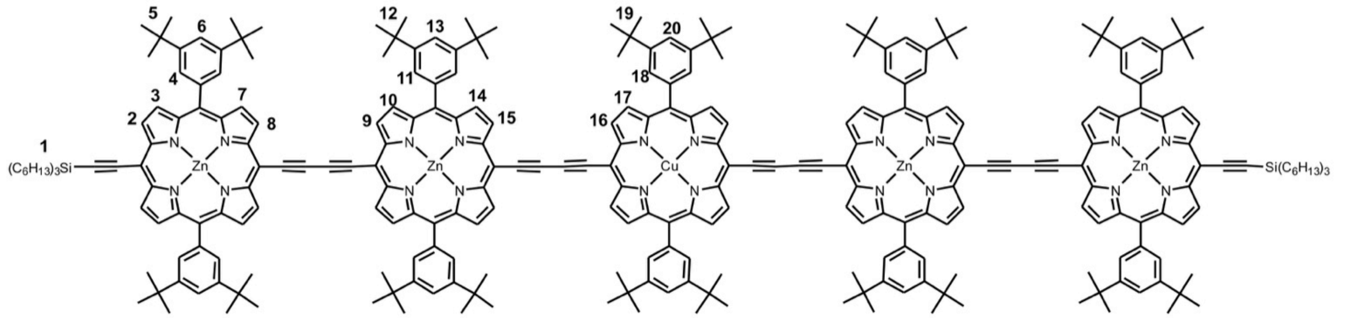

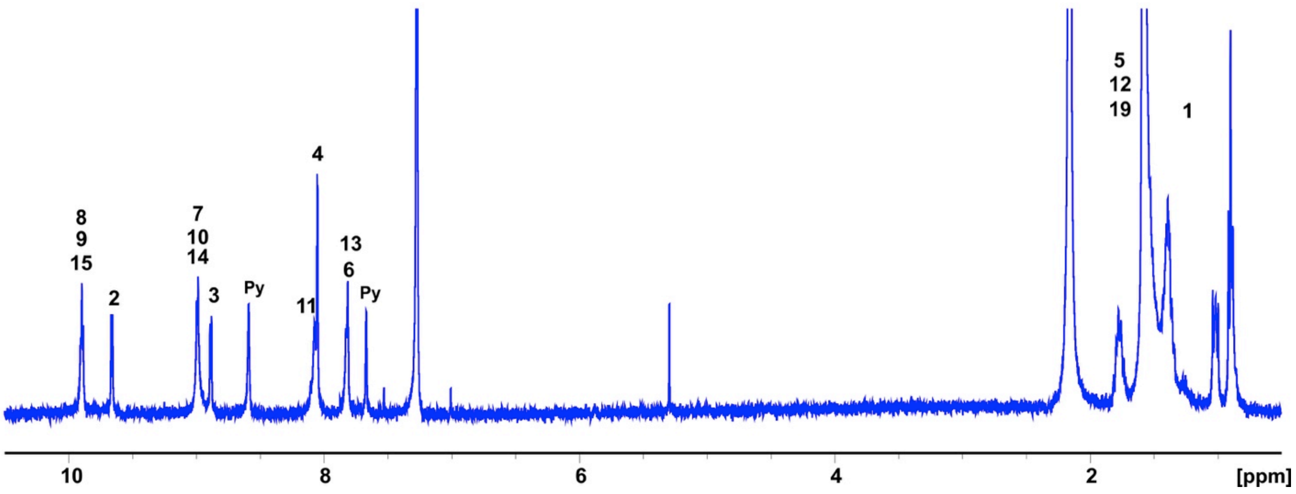

Figure S49: The ^{1}H NMR spectrum of P5$_{Cu}$ (500 MHz, $CDCl_3$ + 1% d_5 pyridine).

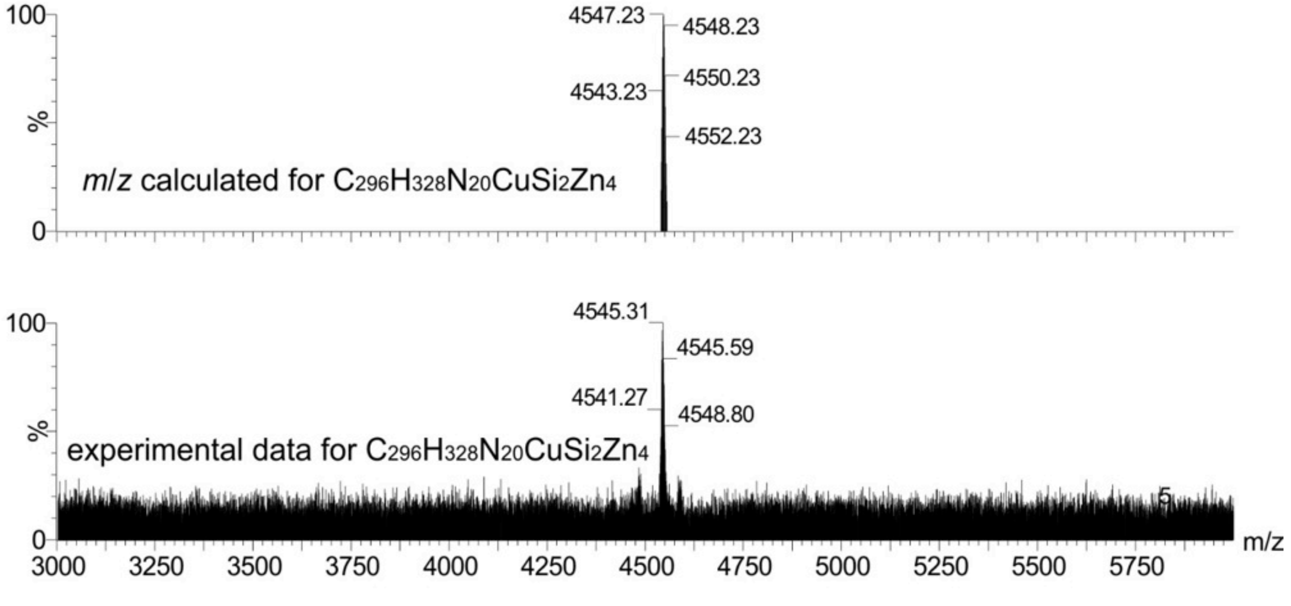

Figure S50: The MALDI-MS spectrum of P5$_{Cu}$ (m/z = 4545 ($C_{296}H_{328}CuN_{20}Si_2Zn_4$, M^+ requires 4547), matrix: dithranol).

T4

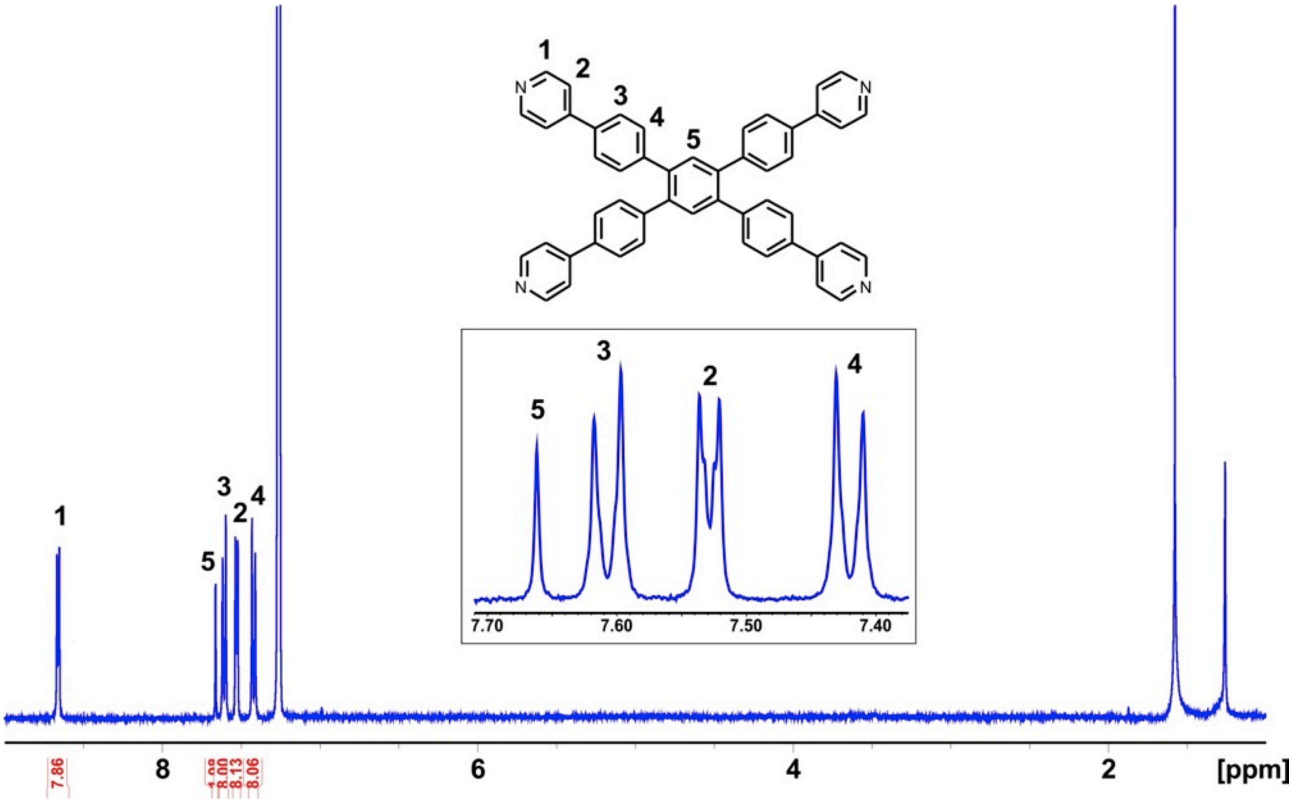

Figure S51: The ^{1}H NMR spectrum of T4 (400 MHz, $CDCl_3$). The signals at δ 1.59 ppm and δ 1.26 ppm correspond to water and grease respectively.

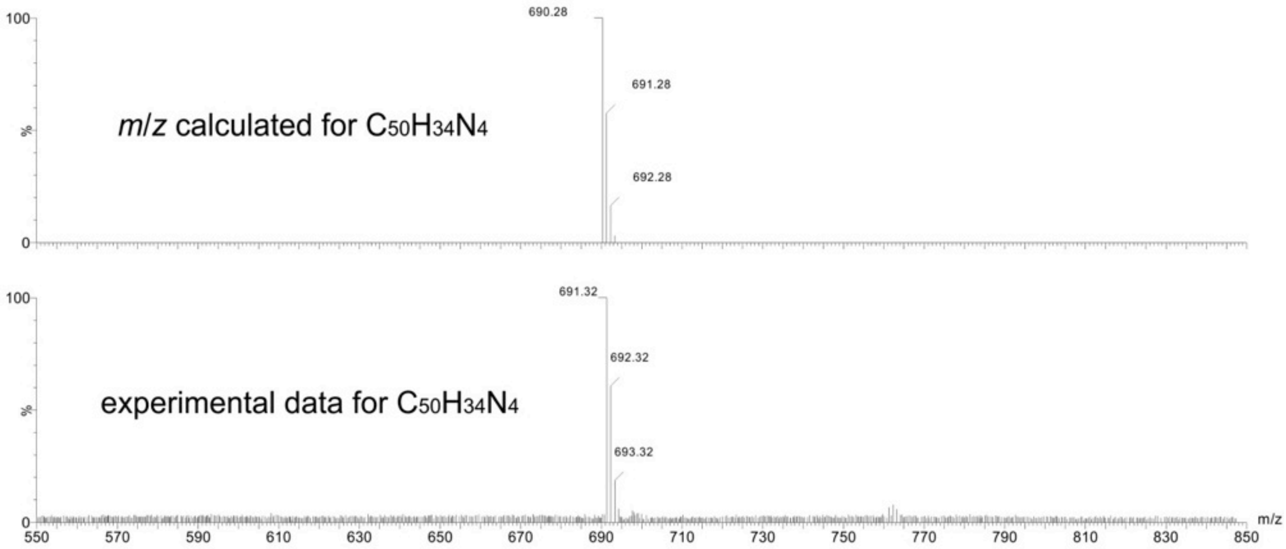

Figure S52: The MALDI-MS spectrum of T4 (m/z = 691 ($C_{50}H_{34}N_4$, M^+ requires 690), matrix: dithranol).

c-$P6_{Cu2}$·T6

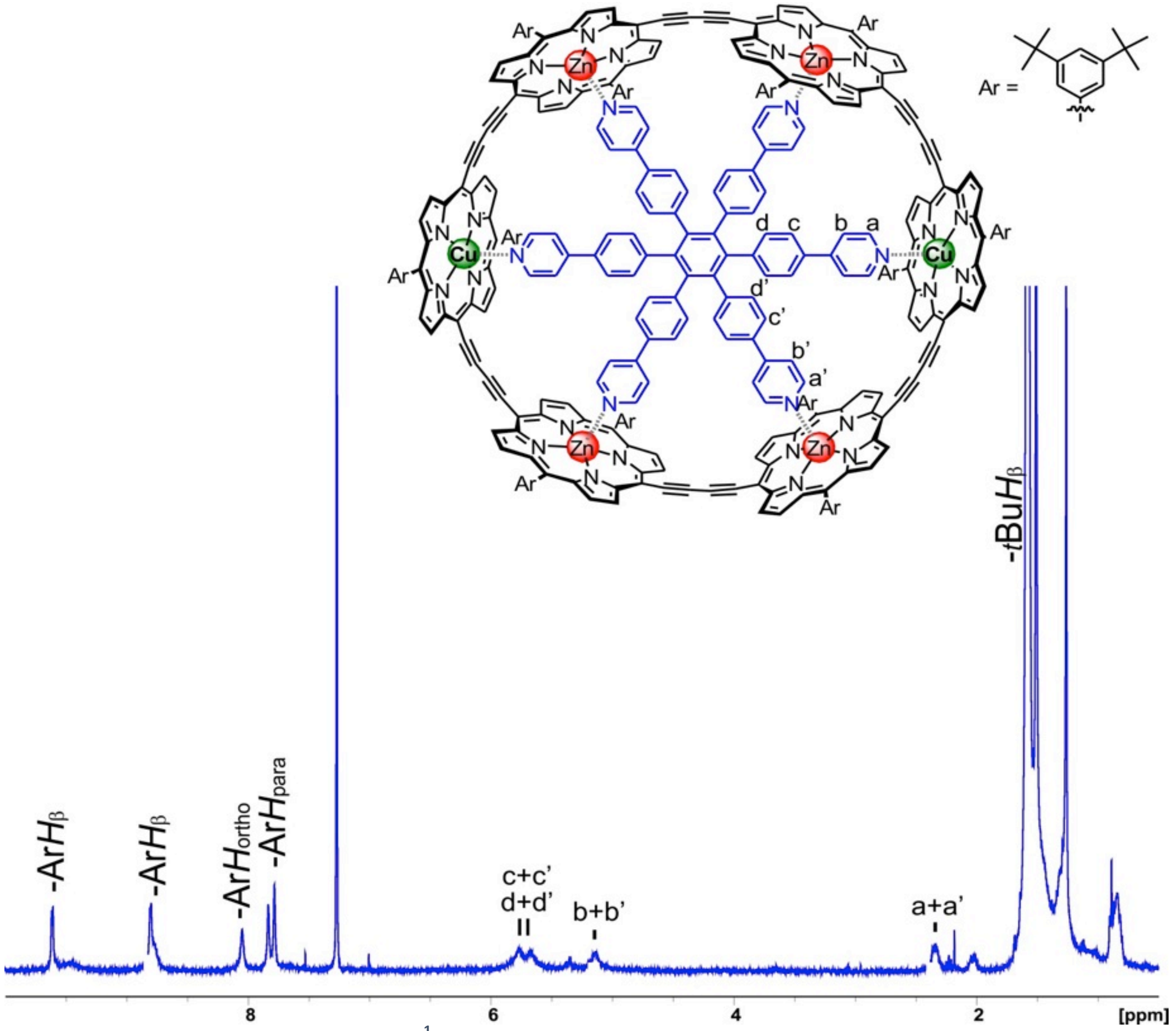

Figure S53: The ^{1}H NMR spectrum of *c*-$P6_{Cu2}$·T6 (600 MHz, $CDCl_3$).

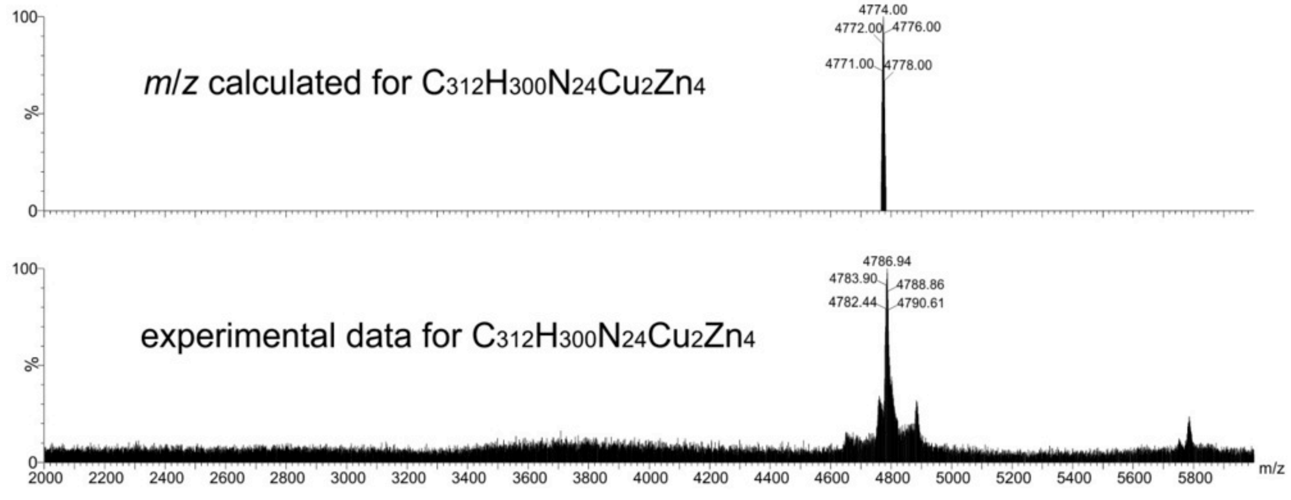

Figure S54: The MALDI-MS spectrum of *c*-$P6_{Cu2}$ (*m/z* = 4787 ($C_{312}H_{300}Cu_2N_{24}Zn_4$, M^+ requires 4774). matrix: DCTB).

c-P10$_{Cu2}$

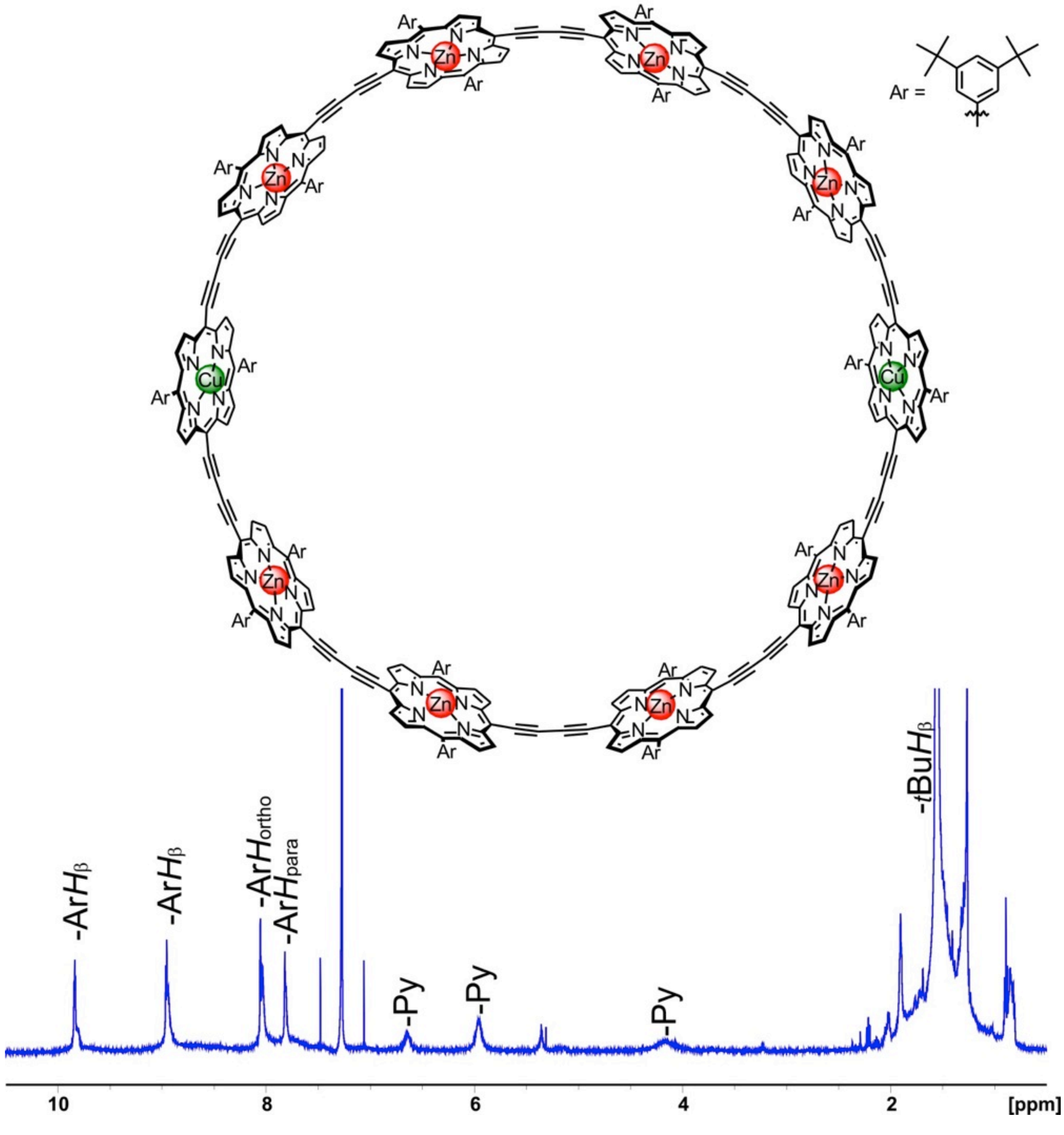

Figure S55: The ¹H NMR spectrum of *c*-P10$_{Cu2}$ (500 MHz, $CDCl_3$). The zinc porphyrins are coordinating an axial pyridine, the signals corresponding to pyridine are denoted as Py in the ¹H NMR spectrum.

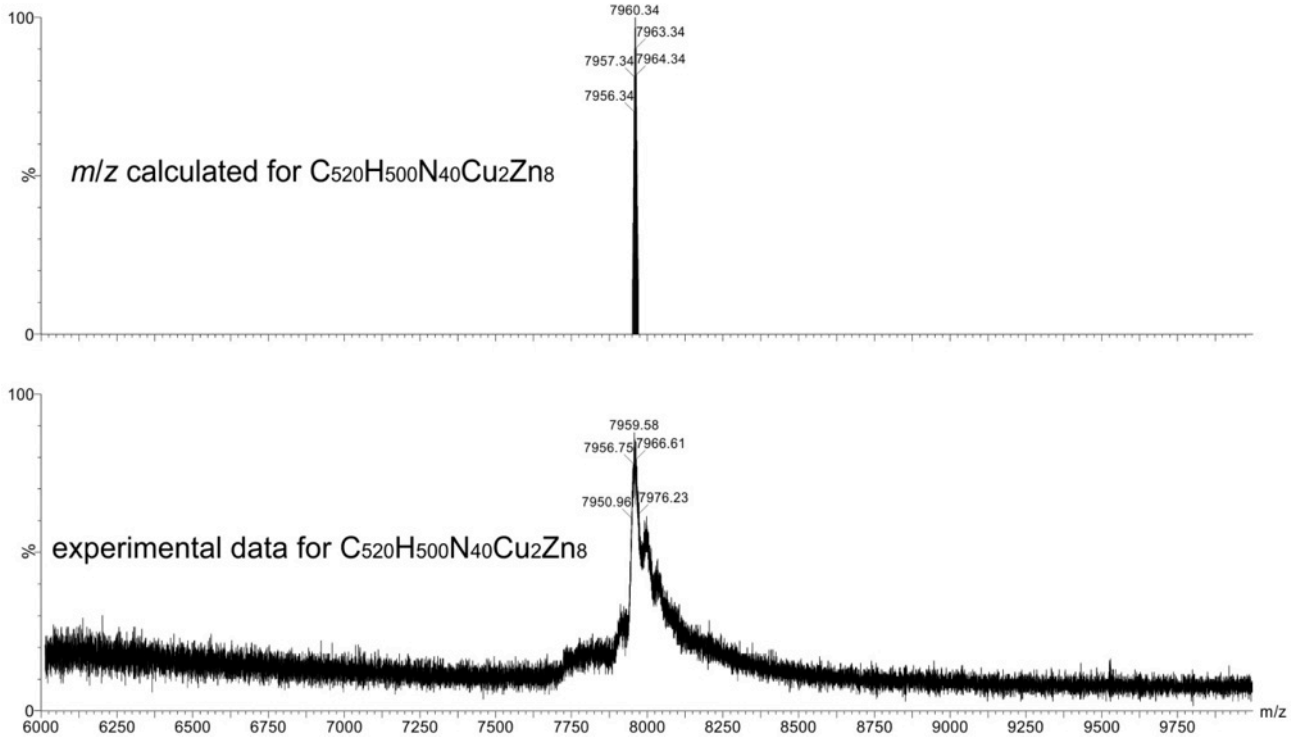

Figure S56: The MALDI-MS spectrum of *c*-P10$_{Cu2}$ (*m/z* = 7960 ($C_{520}H_{500}Cu_2N_{40}Zn_8$, M^+ requires 7960). matrix: DCTB).

Section S8. ^{1}H NMR spectra of 1:1 complexes of $P5_{Cu}$ and $P5_{2H}$ with T4 and T5

The ^{1}H NMR spectra shown below illustrate the chemically pure 1:1 complexes of the linear porphyrin oligomers **$P5_{Cu}$** and **$P5_{2H}$** mixed with 1 equivalent **T4** or **T5**. The spectrum of **$P5_{2H}$·T4** could be assigned and the spectrum for **$P5_{Cu}$·T4** was assigned by analogy. Due to the presence of the paramagnetic copper(II) center, broadening is observed in both the porphyrin oligomer and the template signals in close proximity to the copper. The template complexes with **T5** were found to be more complex and dynamic and could therefore not be assigned. We contemplate that the higher complexity is due to the extra template leg (reducing the symmetry of the template) and the fact that the central leg of the template can either be pointing towards the central porphyrin or point the other way (towards the trihexylsilyl protecting groups) resulting in a larger amount of signals leading to higher complexity in the NMR spectra.

$P5_{2H}$·T4

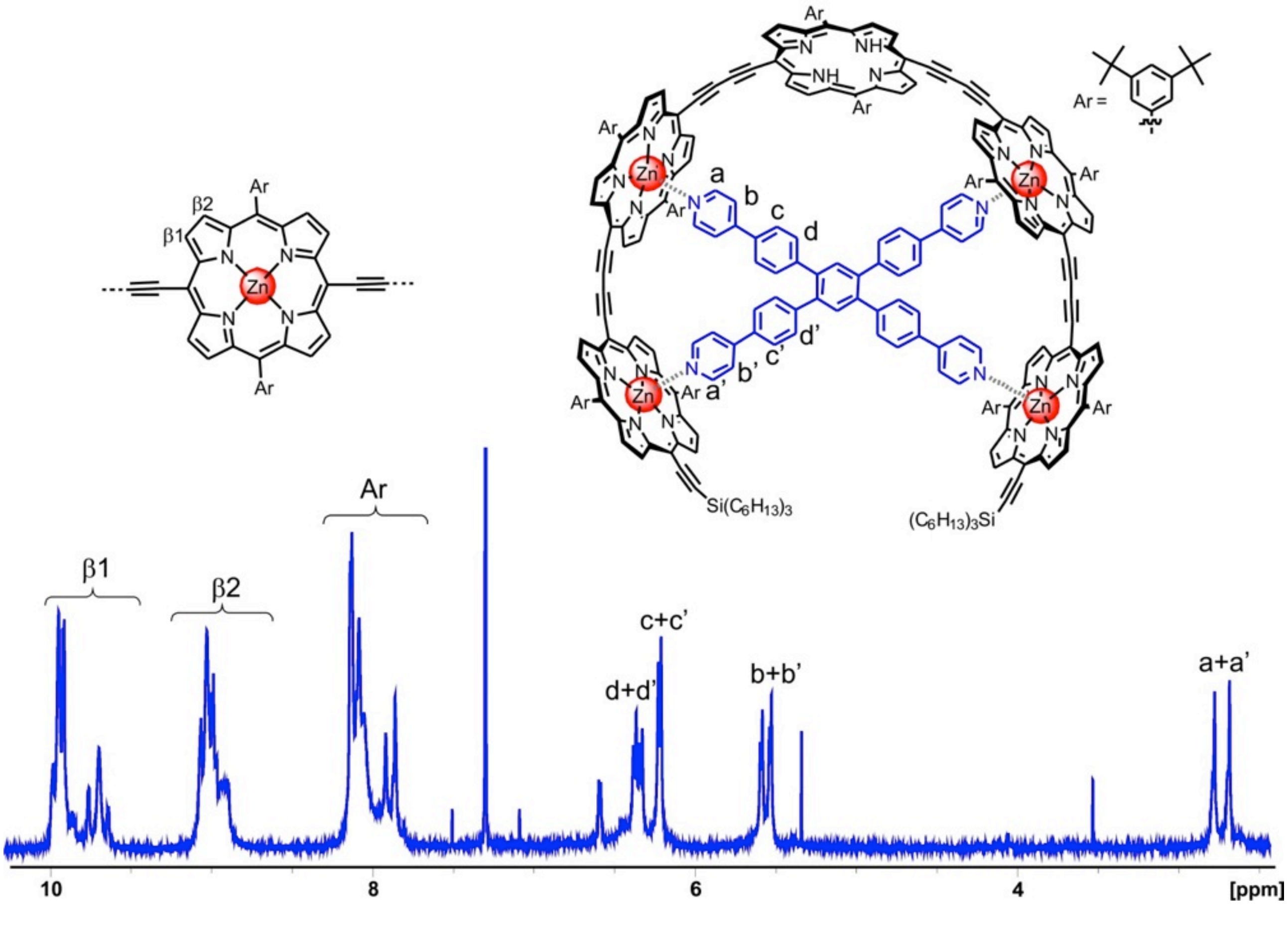

Figure S57: The ^{1}H NMR spectrum of $P5_{2H}$·T4 (500 MHz, $CDCl_3$).

$P5_{Cu}·T4$

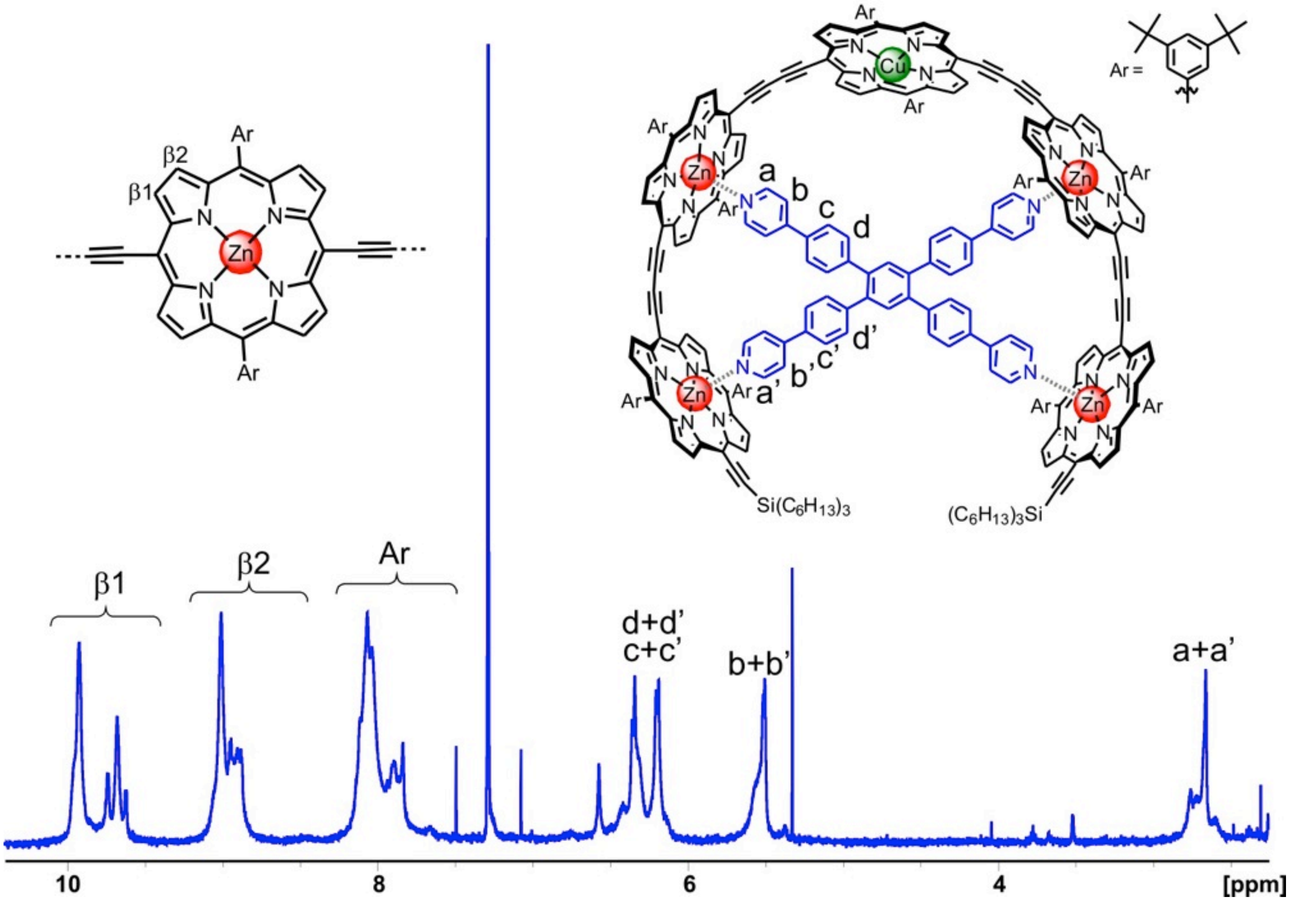

Figure S58: The ^{1}H NMR spectrum of $P5_{Cu}·T4$ (500 MHz, $CDCl_3$).

Section S9. References

(1) H. J. Hogben, J. K. Sprafke, M. Hoffmann, M. Pawlicki and H. L. Anderson, *J. Am. Chem. Soc.*, 2011, **133**, 20962.

(2) P. Liu, P. Neuhaus, D. V. Kondratuk, T. S. Balaban and H. L. Anderson, *Angew. Chem. Int. Ed.*, 2014, **53**, 7770.

(3) C. A. Hunter and H. L. Anderson, *Angew. Chem. Int. Ed.*, 2009, **48**, 7488.

(4) S. W. Benson, *J. Am. Chem. Soc.*, 1958, **80**, 5151.

(5) G. Ercolani, C. Piguet, M. Borkovec and J. Hamacek, *J. Phys. Chem. B*, 2007, **111**, 12195.

(6) R. Ahlrichs, M. Bär, M. Häser, H. Horn and C. Kölmel, *Chem. Phys. Lett.*, 1989, **162**, 165.

(7) A. Schäfer, C. Huber and R. Ahlrichs, *J. Chem. Phys.*, 1994, **100**, 5829.

(8) K. Eichkorn, F. Weigend, O. Treutler and R. Ahlrichs, *Theor. Chem. Acc.*, 1997, **97**, 119.

(9) S. Grimme, *J. Comput. Chem,* 2004, **25**, 1463.

(10) S. Grimme, *J. Comput. Chem.*, 2006, **27**, 1787.

(11) J. K. Sprafke, D. V. Kondratuk, M. Wykes, A. L. Thompson, M. Hoffmann, R. Drevinskas, W.-H. Chen, C. K. Yong, J. Kärnbratt, J. E. Bullock, M. Malfois, M. R. Wasielewski, B. Albinsson, L. M. Herz, D. Zigmantas, D. Beljonne and H. L. Anderson, *J. Am. Chem. Soc.*, 2011, **133**, 17262.

(12) R. W. Wagner, T. E. Johnson, F. Li and J. S. Lindsey, *J. Org. Chem.*, 1995, **60**, 5266.
